# LED-pump-X-ray-multiprobe crystallography for sub-second timescales

**DOI:** 10.1038/s42004-022-00716-1

**Published:** 2022-08-26

**Authors:** Lauren E. Hatcher, Mark R. Warren, Jonathan M. Skelton, Anuradha R. Pallipurath, Lucy K. Saunders, David R. Allan, Paul Hathaway, Giulio Crevatin, David Omar, Ben H. Williams, Ben A. Coulson, Chick C. Wilson, Paul R. Raithby

**Affiliations:** 1 Department of Chemistry, University of Bath, Bath, UK; 2 School of Chemistry, Cardiff University, Cardiff, UK; 3 Diamond Light Source, Harwell Science and Innovation Campus, Didcot, UK; 4 Department of Chemistry, University of Manchester, Manchester, UK; 5 School of Chemical and Process Engineering, University of Leeds, Leeds, UK

**Keywords:** Photochemistry, X-ray crystallography, Characterization and analytical techniques

## Abstract

The visualization of chemical processes that occur in the solid-state is key to the design of new functional materials. One of the challenges in these studies is to monitor the processes across a range of timescales in real-time. Here, we present a pump-multiprobe single-crystal X-ray diffraction (SCXRD) technique for studying photoexcited solid-state species with millisecond-to-minute lifetimes. We excite using pulsed LEDs and synchronise to a gated X-ray detector to collect 3D structures with sub-second time resolution while maximising photo-conversion and minimising beam damage. Our implementation provides complete control of the pump-multiprobe sequencing and can access a range of timescales using the same setup. Using LEDs allows variation of the intensity and pulse width and ensures uniform illumination of the crystal, spreading the energy load in time and space. We demonstrate our method by studying the variable-temperature kinetics of photo-activated linkage isomerism in [Pd(Bu_4_dien)(NO_2_)][BPh_4_] single-crystals. We further show that our method extends to following indicative Bragg reflections with a continuous readout Timepix3 detector chip. Our approach is applicable to a range of physical and biological processes that occur on millisecond and slower timescales, which cannot be studied using existing techniques.

## Abstract (web summary)

Time-resolved single-crystal X-ray diffraction experiments largely focus on timescales shorter than microseconds, whereby slower population dynamics are missed. Here, the authors resolve the 3D structures of photoexcited solid-state species with millisecond-to-minute lifetimes using pump-multiprobe SCXRD.

## Introduction

The ability to monitor solid-state reactions in real-time contributes enormously to our understanding of structure-function relationships and assists in designing new materials. The ability to watch chemical and biological processes in 3D, and as they occur, opens up new vistas of chemical research.

SCXRD provides complete 3D structures and is the “gold standard” for studying solid-state processes in single-crystals^1^. Photocrystallography experiments, where molecules in a crystal are activated by light and the structural changes observed using diffraction, have been successfully used to study various light-induced reactions^2–4^. However, diffraction datasets are inherently time-averaged and initial photocrystallographic experiments were limited to timescales of minutes to hours and hence to starting materials or photo-products. Over the last two decades, the advent of fast pulsed lasers, reliable cryogenics, bright synchrotron sources and, most recently, X-ray free-electron lasers (XFELs), have made it possible to study transient species with lifetimes from milliseconds to femtoseconds that have the potential to act as fast molecular switches in opto-responsive materials^5,6^.

The state-of-the-art for time-resolved measurements on macromolecules is Laue diffraction, with synchrotron sources allowing characterization of species with lifetimes down to ~1 ps using streak cameras and bunch-slicing^7,8^, and to femtoseconds at XFELs^5,9,10^. However, solving structures from Laue diffraction is challenging and typically requires a good starting model, making it difficult to identify unknown intermediates. Excepting a handful of examples using Laue methods to study species with microsecond lifetimes^11–13^, molecular crystallography has therefore continued to use monochromatic X-rays despite flux limitations, either at synchrotrons^14–18^, or more recently using some in-house time-resolved set-ups^19,20^.

Pump-probe photocrystallography experiments are also constrained by the excitation source. Experiments typically synchronise the X-ray beam or a gated detector to a pulsed laser with nanosecond to femtosecond pulse-widths. The timing sequence is then dictated by the laser repetition rate, and high-flux X-rays are required to collect measurable diffraction data between pulses. Short, high-intensity laser pulses can lead to photo-bleaching, low photoexcitation levels, sample heating and uneven illumination. Exposure to high-intensity light and/or X-rays can cause rapid crystal damage^21^, while high-flux X-rays can also induce excitation^22,23^.

Previous time-resolved SCXRD experiments have largely focussed on microsecond and shorter lifetimes, due to the availability of lasers operating on these timescales and the pursuit of ever-faster time-resolution. Consequently, there are comparatively few methods suited to lifetimes of minutes to milliseconds. This is despite the many interesting physical and biological transformations occurring on these timescales, including protein folding, ligand binding, phase transitions, crystal nucleation and X-ray-induced crystal damage^6,21,24–27^. Some studies have investigated millisecond kinetics using gated X-ray detectors to follow selected Bragg reflections^19^, but this is insufficient to solve 3D structures and therefore cannot unambiguously identify unknown intermediates.

The latest developments in synchrotron sources and gated photon-counting detectors provide an opportunity to address the limitations of existing time-resolved SCXRD methods and obtain complete datasets with sub-second time-resolution. We have developed a unique LED-pump-X-ray-multiprobe technique for studying single-crystal photoswitching at minute to millisecond timescales. Our method enables collection of high-quality SCXRD datasets considerably faster than previous pump-probe methods^15,16^, while using a pulsed LED pump ensures uniform sample illumination and allows the pump-multiprobe timing sequence and the excitation pulse-width and intensity to be straightforwardly tuned to maximise photoexcitation and minimise crystal damage. This delivers higher excited-state (ES) populations than in traditional laser experiments, enabling the 3D structure of unknown species to be solved without an initial model. We have applied our technique to study the photo-activated NO_2_$\to$ONO linkage isomerism in [Pd(Bu_4_dien)(NO_2_)][BPh_4_] (**1**, Bu_4_dien = *N,N,Nʹ,Nʹ*-tetrabutyldiethylenetriamine, BPh_4_ = tetraphenylborate)^6^. We obtain original insight into the photoexcitation at close-to-ambient temperature and visualise changes in electron-density on irradiation as a time-resolved “molecular movie” with 400 ms resolution.

This methodology can be adapted to study a variety of molecular/macromolecular processes and may be paired with techniques such as HATRX to improve the signal-to-noise ratio and/or access even faster timescales^28^.

## Results

### Model system

Our method was developed against the model linkage isomer crystal **1**. **1** photoexcites from a ground-state (GS) nitro-(*η*^1^-NO_2_) to an ES *endo*-nitrito-(*η*^1^-ONO) isomer with 100% conversion. Full details of the GS and photoinduced ES structures are discussed in detail previously^29^, and a summary of key data is included in the Supplementary Information, Supplementary Note 1, Fig. S1.

1. The reverse ONO→NO_2_ process is thermally induced and the ES lifetime is strongly temperature-dependent^30^. This makes **1** an ideal test system for time-resolved SCXRD methods, as the ES lifetime can be straightforwardly matched to the measurement timescale by selecting an appropriate temperature. Preliminary experiments to confirm the response of the crystal in the experiment set-up were performed as previously described^30^.

### Pump-multiprobe data collection strategy

**Fig. 1:**
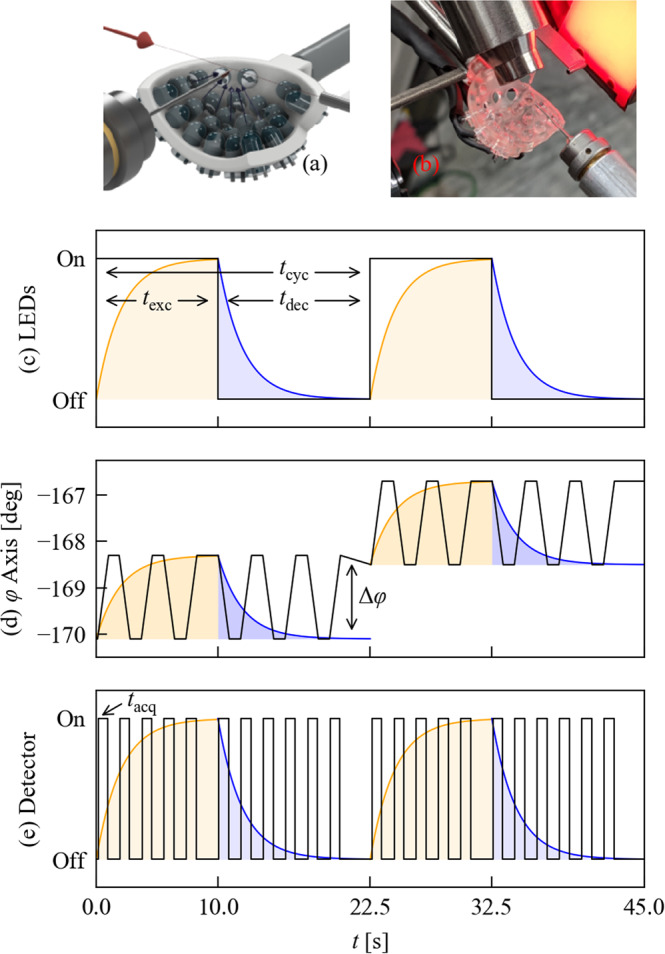
Pump-probe SCXRD experiments using pulsed LEDs. **a**, **b** A CAD drawing (**a**) and photograph (**b**) of the 3D-printed LED holder in situ on the diffractometer. **c**, **d** Illustrate the data-collection procedure. The sample is subjected to repeated excitation/decay cycles of duration ${t}_{{{{{{\rm{cyc}}}}}}}$ (**c**). The LEDs are activated for ${t}_{{{{{{\rm{exc}}}}}}}$ to generate a photo-stationary excited-state (ES) population (yellow) and then switched off for ${t}_{{{{{{\rm{dec}}}}}}}$ to allow the ES population to decay (blue). During each sequence the goniometer ${{{{{\rm{\varphi }}}}}}$-axis is swept backward and forward through an angle range $\triangle {{{{{\rm{\varphi }}}}}}$ (**d**) and the detector is electronically gated to record a series of data frames over a time interval ${t}_{{{{{{\rm{acq}}}}}}}$ (**e**). The start and end points of the ${{{{{\rm{\varphi }}}}}}$ sweep are incremented after each sequence, thereby obtaining a time series of complete ${{{{{\rm{\varphi }}}}}}$ scans sampling the excitation/decay curve with a resolution ${t}_{{{{{{\rm{acq}}}}}}}$. The process is repeated to collect ${{{{{\rm{\varphi }}}}}}$ scans with the other goniometer axes in different positions to build up a full single-crystal dataset.

The light-pump used was a bespoke pulsed LED array (Fig. 1a, b). A typical pump-probe sequence with duration ${t}_{{{{{{\rm{cyc}}}}}}}={t}_{{{{{{\rm{exc}}}}}}}+{t}_{{{{{{\rm{dec}}}}}}}$, where the pump is on for an excitation time ${t}_{{{{{{\rm{exc}}}}}}}$ and off for a decay time ${t}_{{{{{{\rm{dec}}}}}}}$, is shown in Fig. 1c. A major challenge in time-resolved SCXRD experiments is designing a data-collection strategy that provides complete X-ray datasets, allowing for full structural determination, within a reasonable overall experiment time. To obtain accurate intensities each reflection needs to be sampled multiple times across its profile, which is usually achieved by rotating the crystal around one axis (here ${{{{{\rm{\varphi }}}}}}$) while collecting data over a sequence of angle ranges ${{{{{\rm{\varphi }}}}}}$ $\to$ ${{{{{\rm{\varphi }}}}}}+\triangle {{{{{\rm{\varphi }}}}}}$ as individual diffraction images. In traditional pump-probe experiments, each measurement is taken at a centre-time $t$ relative to the start of the cycle, and the entire experiment is repeated for several $t$ over the complete ${t}_{{{{{{\rm{cyc}}}}}}}$. With delays of milliseconds and longer, a full time-resolved experiment collecting sufficient quality data for several $t$ easily requires several hours, which is especially problematic for crystals that degrade with repeated light and/or X-ray exposure.

To address this, we implemented a “pump-multiprobe” strategy where data for all required $t$ are obtained in one collection (Fig. 1d, e). During each cycle the crystal is rotated forwards and backwards multiple times through the same $\triangle \varphi$, sampling the same angular range at multiple $t$, and an electronically gated Pilatus 300 K detector is used to collect each $\triangle \varphi$-sweep as a separate diffraction image. The acquisition time ${t}_{{{{{{\rm{acq}}}}}}}$ for each image is determined by the gate length and diffractometer rotation speed and dictates the time-resolution, here defined as the time over which each 3D structure is averaged. During each subsequent ${t}_{{{{{{\rm{cyc}}}}}}}$ the crystal is moved through the next angular range and data again collected for all $t$, and the process repeated to build-up a 180° $\varphi$-scan. This multi-oscillation approach reduces the time needed to collect a complete set of time-resolved datasets to <120 min, far more time-efficient than a conventional pump-probe experiment.

We also implemented automated, on-the-fly data-processing to enable rapid analysis. The diffraction images are sorted by $t$ as they are recorded and, once all experiments are complete, datasets are indexed, integrated and solved automatically. A full sequence of 3D structure snapshots are thus obtained over the cycle, each averaged over ${t}_{{{{{{\rm{acq}}}}}}}$. Once an initial model for the structures is obtained, this can be included in the processing pipeline so that the data is automatically refined to extract the ES populations from each snapshot.

### Experiment design

The pump-multiprobe strategy was used to examine the time-dependent ES population in **1** over a range of timescales. The experimental conditions required to match the ES lifetime to the intended measurement timescales were determined using numerical simulations^30^ parameterised with excitation and decay kinetic measurements, and ES populations under continuous irradiation, all measured using conventional SCXRD (Supplementary Information, Supplementary Note 4, Tables S1–S15, Figs. S8–S16). These simulations allowed us to select a ${t}_{{{{{{\rm{cyc}}}}}}}$ and optimise the ${t}_{{{{{{\rm{exc}}}}}}}$, ${t}_{{{{{{\rm{dec}}}}}}}$ and measurement temperature to maximise the ES/GS ratio, enabling precise, targeted design of the pump-multiprobe experiments. We selected five ${t}_{{{{{{\rm{cyc}}}}}}}$ from 14 to 170 s with ${t}_{{{{{{\rm{acq}}}}}}}$ from 400 ms to 8 s (Table 1). The simulations suggested a temperature range of 260–290 K, which is above the 255 K ceiling of our previous decay kinetics measurements using conventional SCXRD methods^30^.

**Table 1:** Pump-probe sequences tested in this work.

${t}_{{{{{{\rm{cyc}}}}}}}$ [s]	${t}_{{{{{{\rm{exc}}}}}}}$ [s]	${t}_{{{{{{\rm{dec}}}}}}}$ [s]	$\triangle {{{{{\rm{\varphi }}}}}}$ [deg]	${t}_{{{{{{\rm{acq}}}}}}}$ [s]
170	55	115	1.6	8.0
108	35	73	8.0	4.0
35	14	21	3.2	1.6
22	8	14	1.6	0.8
14	5	9	0.8	0.4

Each row lists the key parameters shown in Fig. 1, viz. the cycle time ${t}_{{{{{{\rm{cyc}}}}}}}$, the excitation and decay times ${t}_{{{{{{\rm{dec}}}}}}}$/${t}_{{{{{{\rm{exc}}}}}}}$, the ${{{{{\rm{\varphi }}}}}}$-angle range $\triangle {{{{{\rm{\varphi }}}}}}$ the crystal is rotated through during each cycle, and the time-resolution ${t}_{{{{{{\rm{acq}}}}}}}$ of the measured X-ray structures.

### ES population dynamics on sub-second timescales

A total of 12 experiments were performed over the five ${t}_{{{{{{\rm{cyc}}}}}}}$, each using a new crystal. By optimising the sequence timings and measurement temperatures, we obtained maximum ES conversions ${\alpha }_{{{\max }}}$ of 33.7% (${t}_{{{{{{\rm{acq}}}}}}}$ = 8 s), 21.3% (${t}_{{{{{{\rm{acq}}}}}}}$ = 4 s), 10.7% (${t}_{{{{{{\rm{acq}}}}}}}$ = 1.6 s), 11.8% (${t}_{{{{{{\rm{acq}}}}}}}$ = 800 ms), and 10.4% (${t}_{{{{{{\rm{acq}}}}}}}$ = 400 ms). Full ${\alpha }_{{{\max }}}$ data for all 12 experiments are given in Supplementary Information, Supplementary Note 4, Supplementary Table S16. After fitting, the refined populations were found to be within 68–98% (mean 84%) of the predicted maximum steady-state ES population ${\alpha }_{{{{{{\rm{SS}}}}}}}$ achievable under continuous illumination at each experiment temperature (Supplementary Information, Supplementary Note 6, Supplementary Tables S18 and S19). This indicates that we obtain photoconversion levels approaching the theoretical maximum in most crystals.

The ES conversion is influenced by a number of factors including crystal size and shape^30^, and we thus observed some variation in the ES fractions obtained under similar conditions. In all cases, however, the ${\alpha }_{{{\max }}}$ obtained with our LED set-up are a significant improvement on the few % reported for traditional laser-pump-X-ray-probe studies^14–17,19^.

Each LED-pump-X-ray-multiprobe experiment yields a series of 3D structures spanning the excitation and decay periods. Good-quality SCXRD data and structure refinements were obtained from all experiments, with residual factors of *R*_int_ = 9.16–9.85% (mean 9.55%) and *R*_1_ = 6.24–6.41% (mean 6.32%) across the nine datasets collected at the shortest ${t}_{{{{{{\rm{acq}}}}}}}$. Supplementary Information, Supplementary Note 5, Supplementary Table S17 provides full data for selected structures across all 12 pump-multiprobe experiments.

**Fig. 2:**
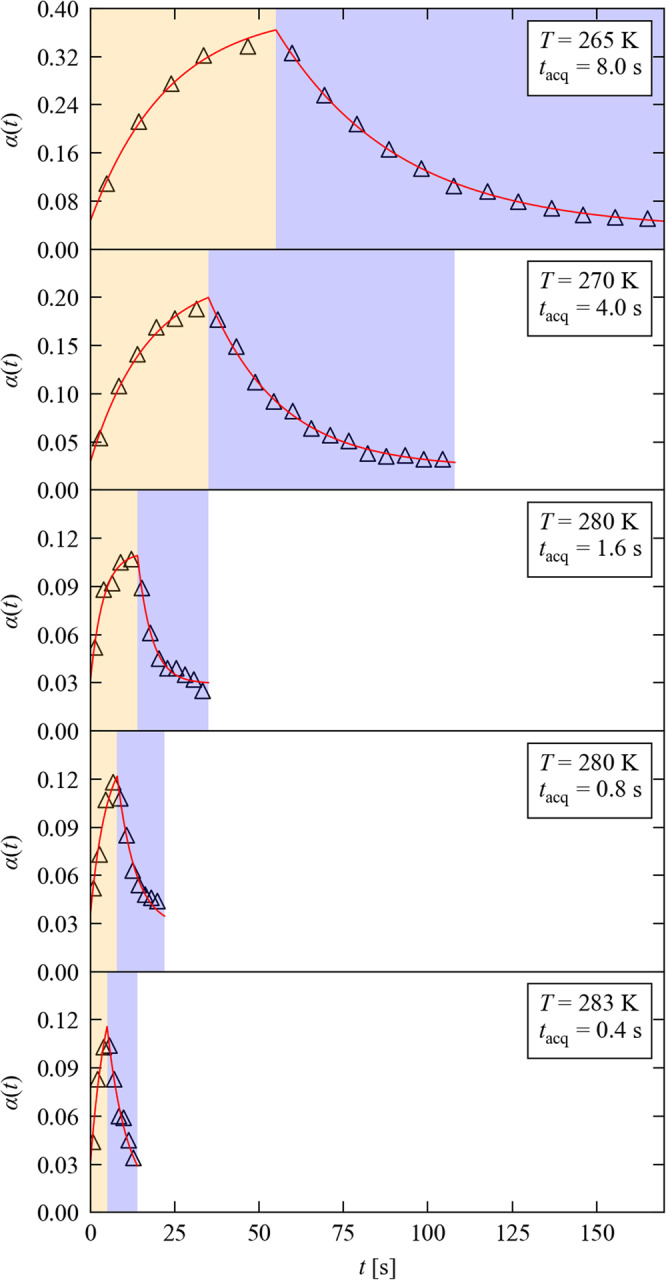
Time-resolved pump-multiprobe single-crystal diffraction measurements on 1. The five plots show the time dependence of the ES population $\alpha (t)$ during successively shorter pump-multiprobe sequences where the temperature and excitation time are optimised to engineer similar kinetic behaviour. On each plot, the orange and blue shaded regions mark the excitation and decay parts of the sequence, respectively. The markers show the measured $\alpha (t)$, each refined from a full single-crystal X-ray data set averaged over the time ${t}_{{{{{{\rm{acq}}}}}}}$ indicated on each plot. The solid lines are fits of the data using numerical simulations with a two-process JMAK model with competing excitation and decay.

The fractional occupation of GS and ES isomers for each structure are refined using a standard disorder model, allowing the evolution of the ES population $\alpha (t)$ to be followed. We define the conversion fraction $\triangle \alpha \left(t\right)=\alpha \left(t\right)-\alpha \left(t=0\right)$ as the percentage of molecules that are converted to the ES isomer at a time $t$. Figure 2 shows the behaviour in experiments with increasingly faster ${t}_{{{{{{\rm{cyc}}}}}}}$ and shorter ${t}_{{{{{{\rm{acq}}}}}}}$, where in each case the temperature was optimised to ensure near-complete ES decay. The same behaviour is seen in all experiments, suggesting the same fundamental isomerisation mechanisms at all the timescales/temperatures studied.

**Fig. 3:**
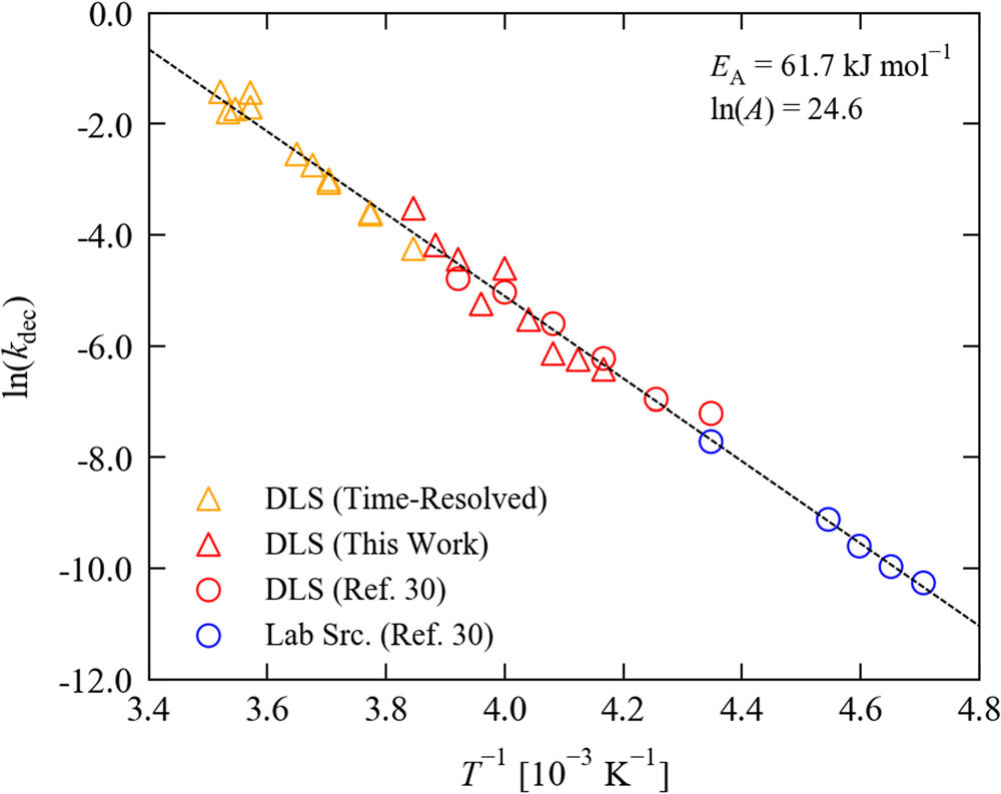
Temperature dependence of the decay rate of the metastable isomer of 1. The plot shows an Arrhenius analysis of decay rate constants *k* obtained from three sets of measurements, *viz*. low- and mid-temperature photocrystallographic decay measurements, performed using a lab source (blue) and at the Diamond Light Source synchrotron facility (DLS - red), and high-temperature time-resolved measurements performed with our pump-multiprobe strategy (yellow). The black line is a fit to the Arrhenius law with the fitting parameters as shown. The low-temperature and some of the mid-temperature data are taken from our previous work^30^.

To investigate this further, we assume competing, independent excitation and decay processes, each described by the Johnson-Mehl-Avrami-Kolmogorov model^30^, and use numerical simulations to fit excitation and decay rates. Figure 2 confirms this model reproduces the measured data well, providing confidence in the fitted parameters. Fitted parameters for all pump-multiprobe experiments are given in Supplementary Information, Supplementary Note 6, Supplementary Figs. S17–S28, Supplementary Table S18. At low temperature, the decay rate follows the Arrhenius law^30^. Figure 3 shows an Arrhenius analysis including low- and mid-temperature rate constants obtained from conventional SCXRD measurements, and high-temperature rate constants from the pump-multiprobe experiments. Both datasets fit the same trendline, providing strong evidence that the decay follows the same mechanism over a wide temperature range. We extract an activation energy ${E}_{{{{{{\rm{A}}}}}}}$ of 61.7 kJ mol^−1^ and an attempt frequency $A$ of 48.3 GHz.

Finally, we note that we do not observe complete ES decay during the experiments. Since the decay is exponential, we adjusted ${t}_{{{{{{\rm{dec}}}}}}}$ to 4–5$\times$ the predicted kinetic ${t}_{1/2}$ at the measurement temperature to balance complete decay to a “clean” GS against a reasonable ${t}_{{{{{{\rm{cyc}}}}}}}$. Despite this, we observed a low-level background excitation $\alpha \left(t=0\right)$ of ~4%, which we ascribe to a small but measurable level of X-ray excitation. While the ${\alpha }_{{{\max }}}$ levels we measure have sufficient range to follow the excitation behaviour clearly, this finding has implications for the low excitation levels observed in traditional laser-pump-probe experiments.

### Evolution of the electron density: molecular movies

**Fig. 4:**
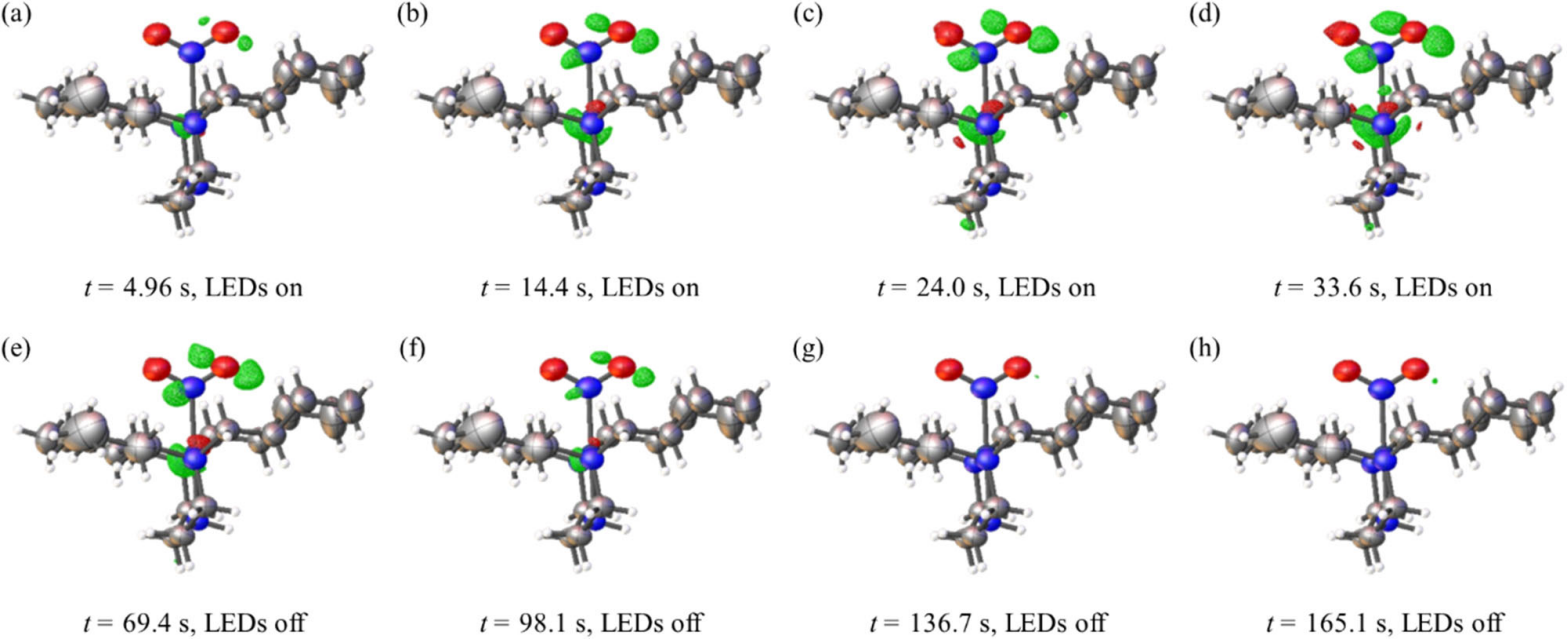
Selected photo-difference maps illustrating the change in electron density in 1 during photoexcitation and decay at 265 K. Fourier electron-density difference maps calculated between the fixed GS coordinates and the ES structure data (ellipsoids shown at 50% probability). Green and red contours show regions of density accumulation and depletion, respectively. **a**–**d** are from structures recorded during the LED illumination period ${t}_{{{{{{\rm{exc}}}}}}}$, while **e**–**h** are from structures recorded during the ES decay period ${t}_{{{{{{\rm{dec}}}}}}}$. All photo-difference maps are set to a consistent level of ± 0.8 e A^−3^ per contour for comparable images, with maximum and minimum electron density levels in each image as follows (in e A^−3^): **a** min = −0.901, max = 1.729; **b** min = −2.023, max = 2.864; **c** min = −2.511, max = 4.087; **d** min = −2.838, max = 5.249; **e** min = −2.230, max = 3.657; **f** min = −0.975, max = 1.682; **g** min = −0.670, max = 0.783; **h** min = −0.666, max = 1.095.

The information provided by the pump-multiprobe measurements also allows us to visualise the changes in electron density on photoexcitation. This is achieved by generating Fourier electron-density difference maps (photo-difference maps) between the GS structure model at $t=0$ and ES data recorded during the pump-multiprobe cycle. Figure 4 shows a series of photo-difference maps generated from an experiment at 265 K with ${t}_{{{{{{\rm{cyc}}}}}}}$ = 170 s, ${t}_{{{{{{\rm{exc}}}}}}}$ = 55 s, ${t}_{{{{{{\rm{dec}}}}}}}$ = 115 s, ${t}_{{{{{{\rm{acq}}}}}}}$ = 8 s and maximum $\triangle \alpha (t)$ = 33.7%. Positive/negative residual peaks indicate regions where electron-density accumulates/depletes on excitation, showing the movement of atoms during the isomerisation.

The time-resolved snapshots can be combined to create a molecular movie showing the nitro$\leftrightarrow$nitrito conversion (Supplementary Information, Supplementary Note 7 and Supplementary Movies 1–5). We find that shorter time-resolutions decrease the quality of the photo-difference maps due to spurious ripples in the difference density, which we attribute to the reduced diffraction intensity due to X-ray flux limitations at shorter ${t}_{{{{{{\rm{acq}}}}}}}$ (although we emphasise that this data is still sufficient for good-quality structure solution).

### Future developments: improved time-resolution with continuous readout using the Timepix3 detector chip

The practical limit on the number of time-delays and the ${t}_{{{{{{\rm{acq}}}}}}}$ in our pump-multiprobe experiment are determined by three variables: (1) the X-ray flux; (2) the “dead-time” needed to move the diffractometer between ${t}_{{{{{{\rm{acq}}}}}}}$; and (3) the detector read-out time. At 400 ms we only require 5% of the available beam, so modern synchrotron sources are easily capable of higher time-resolution. The dead-time for diffractometer movement can be minimised through alternative data-collection strategies. However, the 3 ms read-out of the Pilatus 300 K becomes limiting at ~10 ms and faster. This can be addressed using purpose-built time-resolved detectors such as those based on the Timepix3 hybrid active-pixel detector chip^31^. The Timepix3 detects and timestamps X-ray photons with an accuracy of 25 ns and outputs a continuous data-stream containing the 2D position, time of arrival (ToA) and time over threshold (ToT) of each photon event. This continuous data-stream eliminates read-out time and allows ${t}_{{{{{{\rm{acq}}}}}}}$to be adjusted after collection, meaning that the experiment need not be constrained by having to choose a target time-resolution before data-collection.

To demonstrate the potential of our method to harness this technology, we performed proof-of-concept experiments using a single-module Timepix3 detector being developed at Diamond Light Source. The 1.98 cm^2^ detector area was sufficient only to follow a single reflection, so we used our pump-multiprobe datasets to identify the (−2 1 0) reflection as tracking the ES population. We then ran a series of pump-probe experiments using similar timing sequences to the full SCXRD measurements, while recording continuously with the Timepix3 chip.

**Fig. 5:**
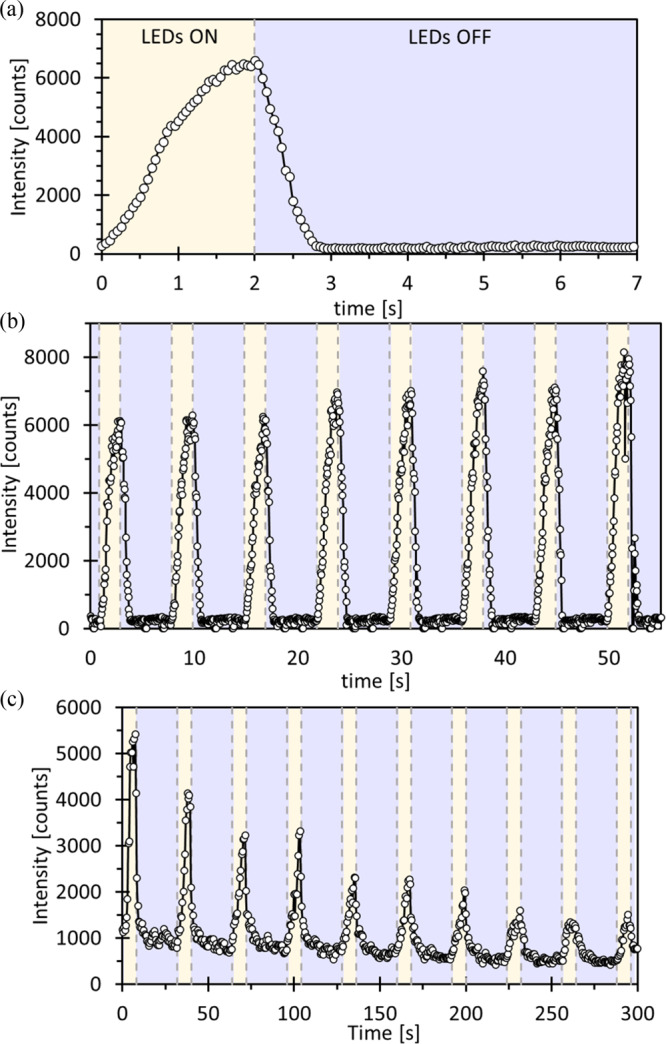
Intensity of the (−2 1 0) reflection of 1 recorded using a Timepix3 detector chip. **a** Intensity change during one cycle with ${t}_{{{{{{\rm{exc}}}}}}}$ = 2 s (LEDs on) and ${t}_{{{{{{\rm{dec}}}}}}}$ = 5 s (LEDs off) at $T$ = 286 K, averaged over eight repeats. **b** Intensity change over ten consecutive cycles with ${t}_{{{{{{\rm{exc}}}}}}}$ = 2 s and ${t}_{{{{{{\rm{dec}}}}}}}$ = 5 s at $T$ = 286 K. **c** Intensity change over ten consecutive cycles with ${t}_{{{{{{\rm{exc}}}}}}}$ = 8 s and ${t}_{{{{{{\rm{dec}}}}}}}$ = 24 s at $T$ = 280 K. In each case the photon events recorded by the detector are integrated into 50 ms time bins.

Figure 5a shows the intensity changes over a representative pump-multiprobe cycle. These are an excellent qualitative match to the refined ES population behaviour from full SCXRD experiments. Figure 5b, c shows the results of repeated pump-multiprobe cycles and show that, while at shorter ${t}_{{{{{{\rm{exc}}}}}}}$ the maximum intensity is relatively consistent between cycles, for longer ${t}_{{{{{{\rm{exc}}}}}}}$ the effects of the photobleaching are clearly evident. Qualitative information on NO_2_$\leftrightarrow$ONO switching can therefore be quickly determined using this method. However, we found that data-fitting did not yield quantitative kinetic information comparable to the full SCXRD experiments, which highlights the importance of our method in providing unambiguous 3D structure information at all time-points. The continuing development of larger-area Tristan 1 M/10 M Timepix3 detectors at Diamond will soon enable collection of complete SCXRD data, and the flexibility of not requiring a fixed ${t}_{{{{{{\rm{acq}}}}}}}$ may allow our method to access finer time-resolution.

## Discussion

The pump-multiprobe methodology presented here leverages the advances in photon-counting detectors to study solid-state processes at second-to-millisecond timescales, with numerous benefits over existing pump-probe methods used for faster processes. Our method yields a complete set of high-quality SCXRD datasets averaged over sub-second timescales, allowing the 3D structures of light-activated species to be determined without prior knowledge. By taking full advantage of the slower timescales, our method represents a powerful technique for following the population dynamics of ES species to obtain kinetic and mechanistic information, and can be utilised to unambiguously identify short-lived intermediate species by visualising changes in the electron density through creating photo-difference maps.

For model complex **1**, at the temperatures investigated (260 to 284 K) and the timescales that we access, we observe only the known *endo*-nitrito-(*η*^1^-ONO) isomer and no evidence of other transient linkage isomers. The system can thus be regarded as a simple binary switch that is OFF in the ground-state (GS) nitro-(*η*^1^-NO_2_) form and ON in the *endo*-nitrito-(*η*^1^-ONO) form and therefore has the potential to act as a ‘near room temperature’ molecular switch for opto-electronic applications. However, in other, related materials^32,33^, transient *exo*-nitrito-(*η*^1^-ONO) isomers have been observed, but always at cryogenic temperatures far below those studied here. Given the strong temperature-dependence of nitrite isomer lifetimes^30^, it is important to establish whether an *exo*-nitrito form might exist at potential operational temperatures and interfere with the simple binary switching. From our experimental observations in the present study it is likely that the *exo*-nitrito lifetime is much shorter than milliseconds near ambient temperature, explaining why it is not observed in this study. To confirm the existence of the *exo*-nitrito isomer as a possible transient intermediate in the nitro-(*η*^1^-NO_2_) to endo-nitrito(*η*^1^-ONO) pathway, the Nudged Elastic Band (NEB) method was used to calculate the minimum energy pathway between the two known states (Supplementary Information, Supplementary Note 10, Supplementary Movie 6 and Supplementary Figs. S32, S33 and S34)). A local minimum is found with an *exo*-nitrito geometry; however, the minima is shallow with only 6.6 kJ mol^−1^ required to continue to the endo-nitrito(*η*^1^-ONO) compared to the expected activation energy of 114 kJ mol^−1^ (Supplementary Information, Supplementary Note 10, Supplementary Figs. S32, S34) for the whole process. This indicates that this state is unlikely to be long-lived and so will not interfere with the simple binary switching of the isomers on the millisecond timescale near room temperature. This result compares well to the prior photocrystallographic experiments^32,33^. However, the NEB output does show that a considerable amount of movement is expected at the Pd(II) centre to accommodate the rearrangement of the nitrite ligand. This is interesting and compares well to the changes in electron density seen at the Pd(II) centre in the second- and millisecond-resolved molecular movies (Fig. 4, Supplementary Information, Supplementary Note 7, Supplementary Movies 1–5).

The pump-multiprobe method records data across multiple time-delays in each pump-probe cycle, which substantially shortens the experiment compared to previously developed approaches and ensures that measurements at different time-delays are not unequally affected by cumulative crystal damage, photobleaching and/or X-ray excitation. A comparison of the Wilson plots for all the pump-multiprobe datasets confirms there is no significant deterioration of the crystal during the process, with all plots showing a strong linear fit and values of the temperature factor B constant (within error) over all time points measured at a particular temperature. Example Wilson plots for the lowest (260 K) and highest temperature (284 K) experiments are included in Supplementary Information, Supplementary Note 8, Supplementary Figs. S29–S30 and Supplementary Data files 5 and 6. The shorter overall experiment time certainly helps to minimise these issues and, where they do occur, the experiment design means that all time-delays are affected equally and thus remain comparable.

The use of a pulsed-LED pump, rather than a laser, also sets our method apart from existing pump-probe experiments. Provided an LED pump can deliver sufficient power, it can be built with low-cost components to provide a variety of wavelength/power profiles and allows complete control over the timing sequence and illumination direction, while coverage can be optimised without complex alignment, optics and safety protocols. An LED pump also spreads the irradiation energy load in time, minimising unwanted effects such as heating and potentially yielding higher overall photoexcitation in crystals like **1**, where the excitation and decay are in equilibrium. We confirmed that the level of crystal heating from the LED sphere is low by determining the temperature scale factor ${k}_{B}$ from Photo-Wilson plots created for all the pump-multiprobe experiments, following the method outlined by Coppens et al.^34^. Full details of this analysis is provided in the Supplementary Information, Supplementary Note 9, Supplementary Fig. S31. The plots show an average ${k}_{B}$ value of 1.069 ± 0.028 across all 12 datasets that, when compared to the literature, indicates a negligible temperature rise in the crystal^34^. The flexibility of this setup also allows the same experiment to study a range of light-induced phenomena at timescales from minutes to microseconds, depending on the LED components used. On the other hand, designing, commissioning, and maintaining a laser setup with similar flexibility would be very resource intensive. We note that laser diodes capable of pulses down to a few hundred picoseconds are also available^35^.

In linkage isomers like **1**, the maximum achievable photoexcitation at a given temperature will always be limited by the competing thermal decay to the steady-state occupation achieved under continuous illumination^30^. While a short burst of high-intensity photons might produce a non-equilibrium population for a short period of time, the low conversion fractions typically seen following laser excitation suggests this does not happen efficiently in practice. Indeed, our previous work with **1** showed that the conversion saturates beyond a certain brightness, indicating a threshold beyond which excess photons are either not absorbed^30^, or, worse, converted to heat to elevate the decay rate. Even using the LED pump, we observed gradual bleaching during our experiments and this would likely be a larger issue with lasers delivering comparable or higher powers over much shorter pulse-widths. There is a clear need for more systematic investigation of these problems and their implications for pump-probe experiments.

Finally, the observation of spurious peaks in the photo-difference maps at shorter ${t}_{{{{{{\rm{acq}}}}}}}$ highlight that the experiment remains diffraction-limited and the signal-to-noise ratio needs to be improved for faster timescales. For **1**, this is not a limitation of the X-ray source but is necessary to avoid X-ray-induced excitation at higher flux. This is likely to be a problem for many crystal systems and motivates the development of faster and more sensitive detector technologies. In this vein, the flexibility afforded by moving away from traditional imaging to event-counting detectors, such as the Timepix3 chip explored in this work, will be crucial for future time-resolved SCXRD studies at shorter timescales.

## Methods

### Test crystal

**1** is a previously reported solid-state linkage isomer system and crystallises as a tetrahydrofuran solvate in the monoclinic space group *P*2_1_/*n* with one cation, one BPh_4_ counter-anion and one THF molecule per asymmetric unit (Supplementary Information, Fig. S1). The photoactive [Pd(Bu_4_dien)(NO_2_)]^+^ cation excites from a ground-state (GS) nitro (*η*^1^-NO_2_) to an excited-state (ES) *endo*-nitrito (*η*^1^-ONO) isomer in the single-crystal with 100% conversion by irradiating at λ ≈ 400 nm^29^. The reverse ONO → NO_2_ process is thermally activated with a measured activation energy ${E}_{{{{{{\rm{A}}}}}}}$ of 51.6–60.3 kJ mol^−1 ^^30^. Complete GS → ES excitation has a minimal effect on the surrounding crystal structure, with a small 0.14% expansion of the lattice at 100 K and negligible loss of diffraction over repeated transformations^29^. Crucially for the present work, the ES lifetime is strongly temperature-dependent, with a continuous variation in the kinetic half-life over seven orders of magnitude from ~10^7 ^s (115 days) at 180 K and 1 s at room temperature^30^. Full crystal data and images for **1** are provided in the Supplementary Information, Supplementary Note 1 and Supplementary Fig. S1.

### Excitation source

The light pump in our experiments is a pulsed LED array incorporating 18 round 3 mm LEDs (Bivar UV3TZ-400-30) mounted into a part-spherical holder to point directly at the crystal (Fig. 1a). This custom 3D-printed LED sphere can hold up to 25 round (T-1) 3 mm through-hole LEDs, with all diodes pointing directly at the sample position. Full details of the electronic set-up of the sphere are included in the Supplementary Information, Supplementary Note 2 and Supplementary Figs. S2 and S3.

The array is mounted directly below the sample with a crystal-to-LED distance of 9.57 cm (Fig. 1b) and illuminates the crystal without impeding the X-ray beam path or restricting the detector position at any point during the experiment. Rotation of the crystal about $\varphi$ during the LED pulses further ensures uniform illumination. The light power at the crystal was measured to be 23 mW (Supplementary Information, Supplementary Note 2(ii)). The LED rise and fall times were tested and found to be negligible on the timescale of the experiment, and pulse separation down to ~200 μs could be achieved (Supplementary Information, Supplementary Note 2 (iii)). Reliable pulse trains with microsecond and shorter repetition rates could likely be obtained using alternative LEDs or laser diodes. Full details of the timing synchronisation between the LEDs and the diffractometer hardware are provided in the Supplementary Information.

### Sample excitation tests

We first carried out test experiments to determine whether prolonged exposure to LED light and/or synchrotron X-rays could lead to unfavourable changes in the sample, e.g. crystal degradation or X-ray induced excitation. Full details are provided in the Supplementary Information, Supplementary Note 3, Supplementary Figs. S4–S7. Though no appreciable crystal degradation was observed in the X-ray tests, a steady increase in the ES nitrito-ONO isomer occupancy was measured with increasing exposure time. This X-ray induced excitation could be limited by increasing the attenuation of the synchrotron beam, leading us to select a 5% beam intensity for experiments, which produced a baseline X-ray excitation of *c.a*. 3% in testing. LED exposure tests revealed that prolonged irradiation for *c.a*. 4 h led to irreversible damage at the crystal surface, causing a visible colour change from pale-yellow to orange, alongside gradual photobleaching with time (Supplementary Information, Supplementary Note 3, Supplementary Fig. S7). To minimise the influence of these effects, a new crystal was used for each experiment.

### Pump-multiprobe data collection

Data were collected using an XPS four-circle diffractometer equipped with a Pilatus 300 K detector and an Oxford Cryosystems Cryostream 7 liquid-nitrogen cooling device for temperature control. The GDA software^34^, which functions as the user interface for setting up data collections on beamline I19 at Diamond Light Source, was used to implement the pump-multiprobe synchronisation, further details of which are provided in the Supplementary Information, Supplementary Methods. The pump-multiprobe data collection strategy was carefully designed to maximise coverage of reciprocal space whilst minimising the “downtime” due to diffractometer movements between data collection positions. Full details of the strategy determination process, including representative examples, are provided in the Supplementary Information (Supplementary Information, Supplementary Methods).

### Data-processing

Data were processed through a custom-designed automated data processing pipeline developed using the beamline systems^35–40^. Full details of the automated process are provided in the Supplementary Information (Section 11(iii)). All single-crystal X-ray diffraction data indexing and integration are performed by DIALS^39^, while data scaling and absorption correction are applied by AIMLESS^40^, all of which are run through the Xia2 interface. Structure solutions are performed using SHELXT^41^, and all structures are refined by full matrix least squares on *F*^2^ with SHELXL^42^. Hydrogen atoms were positioned geometrically and refined using a riding model. The hydrogen atom isotropic displacement parameters were fixed to *U*_iso_(H) = 1.5 $\times$ (for CH_3_) or *U*_iso_(H) = 1.2 $\times$ (for CH_2_ and CH). ES population conversion fractions $\alpha (t)$ for all structures were refined directly from the data using a standard disorder model for the partially isomerised nitrite ligand, utilising SHELX PART instructions. Photodifference maps were generated between the GS model and ES diffraction data, with full details for this process recorded in the Supplementary Information, (Section 11(iv)).

### Kinetic modelling and data fitting

Fitting of the GS $\to$ ES excitation and reverse ES $\to$ GS decay processes was achieved using the Johnson-Mehl-Avrami-Kolmogorov (JMAK) model^43–45^, as in previous work^30^. Full details of the fitting procedures are included in the Supplementary Information, Supplementary Note 4(ii)).

We also used our previously developed simulation tool to fit the measured pump-probe data and extract rate constants for the excitation and decay processes (the Avrami exponents ${n}_{{{{{{\rm{exc}}}}}}}$ for both processes were again fixed to unity)^30^. Full details of the pre-experiments, modelling and data fitting are given in the Supplementary Information, Supplementary Notes 4 and 6).

### Timepix3 single-reflection experiments, data processing and data fitting

Proof-of-concept measurements were performed using a single-module Timepix3-based detector to follow the (−2 1 0) reflection, which was found to show a large increase in intensity between the GS and ES structures.

The raw output from the Timepix3 chip is a data stream containing the position, time of arrival (ToA) and time over threshold (ToT) value for each detected photon. This data stream was synchronised to the timing electronics to record additional timestamped events marking the start and end of each pump-probe sequence. After collecting for a fixed time, the photon capture events are rebased to the pump-probe cycle time, a region of interest (RoI) containing the reflection is defined together with a nominal ${t}_{{{{{{\rm{acq}}}}}}}$, which we chose to be 50 ms to provide a suitable number of intensity-sampling points across pump-multiprobe cycle periods of a few seconds in duration, and the intensity (photon counts, number of events) in the RoI is binned into time windows of length ${t}_{{{{{{\rm{acq}}}}}}}$.

A script was used to automatically extract the photon count data from the Timepix3 data stream files and produce histogram plots of the reflection intensity as a function of irradiation and experiment time, as shown in Fig. 5.

### Simulation of complexes

The cationic moieties of **1** in both the 100% nitro-(*η*^1^-NO_2_) and 100 % (*endo*-nitrito-(*η*^1^-ONO) forms were structurally optimised in ORCA 5.0.0 using the crystal data as an input geometry. The PBE0 functional with the RIJCOSX approximation and the def2/J auxiliary basis set were used throughout. Following structural optimisation and frequency calculation with the def2-SV(P) basis sets, further refinement was carried out using the def2-TZVPP basis set. Nudged elastic band (NEB) calculations were carried out with the same methodology^46^, using the NEB-TS method with the optimised nitro-(*η*^1^-NO2) isomer of **1** as the initial point and the optimised *endo*-nitrito-(*η*^1^-ONO) isomer of **1** as the end point.

## Supplementary information

Raithby_PR File

Supplementary Material

Description of Additional Supplementary Files

Supplementary Movie 1

Supplementary Movie 2

Supplementary Movie 3

Supplementary Movie 4

Supplementary Movie 5

Supplementary Movie 6

Supplementary Data 1

Supplementary Data 2

Supplementary Data 3

Supplementary Data 4

Supplementary Data 5

Supplementary Data 6

## Data Availability

Raw data and codes used for data collection, processing and analysis can be obtained from the authors on reasonable request. Crystal data and CIF files for all 178 structures collected are available from the Cambridge Structural Database, deposition numbers: 2127495–2127511, 2127514–2127530, 2127548–2127564, 2127565–2127583, 2127724–2127742, 2127809–2127827, 2127832–2127850, 2128443–2128453, 2128633–2128645, 2128661–2128669, 2128671–2128679, 2132660–2132668. These data can be obtained free of charge from The Cambridge Crystallographic Data Centre via www.ccdc.cam.ac.uk/data_request/cif. Cif files and checkcif files are available in Supplementary Data 1 and 2 respectively. The electronic structure calculations are available in Supplementary Data files 3 and 4, respectively.

## Code availability

Single crystal X-ray data were collected using the open-source Diamond Light Source General Data Acquisition (GDA) software, for which further access information is available at www.opengda.org. Single crystal X-ray data were indexed, integrated and scaled using the open source xia2 software (https://github.com/xia2/xia2), and solved and refined using the SHELXT and SHELXL software (https://shelx.uni-goettingen.de; free for academic and non-for-profit users). The code for performing the kinetic analyses and numerical simulations, as well as the Photo-Wilson plot analysis, is available on GitHub at https://github.com/JMSkelton/Linkage-Isomer-JMAK-Kinetics. This code makes use of the Python 3 programming language (https://www.python.org) with the NumPy and SciPy packages (https://numpy.org, https://scipy.org) as well as our FitLib package (https://github.com/skelton-group/FitLib), all of which are open source and freely available.
