# Supplementary material for: LED-pump-X-ray-multiprobe crystallography for sub-second timescales
Source: Commun Chem. 2022 Aug 26;5:102. doi: 10.1038/s42004-022-00716-1 (PMC9814726; doi:10.1038/s42004-022-00716-1)
Supplement: Supplementary file 2 — Supplementary Material [file 42004_2022_716_MOESM2_ESM.pdf]

LED-pump-X-ray-multiprobe crystallography for sub-second timescales

Supplementary Information

Lauren E. Hatcher,[1,2] Mark R. Warren,[3] Jonathan M. Skelton,[1,4] Anuradha R. Pallipurath,[1,5] Lucy K. Saunders,[3] David R. Allan,[3] Paul Hathaway,[3] Giulio Crevatin,[3] David Omar,[3] Ben H. Williams,[3] Ben A. Coulson,[2] Chick C. Wilson[1] and Paul R. Raithby[1]*

[1] Department of Chemistry, University of Bath, Bath, UK.

[2] School of Chemistry, Cardiff University, Cardiff, UK.

[3] Diamond Light Source, Harwell Science and Innovation Campus, Didcot, UK.

[4] Department of Chemistry, University of Manchester, Manchester, UK.

[5] School of Chemical and Process Engineering, University of Leeds, Leeds, UK.

* E-mail: p.r.raithby@bath.ac.uk

Supplementary Note 1: Test crystal information

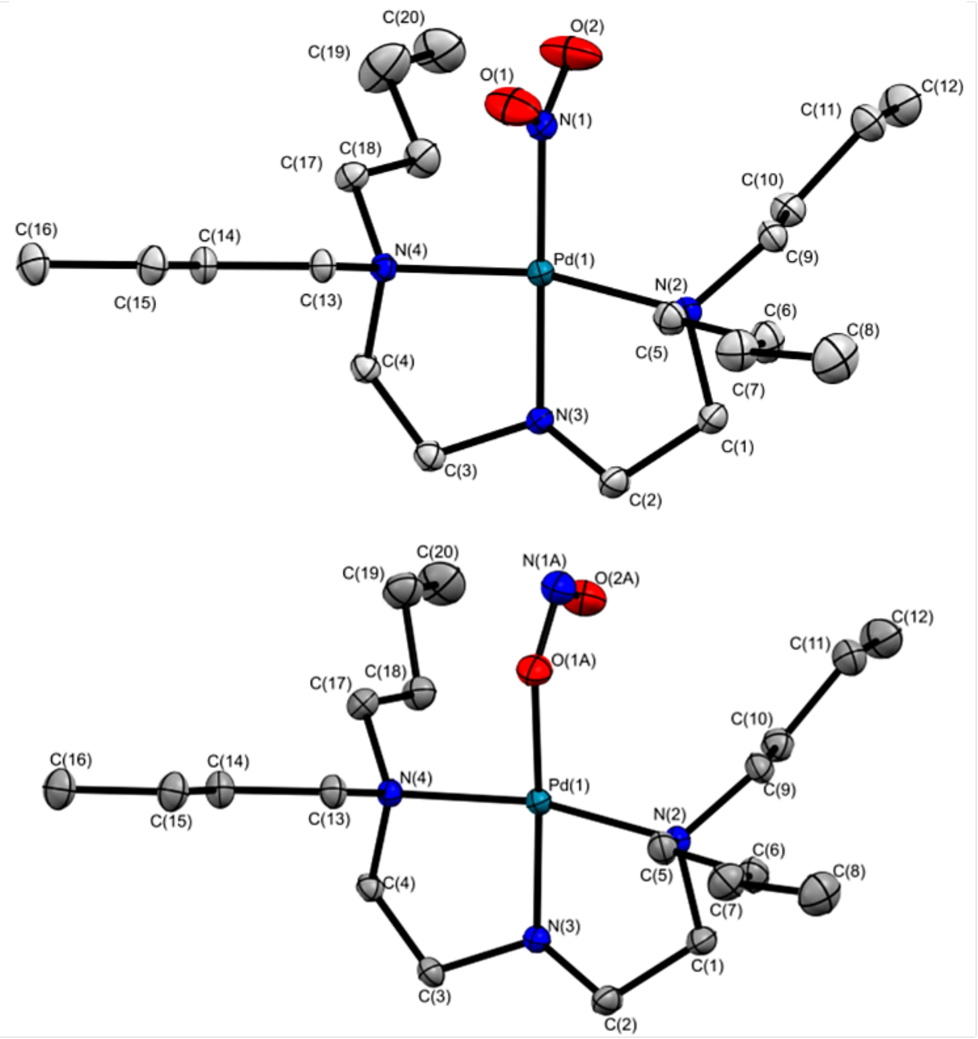

Figure S1 Crystal structures of the photoactive $[Pd(Bu_4dien)(NO_2)]^+$ cation showing the ground-state nitro (η^1-<u>N</u>O_2; top) and excited state *endo*-nitrito (η^1-<u>O</u>NO; bottom) isomers.[1]

Crystal data for GS of 1 at 100 K (100 % nitro-NO₂ isomer): $C_{48}H_{73}B_1N_4O_3Pd_1$ (M = 871.31 g mol^{-1}): monoclinic, space group $P2_1/n$ (no. 14), a = 11.5457(4) Å, b = 13.4021(5) Å, c = 29.7173(12) Å, β = 95.335(4) °, V = 4578.4(3) Å^3, Z = 4, T = 100(2) K, μ(MoK$_\alpha$) = 0.451 mm^{-1}, ρ_{calc} = 1.264 g cm^{-3}, 19,627 reflections measured (6.032° ≤ 2Θ ≤ 52.744°), 9,355 unique (R_{int} = 0.0459) used in solution and refinement. The final R_1 was 0.0428 ($I > 2\sigma(I)$) and wR_2 was 0.0863 (all data).[1]

Crystal data for ES of 1 at 100 K (100 % nitrito-ONO isomer): $C_{48}H_{73}B_1N_4O_3Pd_1$ (M = 871.31 g/mol): monoclinic, space group $P2_1/n$ (no. 14), a = 11.4775(4) Å, b = 13.3647(5) Å, c = 29.9849(8) Å, β = 94.569(3) °, V = 4584.9(3) Å^3, Z = 4, T = 100(2) K, μ(MoK$_\alpha$) = 0.448 mm^{-1}, ρ_{calc} = 1.262 g cm^{-3}, 31,062 reflections measured (6.032° ≤ 2Θ ≤ 52.744°), 9,355 unique (R_{int} = 0.0661) used in solution and refinement. The final R_1 was 0.0428 ($I > 2\sigma(I)$) and wR_2 was 0.0901 (all data).[1]

Supplementary Note 2: Excitation Source

(i) LED sphere design and performance testing

The LEDs in the custom printed sphere array are connected *via* an Adafruit TB6612 driver to a standard laboratory DC power supply (ISO-TECH IPS303DD). The driver powers the LED array in response to a 3.3V TTL signal from a timeframe generator (TFG2), enabling synchronisation between the array and diffractometer through the beamline's Generic Data Acquisition (GDA) software[2] as outlined in the following section. The timing of the LED circuit is monitored through a Tektronix TDS3012B oscilloscope. A circuit diagram for the set-up is included in Figure S2 below.

The LED light pump is synchronised to the diffractometer hardware using the GDA software.[2] Simple electronic triggering provides complete control over the excitation pulse length t_{exc}, allowing us to optimise the excitation period to achieve high photoconversion in the crystal. The length of the decay period, the complete cycle time, and the acquisition period (t_{dec}, t_{cyc} and t_{acq}, respectively) are also flexible, within the limits of the diffractometer, and the timing sequence can be implemented using relatively inexpensive electronics.

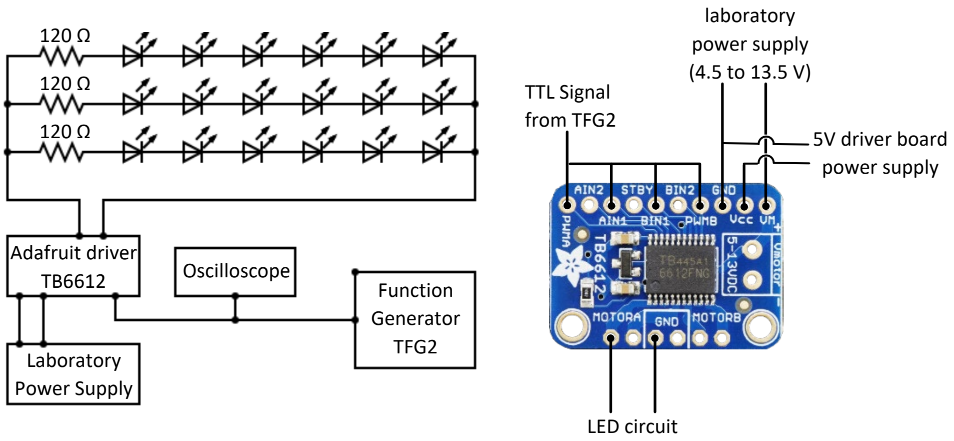

Figure S2 LED sphere circuit. (a) Circuit wiring diagram for LED sphere with connections for time-resolved synchronisation. (b) Photograph of Adafruit TB16612 circuit board used to drive the LED sphere array with connections as marked.

(ii) LED sphere power test experiments

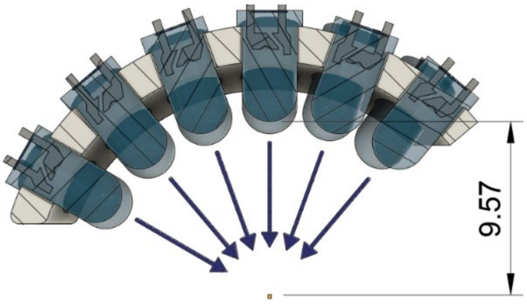

The power provided to the crystal during a typical pump-multiprobe data collection by the LED sphere set-up was recorded using a Thorlabs PM400 optical power meter equipped with a Thorlabs S401C with sensor head. The sensor was placed at the crystal position, at a distance of 9.57 cm from the LED sphere. Obstructing the sensor with a 100 um pin hole provided a sample test area similar to that of the single crystal samples used in pump-multiprobe experiments and gave a power measurement of 23 mW.

(iii) LED sphere array performance test experiments

The pulse separation of LED set-up was tested using an Osram SFH203P silicon pin photodiode (wavelength range 400-1100 nm, switching time 5 ns). The photodiode was placed at a distance of 9.57 cm from the LED array, simulating the position of the crystal during the pump-multiprobe data collection on the diffractometer, and connected to a Tektronix TDS3012B oscilloscope to measure its output. The LEDs were connected as described above, and a series of pulse widths and separations were tested. A reliable pulse separation was recorded down to a resolution of ~ 1 ms , and a rise time of *c.a.* 22 μs and fall time of *c.a.* 400 μs were observed (Figures S3(a) and S3(b), respectively).

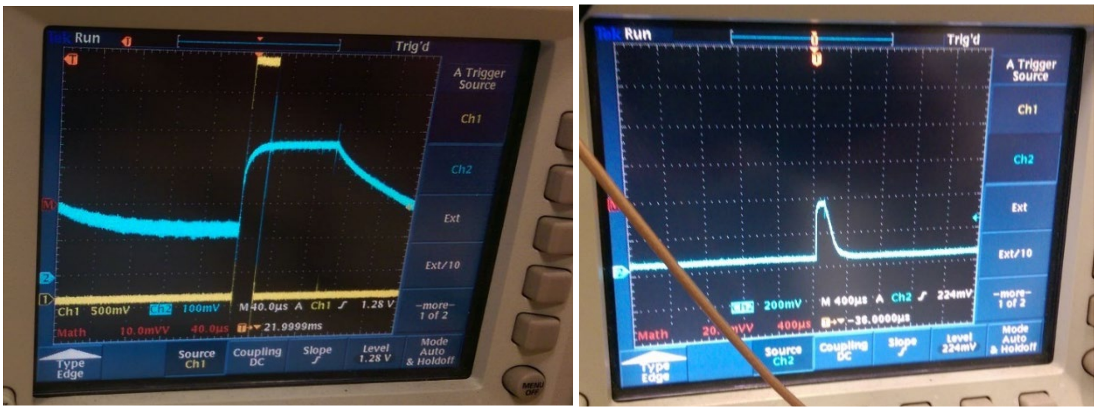

Figure S3 Photograph of the oscilloscope reading showing a pulse rise time of 22 μs (the x-axis displays the time, with 1 graduation = 40 μs). (b) Photograph of the oscilloscope reading confirming the pulse shape for the LED set-up and showing a fall time of approximately 200 μs (the x-axis displays the time, with 1 graduation = 400 μs).

Supplementary Note 3: Sample excitation pre-experiments

(i) X-ray exposure tests

To test for X-ray damage and/or X-ray-induced excitation, crystals of **1** were exposed to the synchrotron X-ray beam at 150 K continuously for 25 mins while five standard single-crystal X-ray datasets were collected. As at this temperature, the thermal decay of the photo-induced nitrito-ONO isomer is negligible so the excited state is cryo-trapped and any build-up over time is thus easily identified by solving and refining a crystal structure from each of the five datasets. The synchrotron X-ray beam was attenuated to 25 % (X-ray test 1) and 5 % (X-ray test 2) of the available flux. The results of these two experiments are provided in Figure S4 and S5 respectively.

While we observed no appreciable crystal degradation in either experiment, both indicated a steady increase in the excited state conversion fraction, $\Delta\alpha(t)$, with time, reaching a maximum of 2.5 and 10 % after 25 min with the 5 and 25 % beam intensity respectively. As a result of these preliminary investigations, we selected the lower 5 % beam intensity for the final pump-multiprobe experiments, as a compromise between obtaining sufficient signal-to-noise from short X-ray exposures and minimising undesirable X-ray induced excitation.

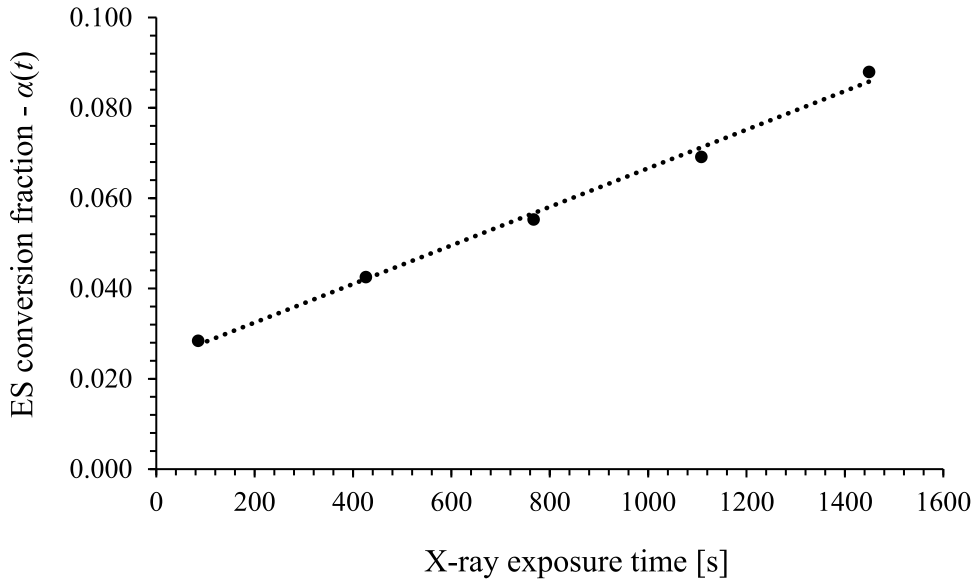

Figure S4 ES conversion fraction $\alpha(t)$ as a function of extended X-ray exposure time, utilising 25 % of the synchrotron beam, showing the steady increase in X-ray induced excitation in **1** with prolonged X-ray exposure (max. 9.1 %).

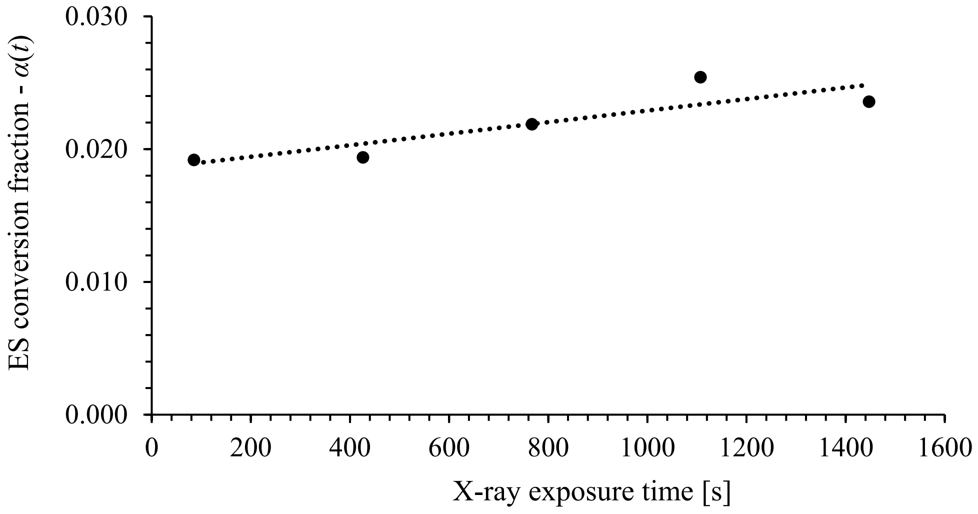

Figure S5 ES conversion fraction $\alpha(t)$ as a function of extended X-ray exposure time, utilising 5 % of the synchrotron beam, showing the steady increase in X-ray induced excitation in **1** with prolonged X-ray exposure (max. 2.5 %).

(ii) LED exposure tests

To assess the extent of any crystal degradation caused by light exposure, a crystal of **1** was mounted on the diffractometer and illuminated continuously using the LED sphere for a period of *c.a.* 4 h at 270 K. At this temperature the photoisomerisation process in **1** is reversible (i.e. the photo-excited state is not cryo-trapped), but continuous illumination produces a measurable steady-state population of the excited state corresponding to *c.a.* 20 % conversion. Complete single-crystal X-ray datasets were collected at regular intervals during the illumination period, utilising 5 % of the available synchrotron beam so as to minimise X-ray induced excitation. Structures were solved and refined by standard procedures, to determine the excited-state conversion fraction as a function of illumination time. The results are shown in Figure S6 below.

There is a gradual reduction in the steady-state $\alpha(t)$ with increasing irradiation time, indicating that a gradual photobleaching process is occurring. After a total of 15,254 s (04:14:05) at the end of the test experiment, $\alpha(t)$ had decreased by 6 %. This change was accompanied by a visible change in the crystal colour from pale yellow at the start of the experiment to mid orange by the end (Figure S7). These observations led us to select a new crystal for each pump-multiprobe data collection in order to minimise the effect of photobleaching in our measurements.

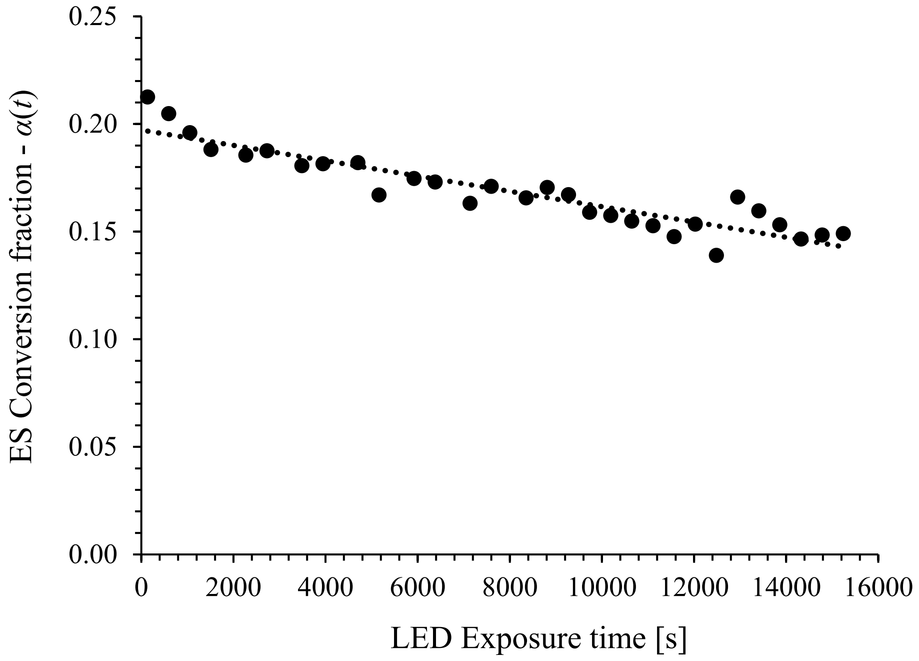

Figure S6 ES conversion fraction $\alpha(t)$ in crystals of 1 as a function of extended LED irradiation time at 270 K, showing the gradual photobleaching with prolonged 400 nm pump light exposure

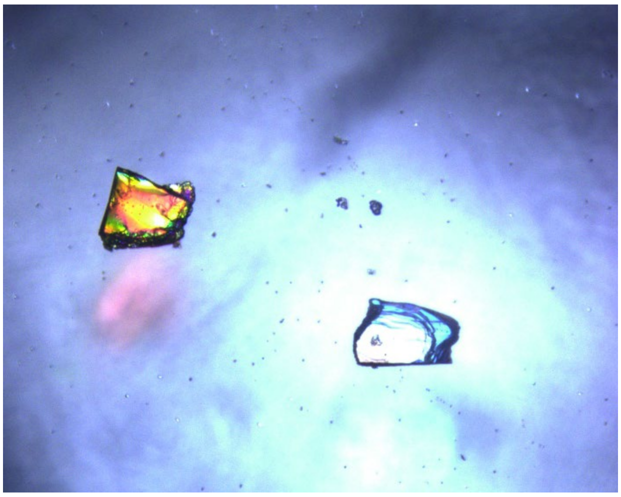

Figure S7 Microscope image of two crystals of **1** before (right) and after (left) illumination with 400 nm LEDs for *c.a.* 4 h at 270 K.

Supplementary Note 4: Preliminary kinetic measurements, model parameterisation and numerical simulations

(i) Preliminary experiments

A series of preliminary X-ray photocrystallography experiments to investigate the excitation and decay kinetics in single-crystals of **1** were carried out following the procedures outlined in our previous work.[3] The excitation kinetics were measured at 150 K, where the excited state is cryo-trapped, and the decay kinetics were measured between 240 and 270 K. The results of these experiments are outlined in Tables S1-S11 below. In addition, a series of pseudo-steady-state photocrystallographic measurements were performed where a crystal was subject to continuous illumination while complete X-ray datasets were collected to measure the steady-state ES occupations α_{SS} between 250 and 300 K. These data are summarised in Table S12.

Table S1 Excitation kinetic experiments on **1** performed at 150 K. Full single-crystal X-ray datasets were collected and refined at regular time intervals after a set cumulative excitation time to obtain values for the excitation state conversion fraction, $\alpha(t)$, with respect to irradiation time t.

Excitation time [s]	Refined GS nitro-NO_2 occupancy	Refined ES nitrito-ONO occupancy (ES conversion fraction, $\alpha(t)$)
1	0.97	0.03
2	0.95	0.05
4	0.93	0.07
8	0.88	0.12
16	0.79	0.21
32	0.62	0.38
64	0.36	0.64
128	0.13	0.87

Table S2 Decay kinetic experiments on **1** performed at 240 K. After complete excitation at 100 K, the temperature was raised to 240 K and a series of full single-crystal X-ray datasets were collected and refined at regular time intervals to obtain values for the excitation state conversion fraction, $\alpha(t)$, with respect to decay time.

Decay time [s]	Refined GS nitro-NO_2 occupancy	Refined ES nitrito-ONO occupancy (ES conversion fraction, $\alpha(t)$)
0.0	0.00	1.00
85	0.24	0.76
426	0.40	0.60
766	0.53	0.47
1106	0.64	0.36
1448	0.71	0.29

Table S3 Decay kinetic experiments on **1** performed at 242.5 K. After complete excitation at 100 K, the temperature was raised to 242.5 K and a series of full single-crystal X-ray datasets were collected and refined at regular time intervals to obtain values for the excitation state conversion fraction, $\alpha(t)$, with respect to decay time.

Decay time [s]	Refined GS nitro-NO_2 occupancy	Refined ES nitrito-ONO occupancy (ES conversion fraction, $\alpha(t)$)
0.0	0.00	1.00
139.0	0.30	0.70
599.0	0.61	0.39
1057.0	0.78	0.22
1515.0	0.86	0.14
1975.0	0.91	0.09

Table S4 Decay kinetic experiments on **1** performed at 245 K. After complete excitation at 100 K, the temperature was raised to 245 K and a series of full single-crystal X-ray datasets were collected and refined at regular time intervals to obtain values for the excitation state conversion fraction, $\alpha(t)$, with respect to decay time.

Decay time [s]	Refined GS nitro-NO_2 occupancy	Refined ES nitrito-ONO occupancy (ES conversion fraction, $\alpha(t)$)
0.0	0.00	1.00
85.0	0.26	0.74
426.0	0.56	0.44
767.0	0.75	0.25
1108.0	0.85	0.15
1449.0	0.91	0.09

Table S5 Decay kinetic experiments on **1** performed at 247.5 K. After complete excitation at 100 K, the temperature was raised to 247.5 K and a series of full single-crystal X-ray datasets were collected and refined at regular time intervals to obtain values for the excitation state conversion fraction, $\alpha(t)$, with respect to decay time.

Decay time [s]	Refined GS nitro-NO_2 occupancy	Refined ES nitrito-ONO occupancy (ES conversion fraction, $\alpha(t)$)
0.0	0.00	1.00
139.0	0.44	0.56
599.0	0.81	0.19
1058.0	0.93	0.07
1517.0	0.95	0.05
1976.0	0.96	0.04

Table S6 Decay kinetic experiments on **1** performed at 250 K. After complete excitation at 100 K, the temperature was raised to 250 K and a series of full single-crystal X-ray datasets were collected and refined at regular time intervals to obtain values for the excitation state conversion fraction, $\alpha(t)$, with respect to decay time.

Decay time [s]	Refined GS nitro-NO_2 occupancy	Refined ES nitrito-ONO occupancy (ES conversion fraction, $\alpha(t)$)
0.0	0.00	1.00
85.0	0.54	0.46
425.0	0.83	0.17
765.0	0.92	0.08
1106.0	0.94	0.06
1447.0	0.96	0.04

Table S7 Decay kinetic experiments on **1** performed at 252.5 K. After complete excitation at 100 K, the temperature was raised to 252.5 K and a series of full single-crystal X-ray datasets were collected and refined at regular time intervals to obtain values for the excitation state conversion fraction, $\alpha(t)$, with respect to decay time.

Decay time [s]	Refined GS nitro-NO_2 occupancy	Refined ES nitrito-ONO occupancy (ES conversion fraction, $\alpha(t)$)
0.0	0.00	1.00
139.0	0.50	0.50
597.0	0.94	0.06
1056.0	0.97	0.03
1516.0	0.97	0.03
1975.0	0.96	0.04

Table S8 Decay kinetic experiments on **1** performed at 255 K. After complete excitation at 100 K, the temperature was raised to 255 K and a series of full single-crystal X-ray datasets were collected and refined at regular time intervals to obtain values for the excitation state conversion fraction, $\alpha(t)$, with respect to decay time.

Decay time [s]	Refined GS nitro-NO_2 occupancy	Refined ES nitrito-ONO occupancy (ES conversion fraction, $\alpha(t)$)
0.0	0.00	1.00
85.0	0.61	0.39
427.0	0.94	0.06
767.0	0.97	0.03
1109.0	0.96	0.04
1450.0	0.97	0.03

Table S9 Decay kinetic experiments on **1** performed at 257.5 K. After complete excitation at 100 K, the temperature was raised to 257.5 K and a series of full single-crystal X-ray datasets were collected and refined at regular time intervals to obtain values for the excitation state conversion fraction, $\alpha(t)$, with respect to decay time.

Decay time [s]	Refined GS nitro-NO_2 occupancy	Refined ES nitrito-ONO occupancy (ES conversion fraction, $\alpha(t)$)
0.0	0.00	1.00
138.0	0.85	0.15
598.0	0.96	0.04
1058.0	0.97	0.03
1516.0	0.97	0.03
1976.0	0.97	0.03

Table S10 Decay kinetic experiments on **1** performed at 260 K. After complete excitation at 100 K, the temperature was raised to 260 K and a series of full single-crystal X-ray datasets were collected and refined at regular time intervals to obtain values for the excitation state conversion fraction, $\alpha(t)$, with respect to decay time.

Decay time [s]	Refined GS nitro-NO_2 occupancy	Refined ES nitrito-ONO occupancy (ES conversion fraction, $\alpha(t)$)
0.0	0.00	1.00
85.0	0.88	0.12
426.0	0.96	0.04
767.0	0.96	0.04
1107.0	0.96	0.04
1448.0	0.95	0.05

Table S11 Decay kinetic experiments on **1** performed at 270 K. After complete excitation at 100 K, the temperature was raised to 270 K and a series of full single-crystal X-ray datasets were collected and refined at regular time intervals to obtain values for the excitation state conversion fraction, $\alpha(t)$, with respect to decay time.

Decay time [s]	Refined GS nitro-NO_2 occupancy	Refined ES nitrito-ONO occupancy (ES conversion fraction, $\alpha(t)$)
0.0	0.00	1.00
85.0	0.95	0.05
425.0	0.95	0.05
765.0	0.95	0.05
1106.0	0.95	0.05
1448.0	0.95	0.05

Table S12 Pseudo-steady-state photocrystallographic measurements performed on **1** between 250 and 300 K. A crystal of 1 was continuously illuminated and a series of full single-crystal X-ray datasets were collected at several temperature intervals between 250 and 300 K. The data were refined to obtain values for the steady-state conversion fraction to the excited state with respect to temperature, $\alpha_{SS}(T)$.

Temperature [K]	Refined GS nitro-NO_2 occupancy	Refined ES nitrito-ONO occupancy (ES conversion fraction, $\alpha_{SS}(T)$)
250	0.16	0.84
260	0.25	0.75
270	0.58	0.42
272	0.68	0.32
274	0.73	0.27
276	0.78	0.22
278	0.80	0.20
280	0.84	0.16
290	0.91	0.09
300	0.93	0.07

(ii) Kinetic model parameterisation and numerical simulations

The kinetic measurements in Tables S1-S11 were fitted to the Johnson-Mehl-Avrami-Kohnogorov (JMAK) kinetic model:[4-6]

$$\alpha(t) = \alpha_\infty + (\alpha_0 - \alpha_\infty)\exp[-kt^n]$$

where $\alpha(t)$ is the time-dependent population of the excited state, α_0 and α_∞ are the initial and final ES populations, k is the rate constant, and n is the Avrami exponent. n is related to the dimensionality D of the transformation as $D = n - 1$. For linkage-isomer systems it is accepted that the isomerisation is non-cooperative and occurs homogenously throughout the crystal bulk, and thus it is common practice to fix n = 1, which we do here.[3] Figures S8 and S9 show the JMAK fits to the excitation and decay measurements in Tables S1 and S2-S11, respectively, and the fit parameters are collected in Table S13.

The decay rate constant k_{dec} is strongly temperature dependent, and this temperature dependence is usually well described by the Arrhenius law:

$$k(T) = A\exp[-E_{\mathrm{A}}/RT]$$

$$\ln[k(T)] = -\frac{E_A}{R}\frac{1}{T}$$

where E_A is the activation energy and the pre-exponential factor A can be roughly equated to an attempt frequency. An analysis of the decay rates in Table S13 using the linearised Arrhenius equation is shown in Figure S10 and yields an activation energy E_A of 74.2 kJ mol^{-1} and an attempt frequency ln$A$ of 30.6, which are both in line with our previous kinetic study on this system.[3]

Using the excitation rate constant, which is assumed to be independent of temperature, and the Arrhenius parameterisation of the decay rate, it is possible to set up a numerical simulation to predict the time evolution of the ES population, $\alpha(t)$, under different conditions.[3] We assume that the excitation and decay processes are independent over a short time interval Δt, which we choose such that the change in α, $\Delta\alpha$, is $< 10^{-4}$, and use the appropriate JMAK equations to update the populations for the following timestep.

These simulations can be used to predict the α reached at photostationary equilibrium under continuous illumination at a given temperature, or to predict the dynamic behaviour during a simulated pump-probe cycle. (In the latter case, we run sufficient simulated cycles for the populations at the start/end of the cycle to stabilise, which typically takes 2-3 cycles.)

Using these numerical simulations, we first refine our initial excitation rate and Arrhenius parameterisation against the measured pseudo-steady-state ES population as a function of temperature (Table S12, Figure S11). The refinement was performed in two stages: first, the excitation rate constant k_{exc} was refined, and second, k_{exc} and the Arrhenius parameters for the decay rate were refined. The final model parameters are listed in Table S14.

With this parameterisation, we then performed for each of the pump-probe cycle times t_{cyc}, *viz.* 170, 108, 35, 22 and 14 s, a series of simulations in which the excitation time t_{exc} and temperature T were varied in order to estimate the maximum and minimum ES populations, α_{min} and α_{max}, at $t = 0$ and $t = t_{exc}$, and hence the difference $\Delta\alpha = \alpha_{\mathrm{max}} - \alpha_{\mathrm{min}}$. The results of these simulations are shown in Figures S12-S16.

For each t_{cyc} selected, the model predicts the optimum t_{exc}, t_{dec} and T subject to two conditions: (1) complete ES decay between sequential cycles; and (2) maximising the difference $\Delta\alpha$ between the start of the cycle at $t = 0$ and the end of the excitation period at $t = t_{exc}$. These are tabulated in Table S15. In general, the trade-off here is that measuring at a lower temperature and allowing for incomplete decay between pump-probe cycles allows for a larger $\Delta\alpha$, which is mainly due to the exponential nature of the decay process.[2] However, it is generally desirable to have complete decay so that at least one of the X-ray datasets is a clean ground-state structure to use as a reference point for e.g. generating photo-difference maps.

The excitation rate k_{exc} depends very strongly on the crystal size and morphology,[3] so these parameters are only a rough guide. In practice, we found that the two predictions provided a good guide to the optimum t_{exc} and t_{dec} and a window of temperatures. Using these, we were able to select pump-multiprobe cycle timings and rapidly optimise the measurement temperature through experimentation while collecting datasets.

Table S13 Kinetic parameters obtained by fitting the experimental data in Tables S1-S11 to the Johnson-Mehl-Avrami-Kohnogorov (JMAK) model: α_0 - initial excited-state occupation; α_∞ final ES occupation; k - rate constant; n - Avrami exponent; RMS - root-mean-square fitting error.

Type	T [K]	α_0 [%]	α_∞ [%]	k [s^{-n}]	n	RMS [%]
Exc.	150.0	1.19	100.0	1.54×10^{-2}	1	0.98
Dec.	240.0	93.6	24.2	1.62×10^{-3}	1	4.62
Dec.	242.5	96.5	9.11	1.94×10^{-3}		3.15
Dec.	245.0	95.5	6.60	2.16×10^{-3}		3.46
Dec.	247.5	98.6	5.76	3.98×10^{-3}		2.52
Dec.	250.0	99.6	8.21	1.00×10^{-2}		3.66
Dec.	252.5	100	2.93	5.27×10^{-3}		0.66
Dec.	255.0	100	3.82	1.18×10^{-2}		0.80
Dec.	257.5	100	3.25	1.53×10^{-2}		0.35
Dec.	260.0	100	4.25	2.96×10^{-2}		0.35

Table S14 Refined kinetic model parameters for the numerical simulations in Figures S12-S16 and Table S13: k_{exc} - excitation rate constant; E_{A}/ ln$A$ activation energy and pre-exponential factor for the Arrhenius parameterisation of the temperature-dependent decay rate constant $k_{\text{dec}}(T)$; n - Avrami exponent for the excitation and decay processes.

Parameter	Value
k_{exc} [s^{-1}]	4.35×10^{-2}
E_{A} [kJ mol^{-1}]	74.3
ln$A$	30.4
n	1

Table S15 Predicted parameters for pump-multiprobe experiments with the five cycle times t_{cyc} used in the experiments, *viz.* 170, 108, 35, 22 and 14 s, obtained from the numerical simulations in Figures S12-S16. For each cycle time, the optimised excitation and decay times, $t_{\text{exc}}/t_{\text{dec}}$, measurement temperature T, and the minimum and maximum excited-state population α at $t = 0$ and $t = t_{\text{exc}}$ and the difference, $\Delta\alpha$, are listed for two conditions: (1) complete excited-state decay between sequential cycles, and (2) maximum $\Delta\alpha$.

t_{cyc} [s]	t_{exc} [s]	t_{dec} [s]	T [K]	$\alpha(t = 0)$ [%]	$\alpha(t = t_{\text{exc}})$ [%]	$\Delta\alpha$ [%]
170	52.7	117.3	260.0	7.8	67.9	60.1
	34.0	136.0	269.5	0.0	40.2	40.2
108	37.8	70.2	263.0	8.6	57.9	49.3
	22.7	85.3	273.0	0.0	30.2	30.2
35	15.0	20.0	270.0	9.1	33.9	24.8
	8.1	26.9	282.0	0.0	12.8	12.8
22	9.9	12.1	272.5	9.0	26.4	17.4
	5.7	16.3	286.0	0.0	8.8	8.8
14	6.4	7.6	275.5	7.4	19.5	12.0
	3.5	10.5	289.5	0.0	6.0	6.0

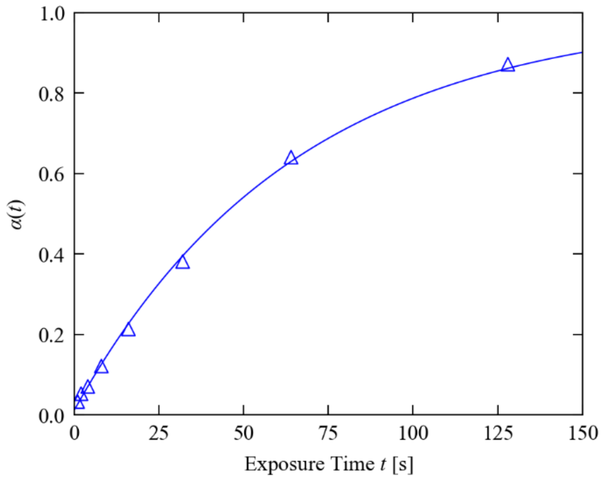

Figure S8 Johnson-Mehl-Avrami-Kohnogorov (JMAK) fit to the excitation kinetic data in Table S1. The markers show the experimental measurements and the solid line shows the fit. The fit parameters are listed in Table S14.

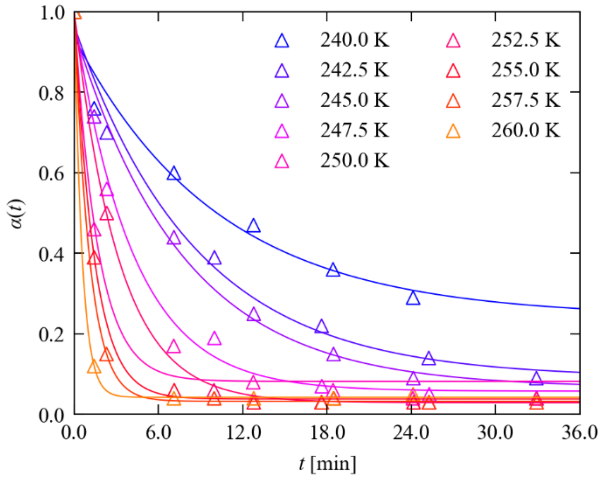

Figure S9 Johnson-Mehl-Avrami-Kohnogorov (JMAK) fit to the decay kinetic data in Tables S2-S11. The markers show the experimental measurements and the solid line shows the fit, and each set of data is coloured from blue (250 K) to yellow (260 K). Note that in the 270 K measurement the decay was too rapid to obtain a meaningful fit. The fit parameters are listed in Table S14.

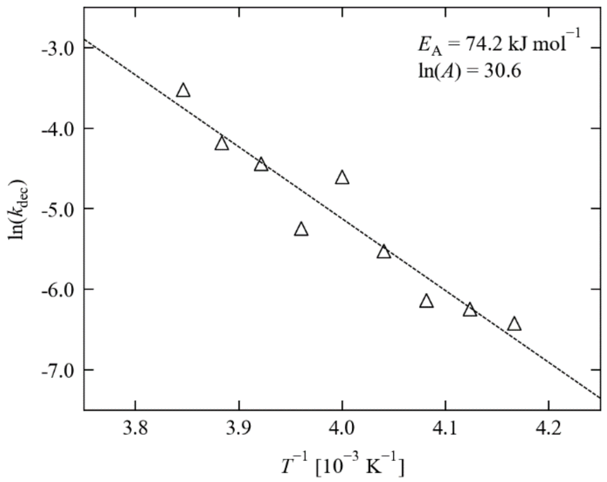

Figure S10 Arrhenius analysis of the temperature dependence of the decay rate constants in Table S13. The markers show the experimental data and the dashed line shows the fit to the linearised Arrhenius equation with the parameters indicated.

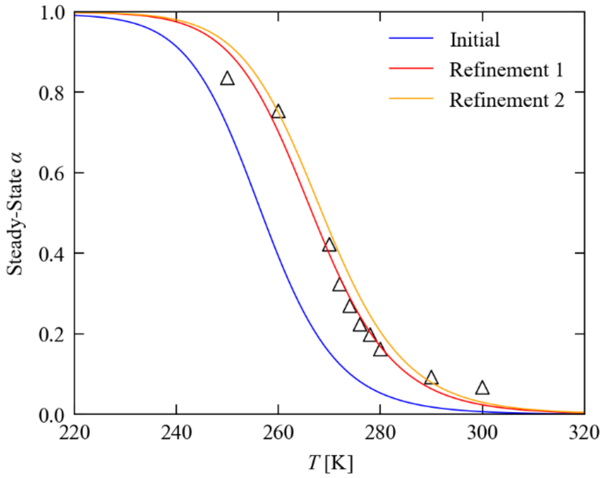

Figure S11 Refinement of the two-process JMAK model against the steady-state measurements in Table S12. The markers show the experimental measurements. The blue line shows the predicted temperature dependence of the steady-state excited-state population, α_{SS}, obtained using the excitation rate constant from the JMAK fit in Figure S8 and the Arrhenius parameters from the analysis in Figure S10. The red line shows the predicted dependence after refinement of the initial excitation rate constant ("Refinement 1"), and the yellow line shows the dependence after refinement of both the excitation rate and the Arrhenius parameters ("Refinement 2"). The final model parameters used to perform the initial numerical simulations of the pump-probe cycles are listed in Table S14.

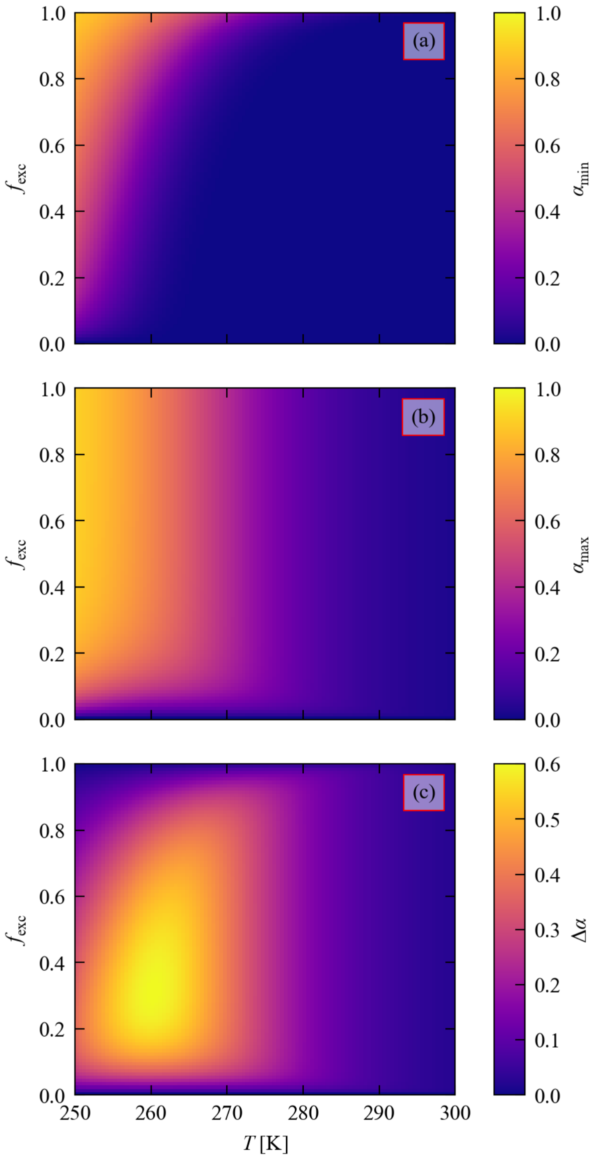

Figure S12 Numerical simulations to optimise the excitation and decay times, t_{exc}/t_{dec}, and measurement temperature T for a fixed pump-probe cycle time of t_{cyc} = 170 s. The kinetic parameters for the two-process JMAK model are listed in Table S14. (a) Minimum excited-state occupation, α_{min}, at $t = 0$, as a function of the fraction f_{exc} of t_{cyc} used for the excitation ($t_{exc} = f_{exc} \times t_{cyc}$ and $t_{dec} = t_{cyc} - t_{exc} = (1 - f_{exc}) \times t_{cyc}$). (b) Maximum ES occupation, α_{max}, at $t = t_{exc}$. (c) Difference $\Delta\alpha = \alpha_{max} - \alpha_{min}$.

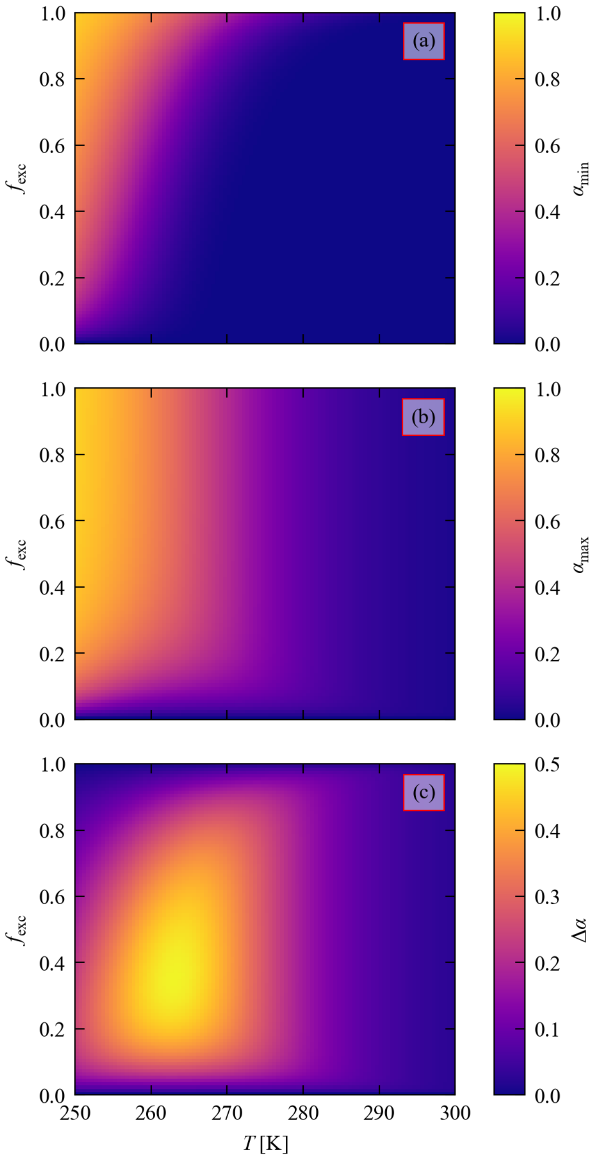

Figure S13 Numerical simulations to optimise the excitation and decay times, t_{exc}/t_{dec}, and measurement temperature T for a fixed pump-probe cycle time of t_{cyc} = 108 s. The kinetic parameters for the two-process JMAK model are listed in Table S14. (a) Minimum excited-state occupation, α_{min}, at $t = 0$, as a function of the fraction f_{exc} of t_{cyc} used for the excitation ($t_{exc} = f_{exc} \times t_{cyc}$ and $t_{dec} = t_{cyc} - t_{exc} = (1 - f_{exc}) \times t_{cyc}$). (b) Maximum ES occupation, α_{max}, at $t = t_{exc}$. (c) Difference $\Delta\alpha = \alpha_{max} - \alpha_{min}$.

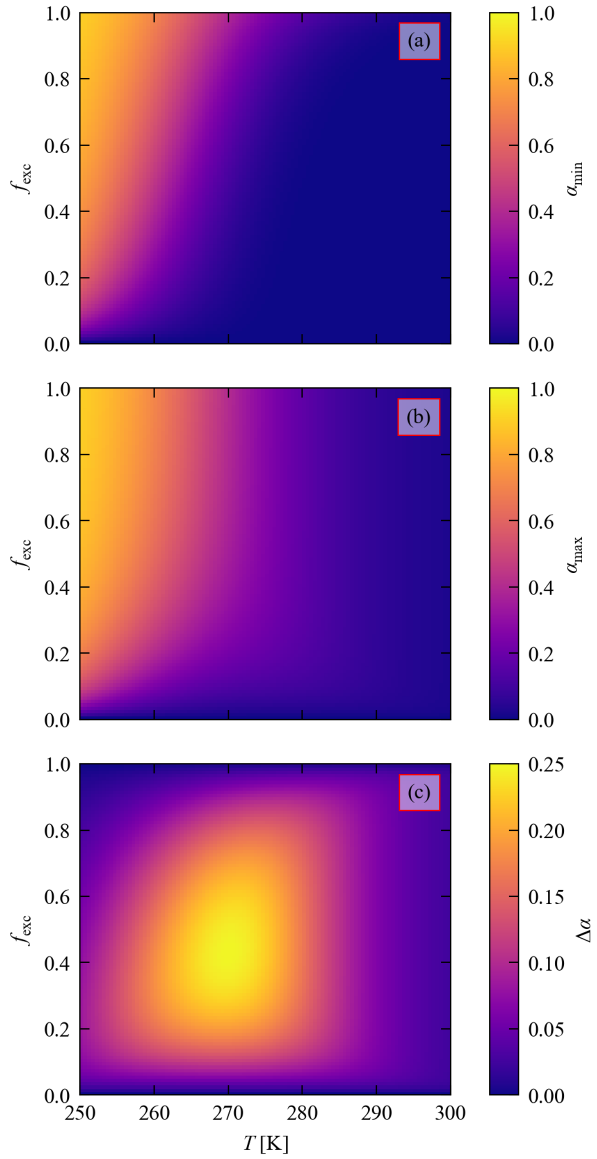

Figure S14 Numerical simulations to optimise the excitation and decay times, t_{exc}/t_{dec}, and measurement temperature T for a fixed pump-probe cycle time of t_{cyc} = 35 s. The kinetic parameters for the two-process JMAK model are listed in Table S14. (a) Minimum excited-state occupation, α_{min}, at $t = 0$, as a function of the fraction f_{exc} of t_{cyc} used for the excitation ($t_{exc} = f_{exc} \times t_{cyc}$ and $t_{dec} = t_{cyc} - t_{exc} = (1 - f_{exc}) \times t_{cyc}$). (b) Maximum ES occupation, α_{max}, at $t = t_{exc}$. (c) Difference $\Delta\alpha = \alpha_{max} - \alpha_{min}$.

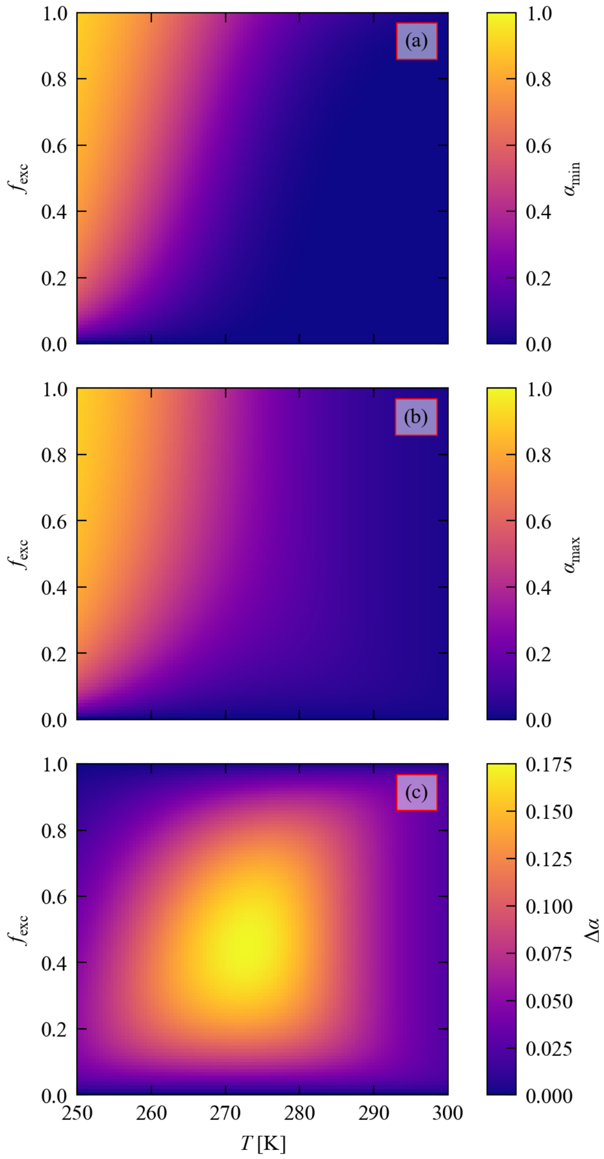

Figure S15 Numerical simulations to optimise the excitation and decay times, t_{exc}/t_{dec}, and measurement temperature T for a fixed pump-probe cycle time of t_{cyc} = 22 s. The kinetic parameters for the two-process JMAK model are listed in Table S14. (a) Minimum excited-state occupation, α_{min}, at $t = 0$, as a function of the fraction f_{exc} of t_{cyc} used for the excitation ($t_{exc} = f_{exc} \times t_{cyc}$ and $t_{dec} = t_{cyc} - t_{exc} = (1 - f_{exc}) \times t_{cyc}$). (b) Maximum ES occupation, α_{max}, at $t = t_{exc}$. (c) Difference $\Delta\alpha = \alpha_{max} - \alpha_{min}$.

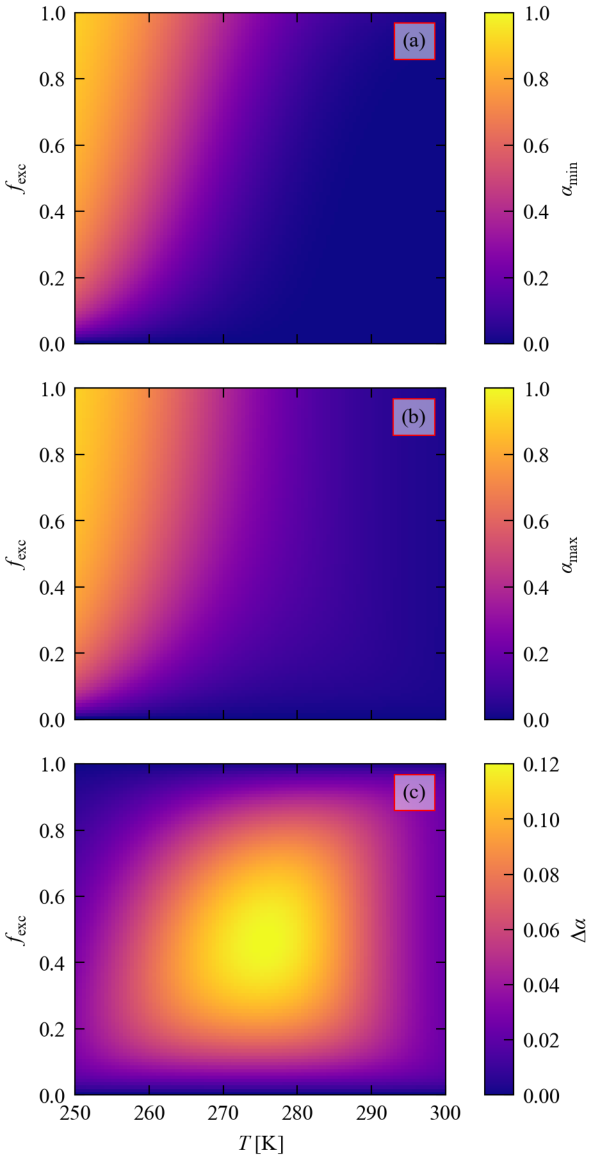

Figure S16 Numerical simulations to optimise the excitation and decay times, t_{exc}/t_{dec}, and measurement temperature T for a fixed pump-probe cycle time of t_{cyc} = 14 s. The kinetic parameters for the two-process JMAK model are listed in Table S14. (a) Minimum excited-state occupation, α_{min}, at $t = 0$, as a function of the fraction f_{exc} of t_{cyc} used for the excitation ($t_{exc} = f_{exc} \times t_{cyc}$ and $t_{dec} = t_{cyc} - t_{exc} = (1 - f_{exc}) \times t_{cyc}$). (b) Maximum ES occupation, α_{max}, at $t = t_{exc}$. (c) Difference $\Delta\alpha = \alpha_{max} - \alpha_{min}$.

Supplementary Note 5: Pump-multiprobe experimental data

Table S16 Summary of the 12 LED-pump-X-ray-probe datasets collected on **1**. The maximum conversion percentages, α_{max} or $\alpha(t = t_{exc})$, are the nitrito-(η^1-ONO) isomer occupancy achieved in each crystal after the irradiation period t_{exc}, and were determined from the single-crystal structure refinement. The variation in α between experiments with identical t_{exc} reflect the strong temperature dependence of the decay rate but also the large influence of the crystal size and shape on the photoconversion level. The shaded cells mark the experiments with the highest α discussed in the text and used to generate molecular movies.

t_{acq} [s]	Repeat	T [K]	t_{cyc} [s]	t_{exc} [s]	t_{dec} [s]	$\alpha(t = t_{exc})$ [%]
8	1	260	170	55	115	21.0
	2	265				33.7
	3	270				16.5
4	1	265	108	35	73	21.3
	2	270				18.8
	3	272				21.3
	4	274				20.7
1.6	1	280	35	14	21	10.7
0.8	1	280	22	8	14	11.8
0.4	1	282	14	5	9	8.7
	2	283				10.4
	3	284				8.0

Table S17 Single-crystal X-ray data for selected pump-multiprobe data collections. Crystal data for the structures with the highest and lowest excited-state population α in each pump-multiprobe experiment are given. The average level of X-ray induced excitation across the experiments was ~ 4 %; where the lowest measured α in each pair of entries are significantly above this level this implies the excited state had not fully decayed the selected during t_{cyc}.

**t_{acq} [s]**	**8**	**8**	**8**	**8**	**8**	**8**	**4**	**4**
**Repeat**	**3**	**3**	**2**	**2**	**1**	**1**	**1**	**1**
***T* [K]**	**260**	**260**	**265**	**265**	**270**	**270**	**265**	**265**
Experiment	260K_8s_t5_excit	260K_8s_t17_decay	265K_8s_t5_excit	265K_8s_t17_decay	270K_8s_t5_excit	270K_8s_t17_decay	265K_4s_t6_excit	265K_4s_t19_decay
t_{exc} [s]	55	55	55	55	55	55	35	35
ES conversion α [%]	21.0	9.1	33.7	5.1	16.5	3.3	21.3	7.3
Empirical formula	$C_{48}H_{73}BN_4O_3Pd$	$C_{48}H_{73}BN_4O_3Pd$	$C_{48}H_{73}BN_4O_3Pd$	$C_{48}H_{73}BN_4O_3Pd$	$C_{48}H_{73}BN_4O_3Pd$	$C_{48}H_{73}BN_4O_3Pd$	$C_{48}H_{73}BN_4O_3Pd$	$C_{48}H_{73}BN_4O_3Pd$
Formula weight [g mol^{-1}]	871.31	871.31	871.31	871.31	871.31	871.31	871.31	871.31
Crystal system	monoclinic	monoclinic	monoclinic	monoclinic	monoclinic	monoclinic	monoclinic	monoclinic
Space group	$P2_1/n$	$P2_1/n$	$P2_1/n$	$P2_1/n$	$P2_1/n$	$P2_1/n$	$P2_1/n$	$P2_1/n$
a [Å]	11.6748(6)	11.6715(4)	11.6547(3)	11.6715(4)	11.6667(3)	11.6773(4)	11.6550(4)	11.6668(3)
b [Å]	13.5924(7)	13.5688(4)	13.5866(4)	13.5707(4)	13.5838(4)	13.5796(4)	13.5702(4)	13.5637(5)
c [Å]	30.2436(15)	30.2352(10)	30.2126(8)	30.1533(10)	30.2270(9)	30.2007(10)	30.1920(9)	30.1798(10)
α [°]	90	90	90	90	90	90	90	90
β [°]	93.022(5)	93.267(3)	93.407(3)	93.711(3)	93.269(3)	93.408(3)	93.362(3)	93.535(3)
γ [°]	90	90	90	90	90	90	90	90
Volume [Å^3]	4792.6(4)	4780.5(3)	4775.6(2)	4766.0(3)	4782.5(2)	4780.5(3)	4767.0(3)	4766.7(3)
Z	4	4	4	4	4	4	4	4
ρ_{calc} [g cm^{-3}]	1.208	1.211	1.212	1.214	1.210	1.211	1.214	1.214
μ [mm^{-1}]	0.201	0.202	0.202	0.202	0.201	0.202	0.202	0.202
F(000)	1856.0	1856.0	1856.0	1856.0	1856.0	1856.0	1856.0	1856.0
Crystal size [mm^3]	0.1 × 0.1 × 0.05	0.1 × 0.1 × 0.05	0.1 × 0.1 × 0.05	0.1 × 0.1 × 0.05	0.1 × 0.1 × 0.05	0.1 × 0.1 × 0.05	0.1 × 0.1 × 0.05	0.1 × 0.1 × 0.05
Wavelength λ [Å]	0.534	0.534	0.534	0.534	0.534	0.534	0.534	0.534
2θ range [°]	2.468 to 38.992	2.472 to 38.992	2.47 to 38.992	2.474 to 38.992	2.47 to 38.99	2.472 to 38.992	2.472 to 38.994	2.758 to 38.99
Reflections collected	31360	34381	34211	34237	34353	34400	32582	32656
Independent reflections	9653 [R_{int} = 0.0786, R_{sigma} = 0.1181]	9740 [R_{int} = 0.0883, R_{sigma} = 0.1121]	9727 [R_{int} = 0.0857, R_{sigma} = 0.1113]	9700 [R_{int} = 0.1020, R_{sigma} = 0.1102]	9742 [R_{int} = 0.0863, R_{sigma} = 0.1129]	9737 [R_{int} = 0.0896, R_{sigma} = 0.1072]	9716 [R_{int} = 0.0814, R_{sigma} = 0.1017]	9718 [R_{int} = 0.0971, R_{sigma} = 0.1079]
Goodness of Fit on F^2	0.847	0.864	0.886	0.941	0.857	0.874	0.895	0.899
Final R indices $I \geq 2\sigma(I)$	R_1 = 0.0576, wR_2 = 0.1227	R_1 = 0.0576, wR_2 = 0.1235	R_1 = 0.0563, wR_2 = 0.1120	R_1 = 0.0596, wR_2 = 0.1218	R_1 = 0.0571, wR_2 = 0.1200	R_1 = 0.0581, wR_2 = 0.1258	R_1 = 0.0543, wR_2 = 0.1188	R_1 = 0.0560, wR_2 = 0.1215
Final R indices all data	R_1 = 0.1500, wR_2 = 0.1511	$\mathrm{R_1}$ = 0.1310, $\mathrm{wR_2}$ = 0.1479	R_1 = 0.1191, wR_2 = 0.1314	R_1 = 0.1057, wR_2 = 0.1416	R_1 = 0.1311, wR_2 = 0.1433	R_1 = 0.1205, wR_2 = 0.1495	R_1 = 0.1105, wR_2 = 0.1382	R_1 = 0.0988, wR_2 = 0.1408
Largest diff. peak/hole	0.31/-0.40	0.35/-0.31	0.46/-0.43	0.53/-0.53	0.35/-0.67	0.35/-0.62	0.49/-0.65	0.39/-0.65

Table S17 *cont.* Single-crystal X-ray data for selected pump-multiprobe data collections. Crystal data for the structures with the highest and lowest excited-state population α in each pump-multiprobe experiment are given. The average level of X-ray induced excitation across the experiments was ~ 4 %; where the lowest measured α in each pair of entries are significantly above this level this implies the excited state had not fully decayed during the selected t_{cyc}.

t_{acq} [s]	4	4	4	4	4	4	1.6	1.6
**Repeat**	**2**	**2**	**3**	**3**	**4**	**4**	**1**	**1**
***T* [K]**	**270**	**270**	**272**	**272**	**274**	**274**	**280**	**280**
Experiment	270K_4s_t6_excit	270K_4s_t19_decay	272K_4s_t6_excit	272K_4s_t19_decay	274K_4s_t6_excit	274K_4s_t19_decay	280K_1.6s_t5_excit	280K_1.6s_t13_decay
t_{exc} [s]	35	35	35	35	35	35	14	14
ES conversion, α [%]	18.8	3.2	21.3	4.2	20.7	3.8	10.7	2.5
Empirical formula	$C_{48}H_{73}BN_4O_3Pd$	$C_{48}H_{73}BN_4O_3Pd$	$C_{48}H_{73}BN_4O_3Pd$	$C_{48}H_{73}BN_4O_3Pd$	$C_{48}H_{73}BN_4O_3Pd$	$C_{48}H_{73}BN_4O_3Pd$	$C_{48}H_{73}BN_4O_3Pd$	$C_{48}H_{73}BN_4O_3Pd$
Formula weight [g mol^{-1}]	871.31	871.31	871.31	871.31	871.31	871.31	871.31	871.31
Crystal system	monoclinic	monoclinic	monoclinic	monoclinic	monoclinic	monoclinic	monoclinic	monoclinic
Space group	$P2_1/n$	$P2_1/n$	$P2_1/n$	$P2_1/n$	$P2_1/n$	$P2_1/n$	$P2_1/n$	$P2_1/n$
a [Å]	11.6864(4)	11.6889(4)	11.6632(4)	11.6765(4)	11.6703(5)	11.6836(5)	11.6850(4)	11.6892(5)
b [Å]	13.6030(4)	13.5919(5)	13.5810(4)	13.5820(5)	13.5825(5)	13.5816(5)	13.5977(5)	13.5959(5)
c [Å]	30.2776(10)	30.2410(11)	30.1872(10)	30.1766(10)	30.1953(11)	30.1824(10)	30.1995(10)	30.1834(10)
α [°]	90	90	90	90	90	90	90	90
β [°]	93.416(3)	93.595(3)	93.304(3)	93.521(3)	93.343(3)	93.541(3)	93.262(3)	93.352(3)
γ [°]	90	90	90	90	90	90	90	90
Volume [Å^3]	4804.7(3)	4795.1(3)	4773.6(3)	4776.7(3)	4778.2(3)	4780.3(3)	4790.6(3)	4788.7(3)
Z	4	4	4	4	4	4	4	4
ρ_{calc} [g cm^{-3}]	1.205	1.207	1.212	1.212	1.211	1.211	1.208	1.209
μ [mm^{-1}]	0.201	0.201	0.202	0.202	0.202	0.202	0.201	0.201
F(000)	1856.0	1856.0	1856.0	1856.0	1856.0	1856.0	1856.0	1856.0
Crystal size [mm^3]	0.1 × 0.1 × 0.05	0.1 × 0.1 × 0.05	0.1 × 0.1 × 0.05	0.1 × 0.1 × 0.05	0.1 × 0.1 × 0.05	0.1 × 0.1 × 0.05	0.1 × 0.1 × 0.05	0.1 × 0.1 × 0.05
Wavelength λ [Å]	0.534	0.534	0.534	0.534	0.534	0.534	0.534	0.534
2θ range [°]	2.466 to 38.992	2.468 to 38.988	2.472 to 38.994	2.756 to 38.992	2.47 to 38.99	2.472 to 38.992	2.758 to 38.992	2.47 to 38.99
Reflections collected	32734	32765	32772	32764	32550	32782	32888	32923
Independent reflections	9775 [R_{int} = 0.0893, R_{sigma} = 0.1109]	9761 [R_{int} = 0.0978, R_{sigma} = 0.1092]	9681 [R_{int} = 0.1002, R_{sigma} = 0.1226]	9676 [R_{int} = 0.1101, R_{sigma} = 0.1216]	9741 [R_{int} = 0.1016, R_{sigma} = 0.1387]	9738 [R_{int} = 0.1043, R_{sigma} = 0.1337]	9603 [R_{int} = 0.0760, R_{sigma} = 0.0789]	9599 [R_{int} = 0.0865, R_{sigma} = 0.0851]
Goodness of Fit on F^2	0.923	0.932	0.872	0.884	0.879	0.905	0.951	0.964
Final R indices $I \geq 2\sigma(I)$	R_1 = 0.0551, wR_2 = 0.1142	R_1 = 0.0569, wR_2 = 0.1211	R_1 = 0.0605, wR_2 = 0.1320	R_1 = 0.0611, wR_2 = 0.1269	R_1 = 0.0606, wR_2 = 0.1200	R_1 = 0.0596, wR_2 = 0.1177	R_1 = 0.0539, wR_2 = 0.1207	R_1 = 0.0552, wR_2 = 0.1263
Final R indices all data	R_1 = 0.1022, wR_2 = 0.1290	R_1 = 0.0956, wR_2 = 0.1365	R_1 = 0.1254, wR_2 = 0.1587	R_1 = 0.1120, wR_2 = 0.1495	R_1 = 0.1348, wR_2 = 0.1436	R_1 = 0.1199, wR_2 = 0.1376	R_1 = 0.0946, wR_2 = 0.1381	R_1 = 0.0922, wR_2 = 0.1452
Largest diff. peak/hole	0.33/-0.38	0.44/-0.42	0.48/-0.56	0.45/-0.54	0.42/-0.47	0.45/-0.41	0.43/-0.55	0.42/-0.60

Table S17 *cont.* Single-crystal X-ray data for selected pump-multiprobe data collections. Crystal data for the structures with the highest and lowest excited-state population α in each pump-multiprobe experiment are given. The average level of X-ray induced excitation across the experiments was ~ 4 %; where the lowest measured α in each pair of entries are significantly above this level this implies the excited state had not fully decayed during the selected t_{cyc}.

t_{acq} [s]	0.8	0.8	0.4	0.4	0.4	0.4	0.4	0.4
**Repeat**	**1**	**1**	**1**	**1**	**2**	**2**	**3**	**3**
***T* [K]**	**280**	**280**	**282**	**282**	**283**	**283**	**284**	**284**
Experiment	280K_0.8s_t4_excit	280K_0.8s_t11_decay	282K_0.4s_t3_excit	282K_0.4s_t9_decay	283K_0.4s_t3_excit	283K_0.4s_t9_decay	284K_0.4s_t3_excit	284K_0.4s_t9_decay
t_{exc} [s]	8	8	5	5	5	5	5	5
ES conversion, α [%]	11.8	4.4	8.7	3.6	10.4	3.4	8.0	3.8
Empirical formula	$C_{48}H_{73}BN_4O_3Pd$	$C_{48}H_{73}BN_4O_3Pd$	$C_{48}H_{73}BN_4O_3Pd$	$C_{48}H_{73}BN_4O_3Pd$	$C_{48}H_{73}BN_4O_3Pd$	$C_{48}H_{73}BN_4O_3Pd$	$C_{48}H_{73}BN_4O_3Pd$	$C_{48}H_{73}BN_4O_3Pd$
Formula weight [g mol^{-1}]	871.31	871.31	871.31	871.31	871.31	871.31	871.31	871.31
Crystal system	monoclinic	monoclinic	monoclinic	monoclinic	monoclinic	monoclinic	monoclinic	monoclinic
Space group	$P2_1/n$	$P2_1/n$	$P2_1/n$	$P2_1/n$	$P2_1/n$	$P2_1/n$	$P2_1/n$	$P2_1/n$
a [Å]	11.6799(4)	11.6820(4)	11.6826(7)	11.6899(7)	11.6995(5)	11.6997(5)	11.6885(5)	11.6922(5)
b [Å]	13.5938(5)	13.5896(6)	13.6088(7)	13.6071(8)	13.6308(5)	13.6253(6)	13.6052(7)	13.6069(7)
c [Å]	30.2110(10)	30.1948(11)	30.2346(15)	30.2249(16)	30.2963(12)	30.2831(13)	30.1939(12)	30.1903(12)
α [°]	90	90	90	90	90	90	90	90
β [°]	93.319(3)	93.408(3)	93.228(5)	93.276(5)	93.208(4)	93.260(4)	93.246(4)	93.272(4)
γ [°]	90	90	90	90	90	90	90	90
Volume [Å^3]	4788.7(3)	4785.1(3)	4799.3(4)	4799.9(5)	4823.9(3)	4819.7(4)	4793.9(4)	4795.3(4)
Z	4	4	4	4	4	4	4	4
ρ_{calc} [g cm^{-3}]	1.209	1.209	1.206	1.206	1.200	1.201	1.207	1.207
μ [mm^{-1}]	0.201	0.201	0.201	0.201	0.200	0.200	0.201	0.201
F(000)	1856.0	1856.0	1856.0	1856.0	1856.0	1856.0	1856.0	1856.0
Crystal size [mm^3]	0.1 × 0.1 × 0.05	0.1 × 0.1 × 0.05	0.1 × 0.1 × 0.05	0.1 × 0.1 × 0.05	0.1 × 0.1 × 0.05	0.1 × 0.1 × 0.05	0.1 × 0.1 × 0.05	0.1 × 0.1 × 0.05
Wavelength λ [Å]	0.534	0.534	0.534	0.534	0.534	0.534	0.534	0.534
2θ range [°]	2.758 to 38.99	2.756 to 38.994	2.466 to 38.99	2.466 to 38.992	2.462 to 38.99	2.754 to 38.992	2.468 to 38.992	2.756 to 38.992
Reflections collected	27786	27789	32702	32735	32844	32834	32732	32753
Independent reflections	9400 [R_{int} = 0.0797, R_{sigma} = 0.1039]	9394 [R_{int} = 0.0937, R_{sigma} = 0.1113]	9721 [R_{int} = 0.0910, R_{sigma} = 0.1072]	9726 [R_{int} = 0.0974, R_{sigma} = 0.1075]	9783 [R_{int} = 0.0906, R_{sigma} = 0.1059]	9778 [R_{int} = 0.0912, R_{sigma} = 0.1017]	9616 [R_{int} = 0.0916, R_{sigma} = 0.0917]	9615 [R_{int} = 0.0968, R_{sigma} = 0.0948]
Goodness of Fit on F^2	0.923	0.940	0.876	0.881	0.884	0.886	0.965	0.962
Final R indices $I \geq 2\sigma(I)$	R_1 = 0.0584, wR_2 = 0.1167	R_1 = 0.0623, wR_2 = 0.1283	R_1 = 0.0567, wR_2 = 0.1152	R_1 = 0.0573, wR_2 = 0.1181	R_1 = 0.0609, wR_2 = 0.1345	R_1 = 0.0599, wR_2 = 0.1310	R_1 = 0.0628, wR_2 = 0.1415	R_1 = 0.0627, wR_2 = 0.1412
Final R indices all data	R_1 = 0.1096, wR_2 = 0.1350	R_1 = 0.1087, wR_2 = 0.1503	R_1 = 0.1158, wR_2 = 0.1338	R_1 = 0.1126, wR_2 = 0.1383	R_1 = 0.1305, wR_2 = 0.1637	R_1 = 0.1209, wR_2 = 0.1567	R_1 = 0.1129, wR_2 = 0.1709	R_1 = 0.1120, wR_2 = 0.1703
Largest diff. peak/hole	0.36/-0.46	0.46/-0.49	0.34/-0.45	0.38/-0.39	0.60/-0.62	0.52/-0.48	0.44/-0.62	0.39/-0.55

Supplementary Note 6: Pump-multiprobe data fitting

The pump-multiprobe datasets were analysed by fitting the excited-state populations as a function of time, $\alpha(t)$, using numerical simulations based on a two-process JMAK model as described in Section 4 above. We also account for the small background excitation α_{bg} as a fit parameter. For each dataset, we first estimate a decay rate constant k_{dec} based on the Arrhenius parameterisation in Table S14, together with an α_{bg} from the data, and use the excitation data ($t \leq t_{\text{exc}}$) to fit an approximate excitation rate k_{exc}. We then refine all three parameters freely against the complete $\alpha(t)$. This allows us to determine for each experiment an excitation and decay rate and a background excitation α_{bg}. (The Avrami exponents for the excitation and decay are both assumed to be unity.) The fit parameters for each of the experiments carried out in this work are listed in Table S18.

This fit also allows us to determine a maximum excitation level at $t = t_{\text{exc}}$ after accounting for any background excitation. It is of interest to compare this to the theoretical steady-state excitation level α_{SS} that could be achieved with the fitted kinetic parameters - this comparison is shown in Table S19.

Table S18 Kinetic parameters obtained by fitting the excited-state populations as a function of $\alpha(t)$ measured in each of the pump-multiprobe experiments on **1** carried out in this work: α_{bg} - background ES population; k_{exc} = excitation rate constant; k_{dec} - decay rate constant; RMS - root-mean-square error on the fit. The data fits are shown in Figures S17-S28.

t_{cyc} [s]	t_{exc} [s]	t_{dec} [s]	T [K]	α_{bg} [%]	k_{exc} [s^{-1}]	k_{dec} [s^{-1}]	RMS [%]
170	55	115	260	5.98	4.63×10^{-3}	1.43×10^{-2}	0.43
170	55	115	265	3.25	1.57×10^{-2}	2.74×10^{-2}	0.45
170	55	115	270	3.71	8.34×10^{-3}	4.94×10^{-2}	0.48
108	35	73	265	4.80	8.79×10^{-3}	2.65×10^{-2}	0.57
108	35	73	270	2.30	1.19×10^{-2}	4.70×10^{-2}	0.35
108	35	73	272	3.97	1.56×10^{-2}	6.44×10^{-2}	0.62
108	35	73	274	3.84	1.78×10^{-2}	7.86×10^{-2}	0.43
35	14	21	280	2.95	2.12×10^{-2}	2.38×10^{-1}	0.30
22	8	14	280	2.73	2.38×10^{-2}	1.83×10^{-1}	0.44
14	5	9	282	1.31	2.17×10^{-2}	1.77×10^{-1}	0.41
14	5	9	283	0.45	3.24×10^{-2}	1.68×10^{-1}	0.39
14	5	9	284	2.71	1.94×10^{-2}	2.42×10^{-1}	0.57

Table S19 Maximum excited-state population α_{max} obtained in each of the pump-multiprobe experiments on **1** listed in Table S16 based on the data fits shown in Figures S17-S28. Also shown are the predicted maximum steady-state populations attainable under continuous illumination using the same kinetic parameters, α_{SS}, and the ratios $\alpha_{\text{max}}/\alpha_{\text{SS}}$ as a percentage. Note that here α_{max} refers to the maximum conversion determined after data fitting, and may differ from the largest measured ES populations ($\alpha(t = t_{exc})$ in Tables S16 / S17) due mainly to the subtraction of the fitted background ES population α_{bg}.

t_{cyc} [s]	t_{exc} [s]	t_{dec} [s]	T [K]	α_{max} [%]	α_{SS} [%]	Ratio [%]
170	55	115	260	17.0	24.0	70.8
170	55	115	265	33.2	36.2	91.7
170	55	115	270	13.8	14.3	96.5
108	35	73	265	18.4	24.6	74.8
108	35	73	270	17.7	20.0	88.5
108	35	73	272	18.3	19.3	94.8
108	35	73	274	17.8	18.4	96.7
35	14	21	280	8.00	8.20	97.6
22	8	14	280	9.40	11.5	81.7
14	5	9	282	7.40	10.9	67.9
14	5	9	283	11.1	16.1	68.9
14	5	9	284	5.60	7.40	75.7

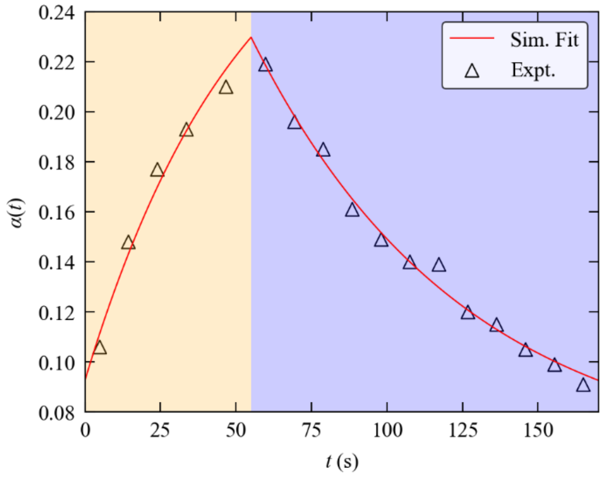

Figure S17 Data fitting for the pump-multiprobe experiment with **1** using the parameters t_{cyc} = 170 s, t_{exc} = 55 s, t_{dec} = 115 s and T = 260 K. The markers show the ES population as a function of time $\alpha(t)$ obtained by solving and refining single-crystal structures from the pump-multiprobe diffraction datasets. The solid line shows the $\alpha(t)$ predicted by numerical simulations using a two-process JMAK model with the fitted kinetic parameters listed in Table S18. The shaded yellow and blue regions of the plot mark the excitation and decay phases of the pump-multiprobe cycle, respectively.

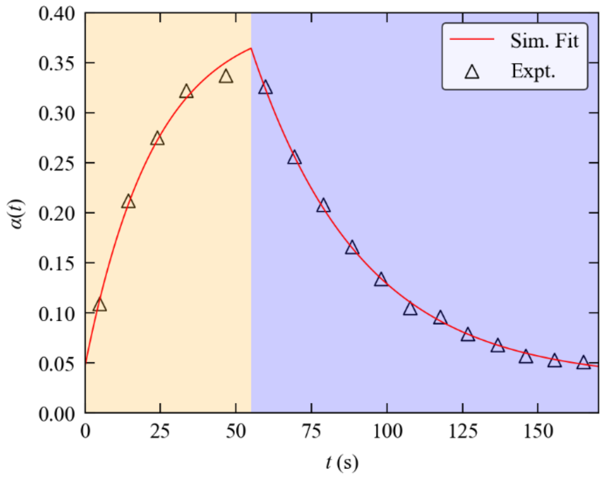

Figure S18 Data fitting for the pump-multiprobe experiment with **1** using the parameters t_{cyc} = 170 s, t_{exc} = 55 s, t_{dec} = 115 s and T = 265 K. The markers show the ES population as a function of time $\alpha(t)$ obtained by solving and refining single-crystal structures from the pump-multiprobe diffraction datasets. The solid line shows the $\alpha(t)$ predicted by numerical simulations using a two-process JMAK model with the fitted kinetic parameters listed in Table S18. The shaded yellow and blue regions of the plot mark the excitation and decay phases of the pump-multiprobe cycle, respectively.

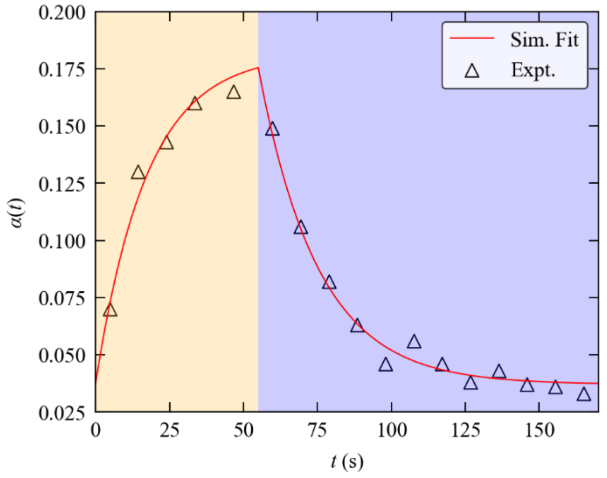

Figure S19 Data fitting for the pump-multiprobe experiment with **1** using the parameters t_{cyc} = 170 s, t_{exc} = 55 s, t_{dec} = 115 s and T = 270 K. The markers show the ES population as a function of time $\alpha(t)$ obtained by solving and refining single-crystal structures from the pump-multiprobe diffraction datasets. The solid line shows the $\alpha(t)$ predicted by numerical simulations using a two-process JMAK model with the fitted kinetic parameters listed in Table S18. The shaded yellow and blue regions of the plot mark the excitation and decay phases of the pump-multiprobe cycle, respectively.

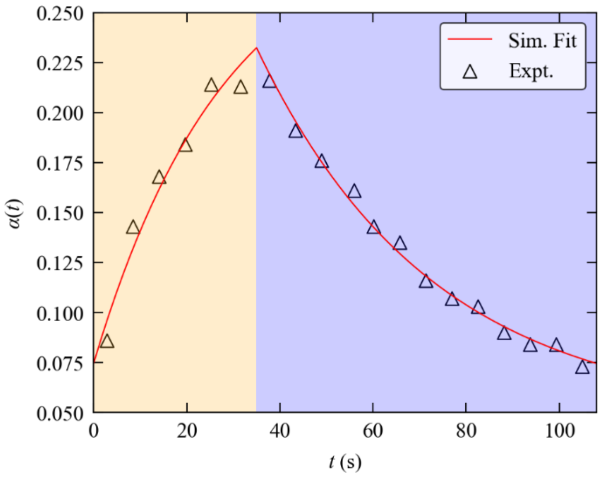

Figure S20 Data fitting for the pump-multiprobe experiment with **1** using the parameters t_{cyc} = 108 s, t_{exc} = 35 s, t_{dec} = 73 s and T = 265 K. The markers show the ES population as a function of time $\alpha(t)$ obtained by solving and refining single-crystal structures from the pump-multiprobe diffraction datasets. The solid line shows the $\alpha(t)$ predicted by numerical simulations using a two-process JMAK model with the fitted kinetic parameters listed in Table S18. The shaded yellow and blue regions of the plot mark the excitation and decay phases of the pump-multiprobe cycle, respectively.

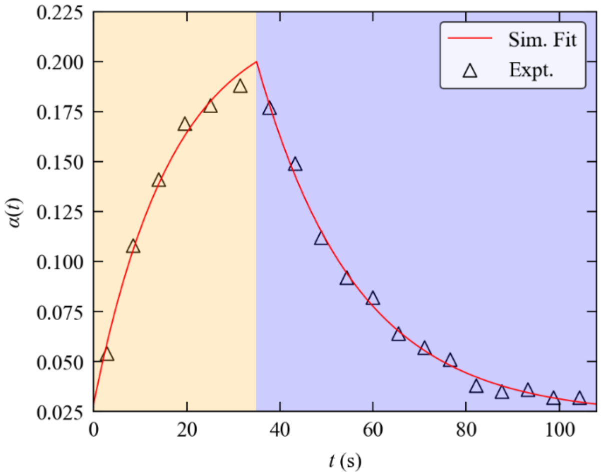

Figure S21 Data fitting for the pump-multiprobe experiment with **1** using the parameters t_{cyc} = 108 s, t_{exc} = 35 s, t_{dec} = 73 s and T = 270 K. The markers show the ES population as a function of time $\alpha(t)$ obtained by solving and refining single-crystal structures from the pump-multiprobe diffraction datasets. The solid line shows the $\alpha(t)$ predicted by numerical simulations using a two-process JMAK model with the fitted kinetic parameters listed in Table S18. The shaded yellow and blue regions of the plot mark the excitation and decay phases of the pump-multiprobe cycle, respectively.

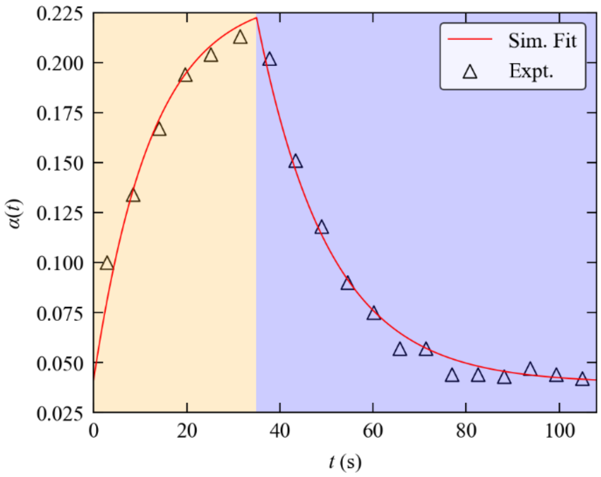

Figure S22 Data fitting for the pump-multiprobe experiment with **1** using the parameters t_{cyc} = 108 s, t_{exc} = 35 s, t_{dec} = 73 s and T = 272 K. The markers show the ES population as a function of time $\alpha(t)$ obtained by solving and refining single-crystal structures from the pump-multiprobe diffraction datasets. The solid line shows the $\alpha(t)$ predicted by numerical simulations using a two-process JMAK model with the fitted kinetic parameters listed in Table S18. The shaded yellow and blue regions of the plot mark the excitation and decay phases of the pump-multiprobe cycle, respectively.

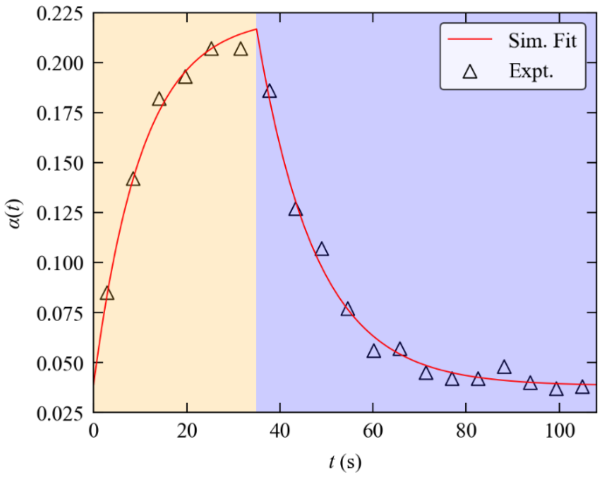

Figure S23 Data fitting for the pump-multiprobe experiment with **1** using the parameters t_{cyc} = 108 s, t_{exc} = 35 s, t_{dec} = 73 s and T = 274 K. The markers show the ES population as a function of time $\alpha(t)$ obtained by solving and refining single-crystal structures from the pump-multiprobe diffraction datasets. The solid line shows the $\alpha(t)$ predicted by numerical simulations using a two-process JMAK model with the fitted kinetic parameters listed in Table S18. The shaded yellow and blue regions of the plot mark the excitation and decay phases of the pump-multiprobe cycle, respectively.

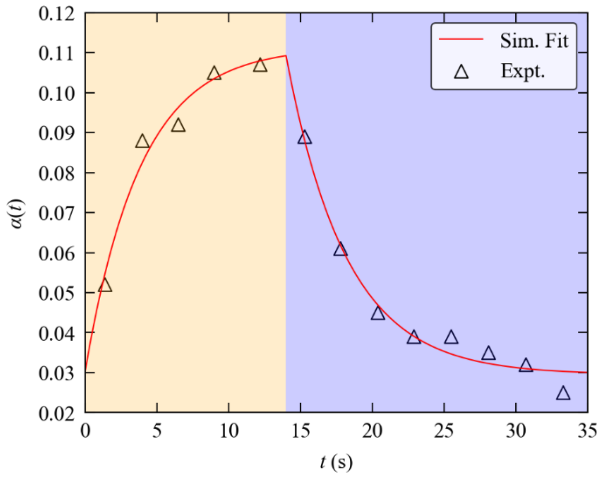

Figure S24 Data fitting for the pump-multiprobe experiment with **1** using the parameters t_{cyc} = 35 s, t_{exc} = 14 s, t_{dec} = 21 s and T = 280 K. The markers show the ES population as a function of time $\alpha(t)$ obtained by solving and refining single-crystal structures from the pump-multiprobe diffraction datasets. The solid line shows the $\alpha(t)$ predicted by numerical simulations using a two-process JMAK model with the fitted kinetic parameters listed in Table S18. The shaded yellow and blue regions of the plot mark the excitation and decay phases of the pump-multiprobe cycle, respectively.

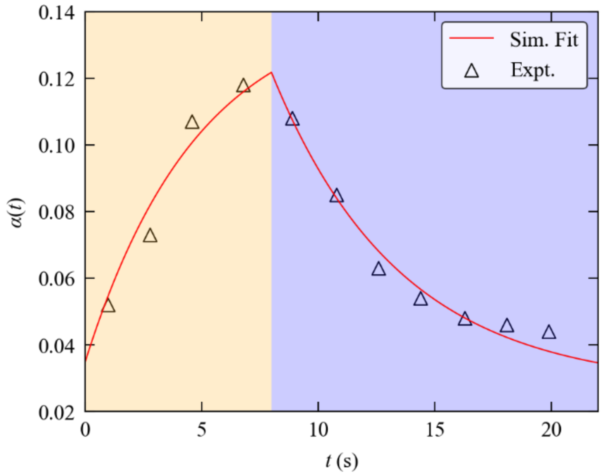

Figure S25 Data fitting for the pump-multiprobe experiment with **1** using the parameters t_{cyc} = 22 s, t_{exc} = 8 s, t_{dec} = 14 s and T = 280 K. The markers show the ES population as a function of time $\alpha(t)$ obtained by solving and refining single-crystal structures from the pump-multiprobe diffraction datasets. The solid line shows the $\alpha(t)$ predicted by numerical simulations using a two-process JMAK model with the fitted kinetic parameters listed in Table S18. The shaded yellow and blue regions of the plot mark the excitation and decay phases of the pump-multiprobe cycle, respectively.

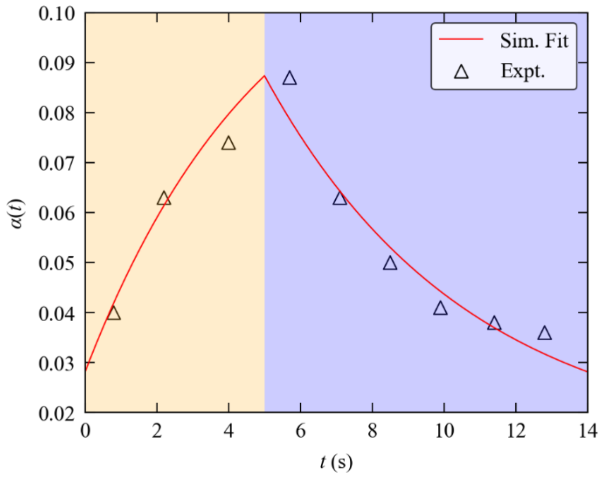

Figure S26 Data fitting for the pump-multiprobe experiment with **1** using the parameters t_{cyc} = 14 s, t_{exc} = 5 s, t_{dec} = 9 s and T = 282 K. The markers show the ES population as a function of time $\alpha(t)$ obtained by solving and refining single-crystal structures from the pump-multiprobe diffraction datasets. The solid line shows the $\alpha(t)$ predicted by numerical simulations using a two-process JMAK model with the fitted kinetic parameters listed in Table S18. The shaded yellow and blue regions of the plot mark the excitation and decay phases of the pump-multiprobe cycle, respectively.

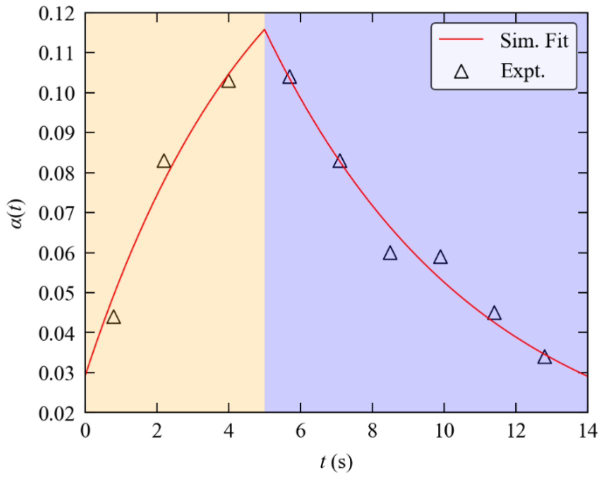

Figure S27 Data fitting for the pump-multiprobe experiment with **1** using the parameters t_{cyc} = 14 s, t_{exc} = 5 s, t_{dec} = 9 s and T = 283 K. The markers show the ES population as a function of time $\alpha(t)$ obtained by solving and refining single-crystal structures from the pump-multiprobe diffraction datasets. The solid line shows the $\alpha(t)$ predicted by numerical simulations using a two-process JMAK model with the fitted kinetic parameters listed in Table S18. The shaded yellow and blue regions of the plot mark the excitation and decay phases of the pump-multiprobe cycle, respectively.

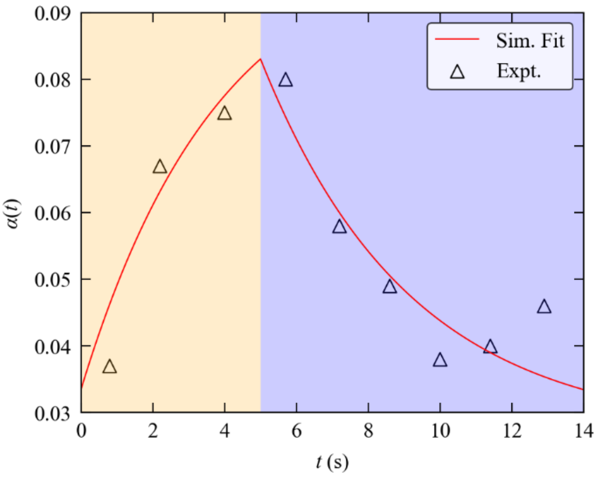

Figure S28 Data fitting for the pump-multiprobe experiment with **1** using the parameters t_{cyc} = 14 s, t_{exc} = 5 s, t_{dec} = 9 s and T = 284 K. The markers show the ES population as a function of time $\alpha(t)$ obtained by solving and refining single-crystal structures from the pump-multiprobe diffraction datasets. The solid line shows the $\alpha(t)$ predicted by numerical simulations using a two-process JMAK model with the fitted kinetic parameters listed in Table S18. The shaded yellow and blue regions of the plot mark the excitation and decay phases of the pump-multiprobe cycle, respectively.

Supplementary Note 7: Time-resolved molecular movies

Molecular movies generated for the pump-multiprobe experiments with the largest maximum excited-state populations, under each of the timing regimes tested, are provided as follows.

- *8s_Animation.gif* - molecular movie for the experiment at t_{acq} = 8 s
- *4s_Animation.gif* - molecular movie for the experiment at t_{acq} = 4 s
- *1-6s_Animation.gif* - molecular movie for the experiment at t_{acq} = 1.6 s
- *0-8s_Animation.gif* - molecular movie for the experiment at t_{acq} = 0.8 s
- *0-4s_Animation.gif* - molecular movie for the experiment at t_{acq} = 0.4 s

Supplementary Note 8: "Normal" Wilson Plots for all pump-multiprobe datasets collected at the lowest (260 K) and highest (284 K) experiment temperatures

Dataset: 260K_8s_t1_excit

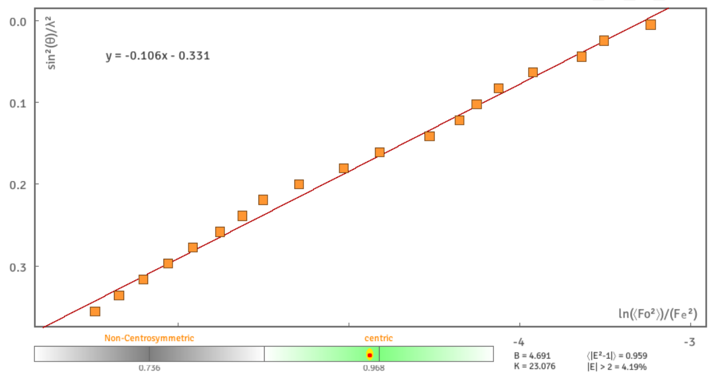

Dataset: 260K_8s_t2_excit

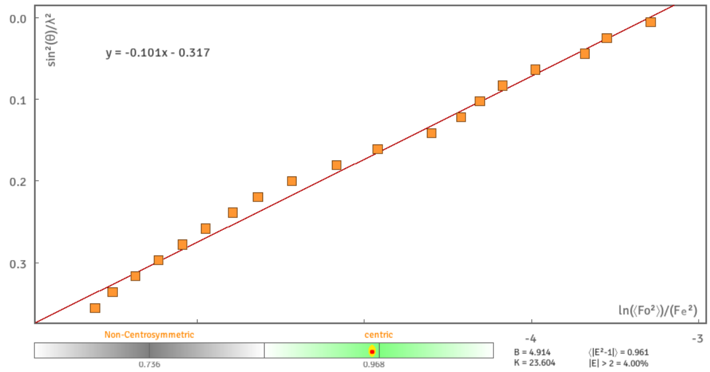

Dataset: 260K_8s_t3_excit

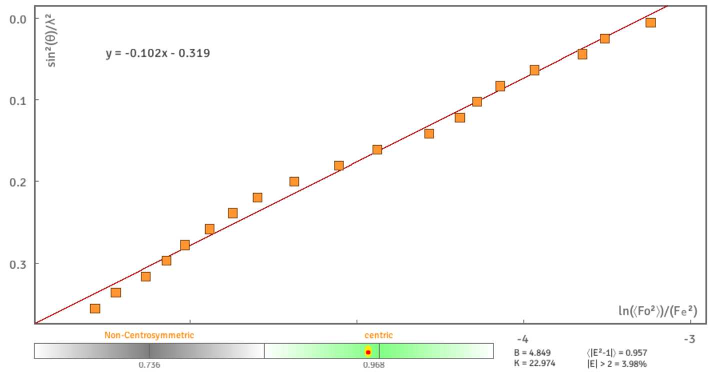

Dataset: 260K_8s_t4_excit

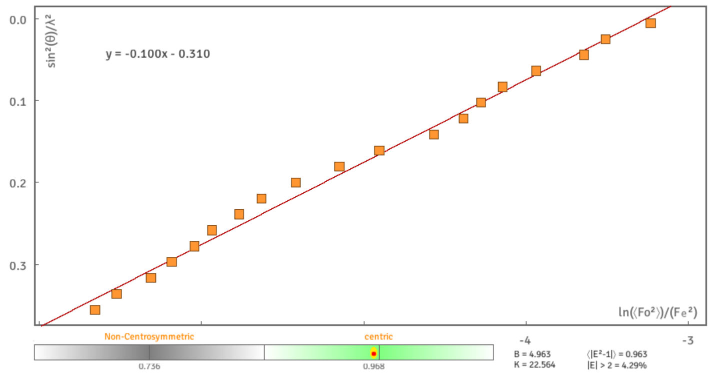

Dataset: 260K_8s_t5_excit

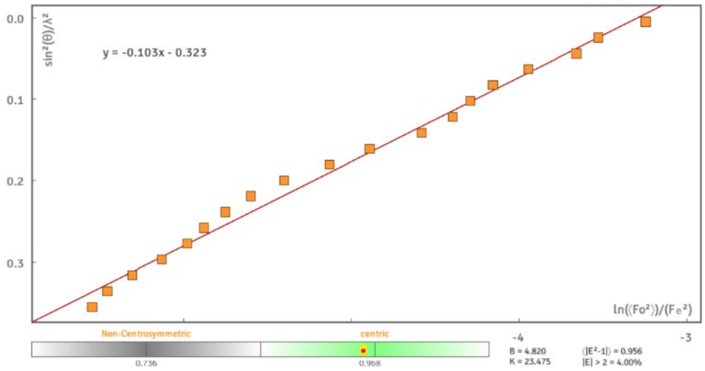

Dataset: 260K_8s_t6_decay

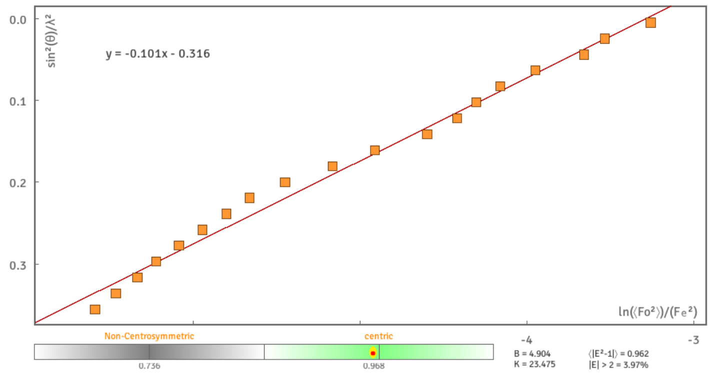

Dataset: 260K_8s_t7_decay

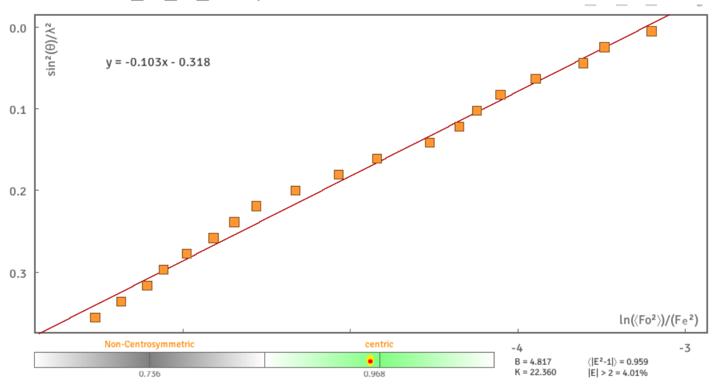

Dataset: 260K_8s_t8_decay

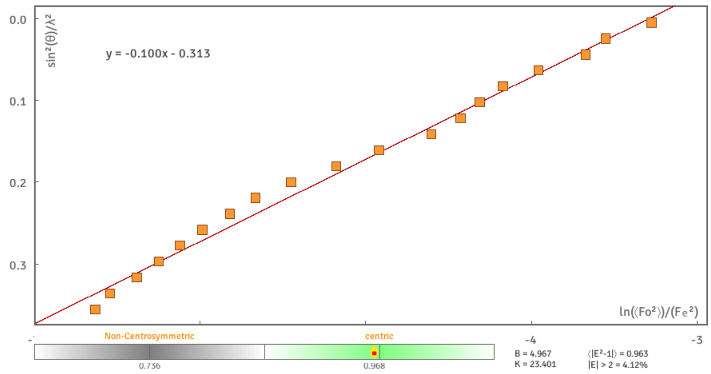

Dataset: 260K_8s_t9_decay

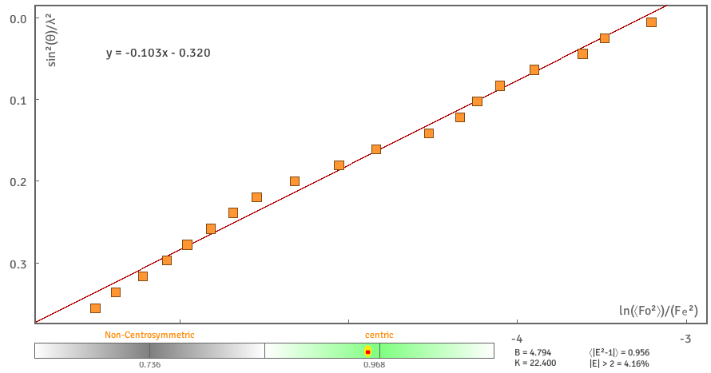

Dataset: 260K_8s_t10_decay

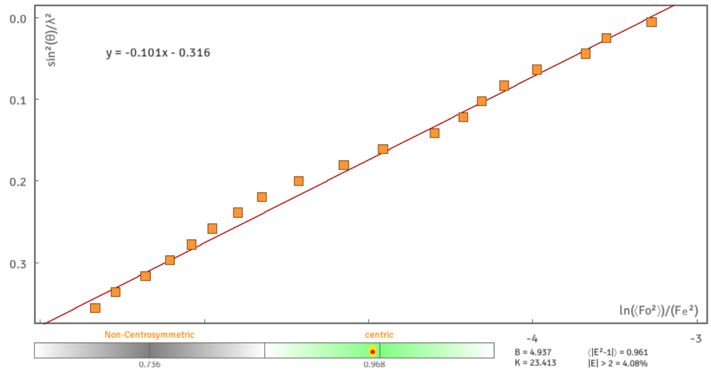

Dataset: 260K_8s_t11_decay

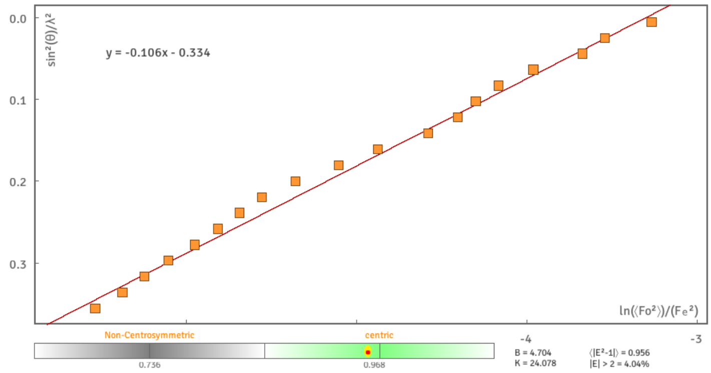

Dataset: 260K_8s_t12_decay

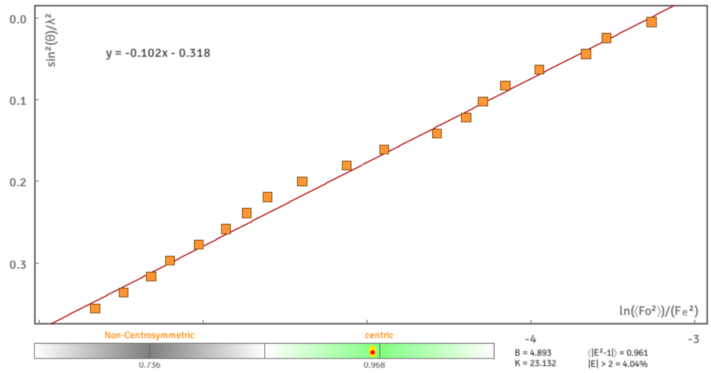

Dataset: 260K_8s_t13_decay

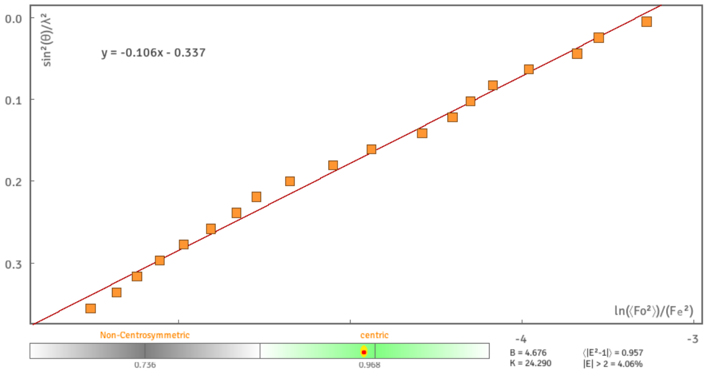

Dataset: 260K_8s_t14_decay

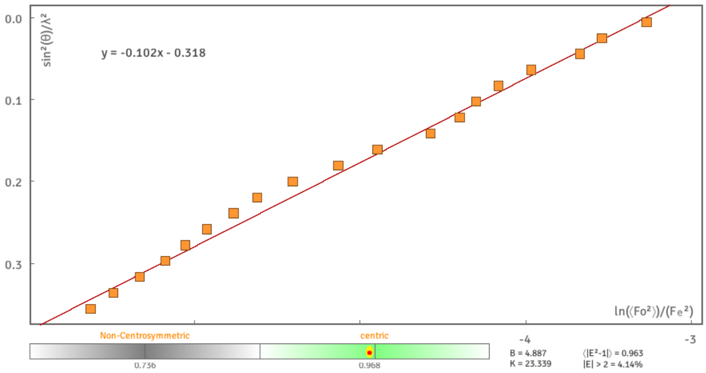

Dataset: 260K_8s_t15_decay

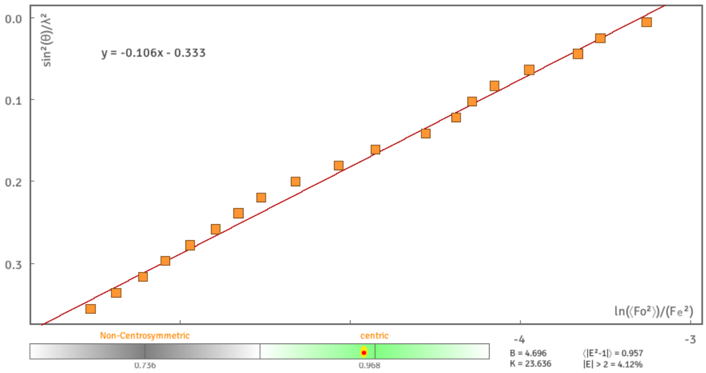

Dataset: 260K_8s_t16_decay

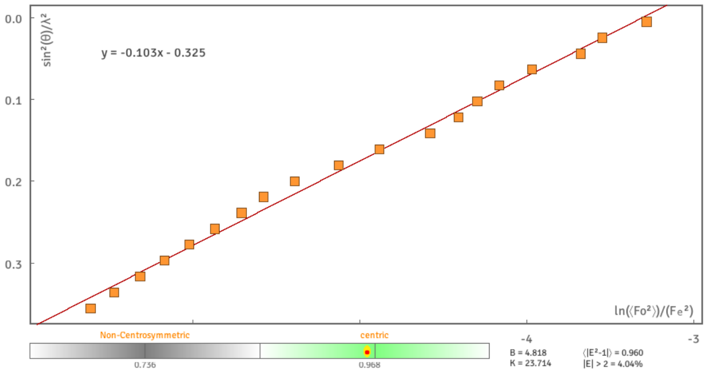

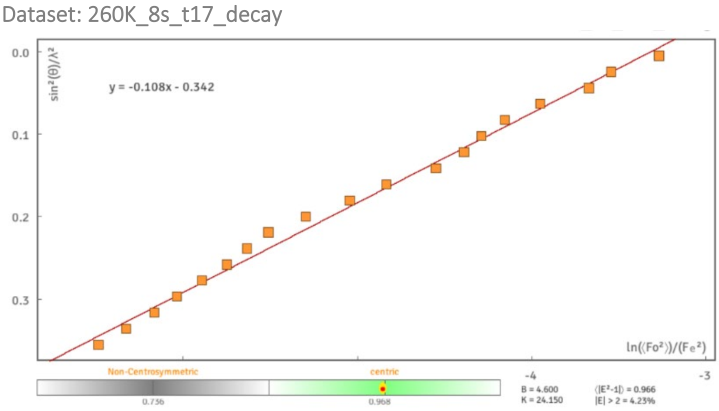

Figure S29 Wilson Plots for all delay time-points collected in the pump-multiprobe experiment at 260 K (t_{acq} = 8 s). The strong linear fit for all plots indicates that there is no significant deterioration of the crystal during the process and the consistency across the different time delays proves that the pump-multiprobe method is successful at ensuring the measurements at different time-delays are not unequally affected by cumulative problems such as crystal damage, photobleaching or sample heating.

Dataset: 284K_0.4s_t1_excit

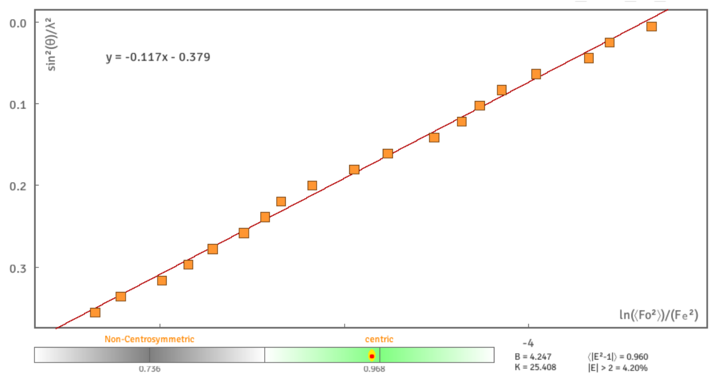

Dataset: 284K_0.4s_t2_excit

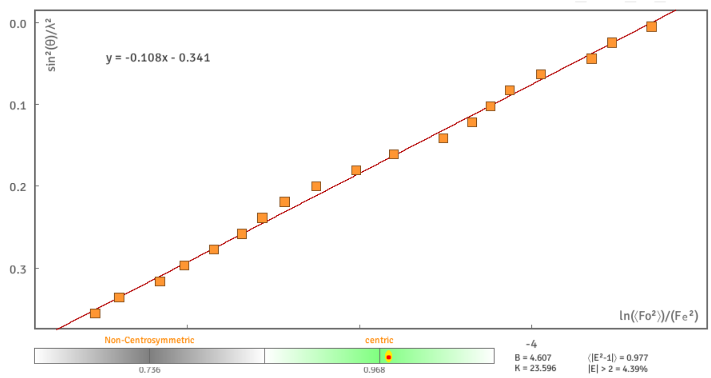

Dataset: 284K_0.4s_t3_excit

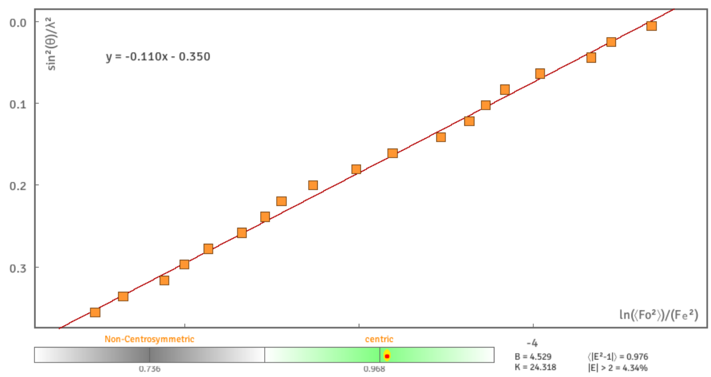

Dataset: 284K_0.4s_t4_decay

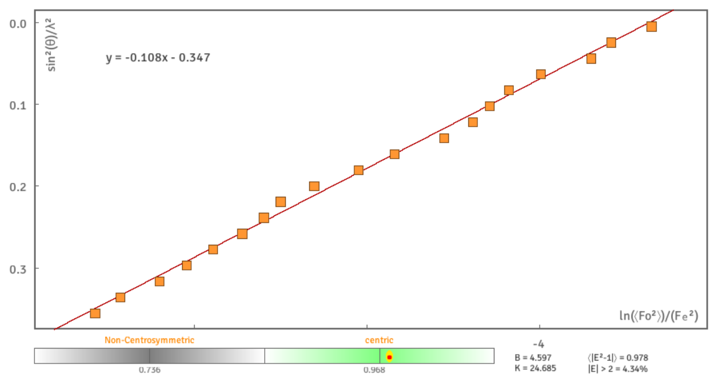

Dataset: 284K_0.4s_t5_decay

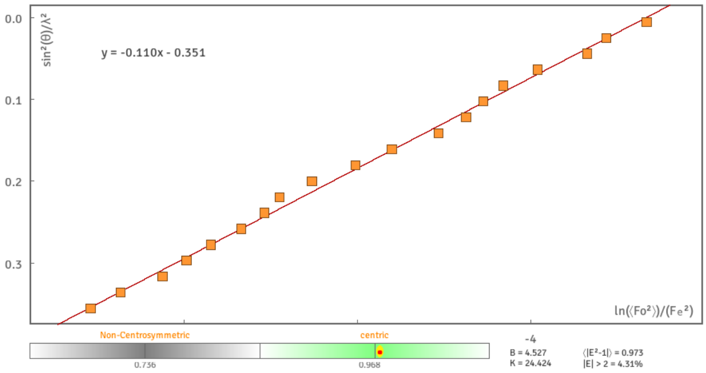

Dataset: 284K_0.4s_t6_decay

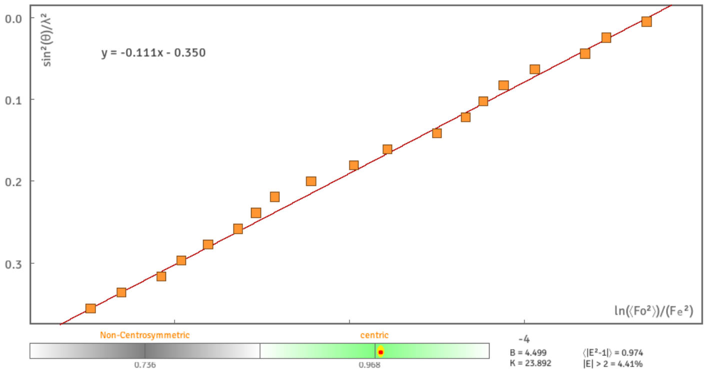

Dataset: 284K_0.4s_t7_decay

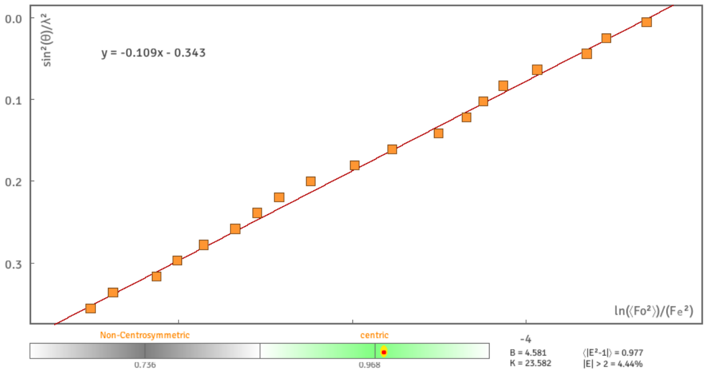

Dataset: 284K_0.4s_t8_decay

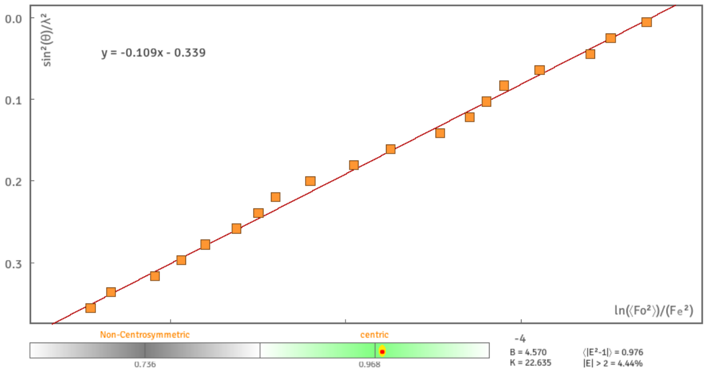

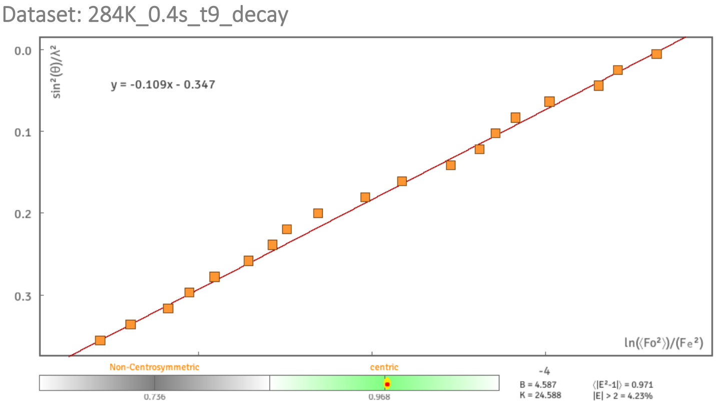

Figure S30 Wilson Plots for all delay time-points collected in the pump-multiprobe experiment at 284 K (t_{acq} = 0.4 s). The strong linear fit for all plots indicates that there is no significant deterioration of the crystal during the process and the consistency across the different time delays proves that the pump-multiprobe method is successful at ensuring the measurements at different time-delays are not unequally affected by cumulative problems such as crystal damage, photobleaching or sample heating.

Supplementary Note 9: "Photo-Wilson" Plots for all pump-multiprobe datasets

Photo-Wilson plots were constructed following the methods developed by Coppens *et al.*[7] The plots allow determination of a temperature scale factor, k_B, that can be used to assess the degree of sample heating as a result to cumulative pumping by the illumination set-up. A value of k_B = 1.00 indicates no temperature scaling is needed and there is no increase in sample temperature as a result of irradiation.

Plots were created for all 12 pump-multiprobe datasets collected in this study and are included in Figure S31 below.

An average (mean) value of k_B = 1.069 ± 0.028 across all 12 datasets, with a maximum k_B = 1.187 ± 0.009 and a minimum of k_B = 0.987 ± 0.007 (i.e. a range of 0.200). Comparing these values with the literature indicates that the temperature increase as a result of the cumulative pumping with the LED sphere for several seconds is small, if not negligible, when considering the errors.[7]

Dataset: 260K_8s (t_{acq} = 8 s, t_{exc} = 55 s):

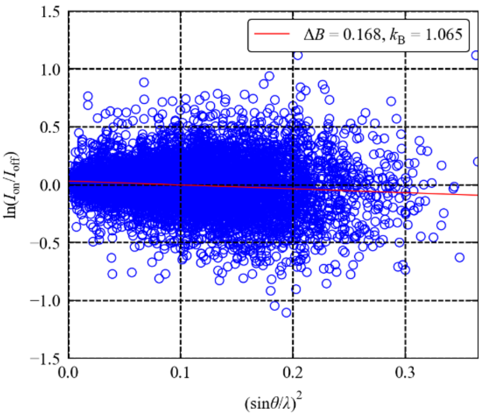

Dataset: 265K_8s (t_{acq} = 8 s, t_{exc} = 55 s):

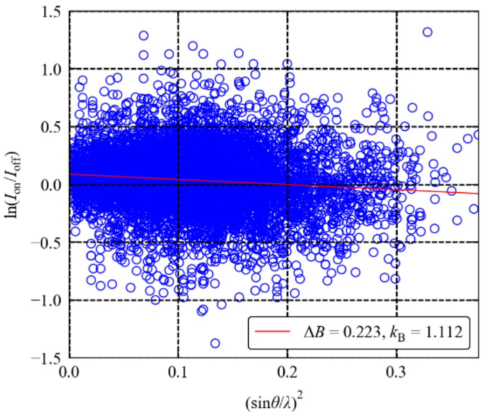

Dataset: 270K_8s (t_{acq} = 8 s, t_{exc} = 55 s):

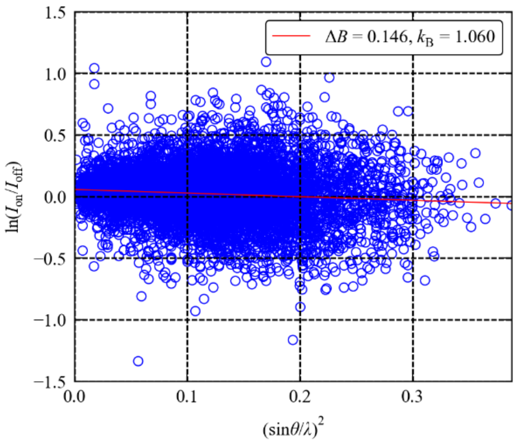

Dataset: 265K_4s (t_{acq} = 4 s, t_{exc} = 35 s):

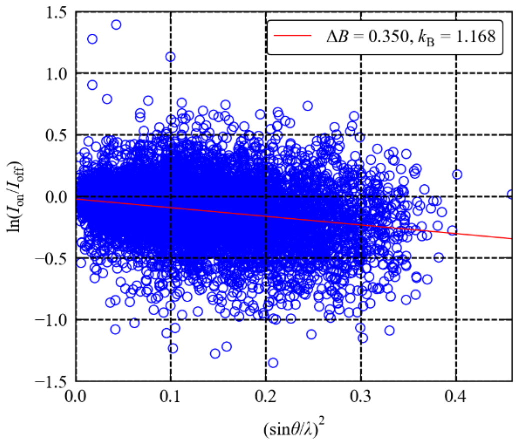

Dataset: 270K_4s (t_{acq} = 4 s, t_{exc} = 35 s):

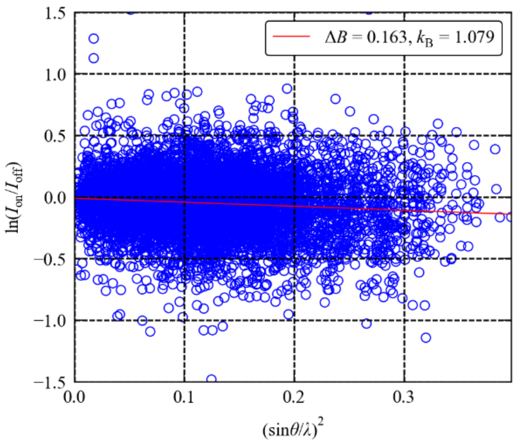

Dataset: 272K_4s (t_{acq} = 4 s, t_{exc} = 35 s):

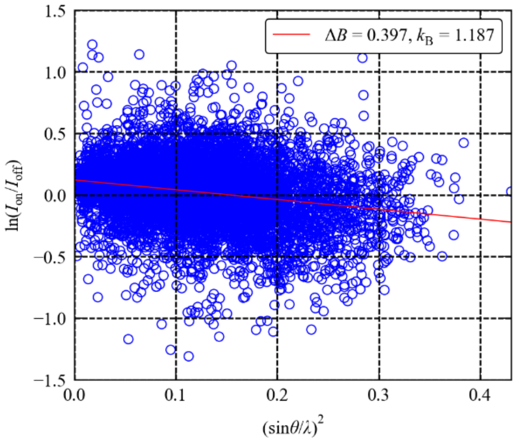

Dataset: 274K_4s (t_{acq} = 4 s, t_{exc} = 35 s):

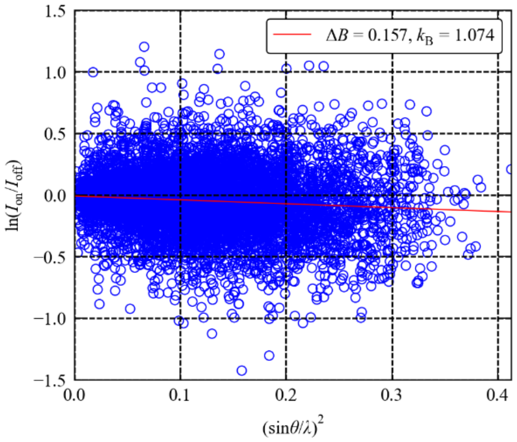

Dataset: 280K_1.6s (t_{acq} = 1.6 s, t_{exc} = 14 s):

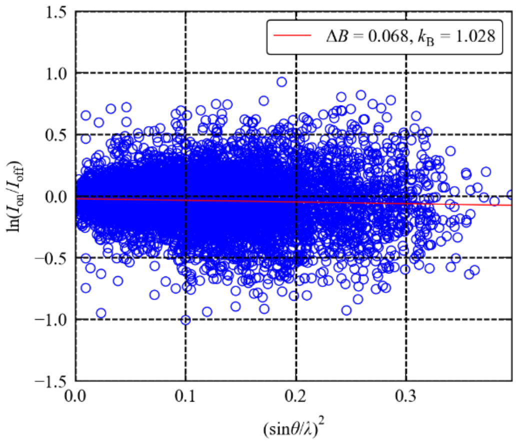

Dataset: 280K_0.8s (t_{acq} = 0.8 s, t_{exc} = 8 s):

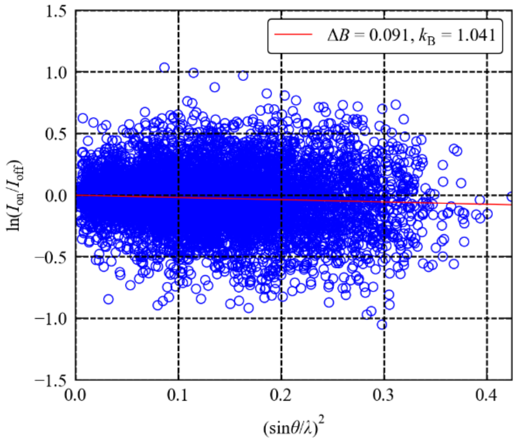

Dataset: 282K_0.4s (t_{acq} = 0.4 s, t_{exc} = 5 s):

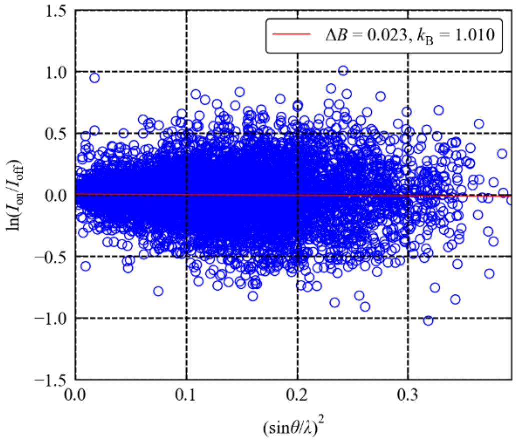

Dataset: 283K_0.4s (t_{acq} = 0.4 s, t_{exc} = 5 s):

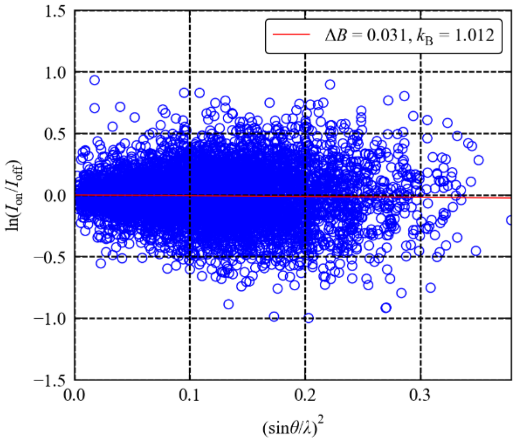

Dataset: 284K_0.4s (t_{acq} = 0.4 s, t_{exc} = 5 s):

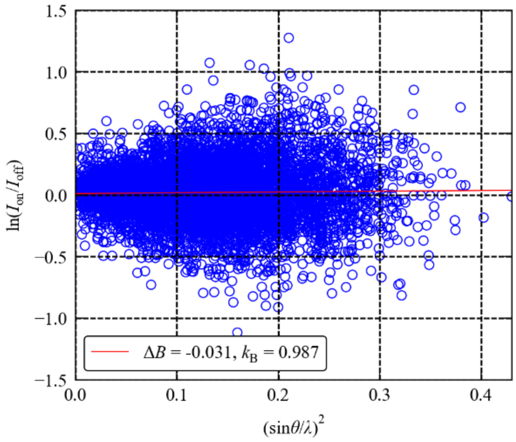

Figure S31 Photo-Wilson plots constructed for all 12 pump-multiprobe experiments with the LED sphere set-up. ΔB values are obtained from the slope of the plot and k_B values determined following the methods of Coppens et al.[7] All k_B values are close to 1.00, indicating that the temperature change at the sample of a result of cumulative pumping with the LED sphere is close to negligible.

Supplementary Note 10. Nudged-elastic band (NEB) calculations

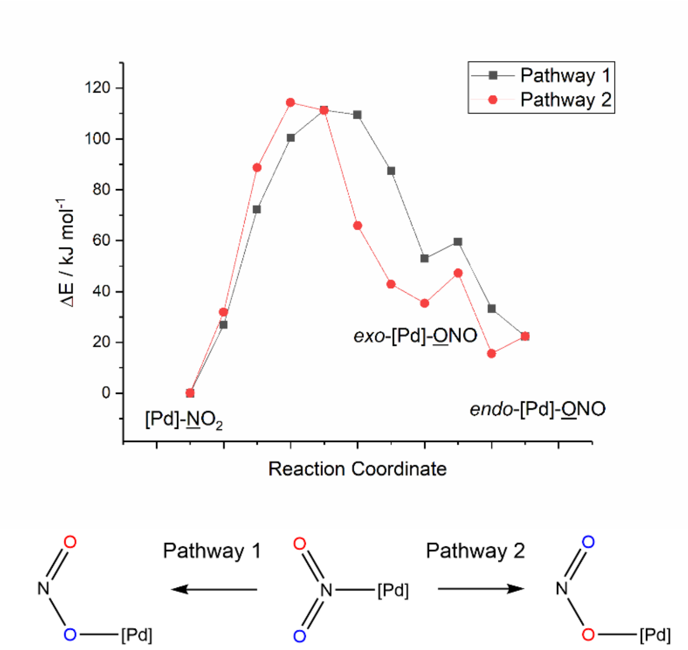

Figure S32: Summary of the energy pathways between the nitro-(η^1-NO_2) and *endo*-nitrito-(η^1-ONO) isomers of 1 as calculated by the NEB method

The structures of both the 100% nitro-(η^1-NO_2) and 100% nitrito-(η^1-ONO) isomers of complex **1** were used as inputs for a NEB calculation. These structures were prepared for the calculation by ensuring that each individual atom in the listed structure appears in the same order in the starting geometry and output geometry. This highlighted two possible pathways for the isomerism, designated pathway 1 and pathway 2. In pathway 1, it is assumed that each oxygen atom in the nitro group terminates on the same side of the nitrogen atom that they originate from. In pathway 2, it is suggested that the nitro ligand undergoes a 180° rotation perpendicular to the axis of the Pd-N bond and therefore that the oxygen atoms terminate on the opposite side from their origin. The full energy pathways are shown in Figure S32.

A video compilation of each pathway is also provided in the supplementary file **NEBpathways.avi**.

The activation energies of the two processes are very close and reflects the similarities in the transition state geometry (Figure S33). It is therefore likely that both pathways are viable isomerism mechanisms and contribute to overall conversion, at least energetically. Pathway 1 would be the most favourable pathway when taking topotactic considerations into account, as this route would involve the least atomic rearrangements in the solid-state.

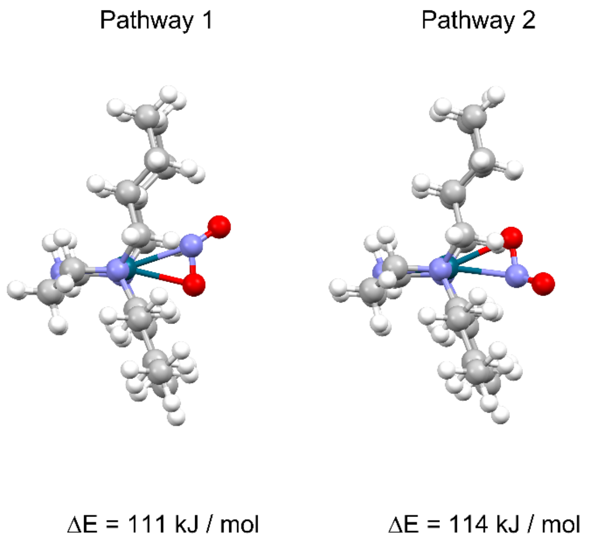

Figure S33: The converged transition state resulting from the NEB calculations on the isomerism of 1. Energy relative to nitro-(η^1-NO_2) isomer of 1.

A commonality between the two pathways is that they both exhibit a local energy minimum between the highest-energy transition state and the final nitrito-(η^1-ONO) isomer. The geometries of these local energy minima are shown in Figure S34 and correspond to the proposed *exo* nitrito-(η^1-ONO) isomers of **1.**

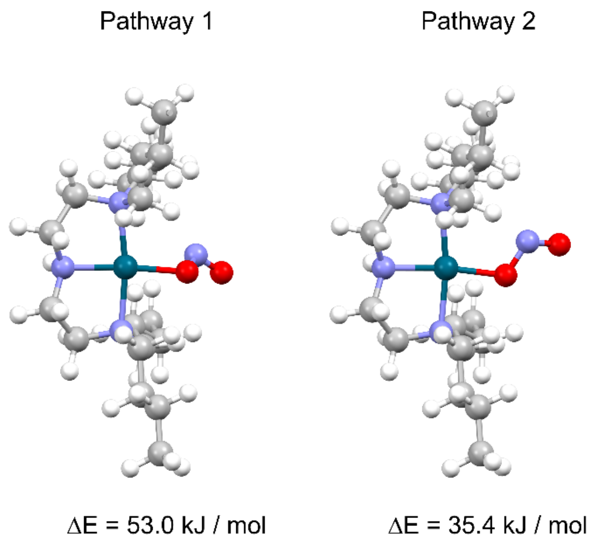

Figure S34: The geometries of the local energy minima state resulting from the NEB calculations on the isomerism of 1 corresponding to the proposed *exo* nitrito-(η^1-ONO) isomers. Energy relative to nitro-(η^1-NO_2) isomer of 1.

Figure S32 indicates that these local minima are very shallow relative to the overall isomerism energy. Although the *exo* isomers are present regardless of the isomerism pathway, the *exo* isomers formed are likely to continue to the lower-energy *endo* isomer almost immediately, and thus are not expected to exhibit a very long lifetime.

Supplementary Methods

(i) Pump-multiprobe synchronisation

The GDA interfaces with the diffractometer control software, EPICS,[8] to set the start and end points for the φ scans, the scan velocity and to set up the detector for collecting images. The "position compare" TTL output on the diffractometer, which goes high while the φ-axis is swept over the desired angle range, is used as the master signal to trigger the detector and, *via* the GDA and TFG2 timeframe generator, the LED driver. The jitter in the response of the TFG2 and Pilatus 300 K are 1.5625 and 10 ns, respectively, and completely negligible on the timescale of our experiments. Experiment metadata, including the diffractometer positions, detector responses and LED switching, are logged with timestamps to monitor the synchronisation. A schematic representation of the pump-multiprobe synchronisation is available in the Supplementary Information (Figure S32).

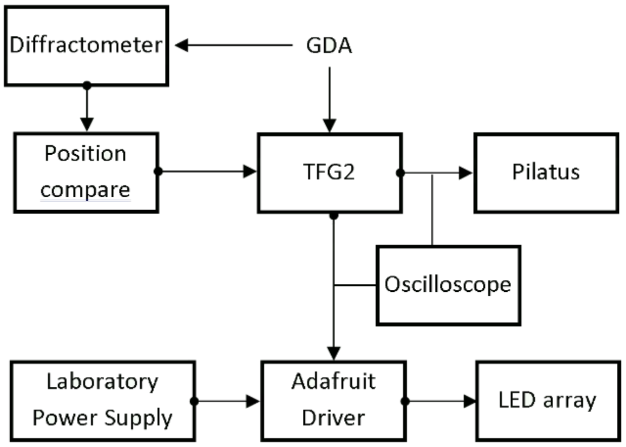

Figure S35 Schematic diagram outlining the communication connections between the diffractometer, LED pump array, Pilatus detector, TFG2 function generator and GDA software to enable the timing synchronisation in the pump-multiprobe experiments.

(ii) Pump-multiprobe data-collection strategy design. In order to maximise coverage of reciprocal space whilst minimising the downtime due to diffractometer movement between data-collection positions, we devised a collection strategy based on scanning the phi (φ)-axis. A target pump-probe cycle time t_{cyc} is chosen and numerical simulations are used to optimise the excitation pulse length t_{exc}, decay period t_{dec} and measurement temperature T to maximise the difference between the ES populations $\alpha(t)$ at the start of the cycle ($t = 0$) and after the pump pulse ($t = t_{\mathrm{exc}}$). We then select an acquisition time t_{acq}, and an appropriate scan width Δφ and rotation speed are determined. There is a small time delay between acquisition periods while the diffractometer motors are reversed, and the synchronisation between the pump, diffractometer and detector requires an integral number of acquisitions in each of the excitation and decay periods. The chosen t_{cyc}, t_{exc}, t_{dec} and t_{acq}, together with information from the simulations, thus also determines the number of probe measurements per cycle.

To obtain a good quality, complete set of single-crystal X-ray data at each t, a sufficient number of φ scans must be collected to cover the reciprocal space of the crystal. This is achieved by performing two 180 ° φ scans at ω = – 90 °, κ = 0 °, 2θ = 12 ° (scan 1) and ω = – 90 °, κ = 60 °, 2θ = 12 ° (scan

2). Δφ must be chosen carefully to balance fast data collection and a short overall experiment duration with recording accurate diffraction intensities by sampling reflections multiple times across their profile. The φ-axis rotation speed was 2 ° / s. To minimise the time overhead of moving the diffractometer axes, we perform both forwards and reverse φ scans and require an odd number of probe measurements so that the diffractometer ends each pump-probe cycle in the correct start position for the following scan (c.f. Figure 1). The Δφ and timings used in the experiments in this work are listed in Table 1.

As an example, with a target t_{cyc} = 170 s, the simulations predict t_{exc} = 52.7 s, t_{dec} = 117.3 s and a measurement temperature of 260 K. We select a t_{acq} of 8 s, for which an appropriate $\Delta\varphi$ is 16 ° with a φ-axis rotation speed of 2 ° / s. Including overheads, we obtain 9.6 s per time delay, allowing for a total of 17 Δt within the 170 s t_{cyc}. The t_{exc} and t_{dec} are adjusted to 55 and 115 s to include an integral number of probe measurements, giving 5 and 12 Δt during the excitation and decay periods, respectively. The diffractometer φ-axis will therefore scan the same angle range Δφ 17 times during each cycle. Each φ scan covers 180 °, so a total of 11 complete pump-multiprobe cycles are recorded for a full scan, and 22 cycles across the complete experiment (i.e. 11 complete cycles for each of the two φ scans with the ω, κ and 2θ positions given above).

(iii) Data Processing

Once the data collection has begun, the automated processing procedures can be started in parallel.[9]
The diffraction images are sorted on-the-fly during the data collection into separate directories for each delay time t recorded. This ensures that the diffraction images are in the correct format for routine SCXRD processing. Automated data indexing and integration are performed by DIALS,[10] while data scaling and absorption correction are applied by AIMLESS,[11] all of which are run through the xia2 interface. Initial structure refinement is also performed automatically by running the processed data through SHELXL and importing a model solution containing both the GS nitro-(η^1-NO_2) and ES nitrito-(η^1-ONO) isomers as a standard disorder model that utilises SHELX PART instructions to refine the GS:ES isomer occupancy ratio (and therefore the conversion fraction) as a free variable. Once the refinement has converged, the conversion fraction is extracted from the resulting SHELX CIF file, and the conversion fractions for all t are tabulated in a separate text file. This enables a rapid initial assessment of the pump-multiprobe experiment results and hence fast decision-making with regards to adjusting variables such as the temperature. For each dataset, complete automated data processing takes *c.a.* 15 minutes to complete and requires no user intervention. We take advantage of the Diamond Light Source computing cluster, enabling us to process all of the datasets from a pump-multiprobe experiment simultaneously on different cluster nodes, so that the number of t recorded does not affect the overall data-processing time.
To finalise the crystal structures and confirm the presence and occupancy of the photo-induced ES isomer, the data were later analysed manually using Olex2.[12] This manual processing was performed to ensure data were suitable for publication, but in practice we found that the automatic data processing generated structures that closely resembled the final versions prepared manually. Full details of the structure refinement are given in the main manuscript.

(iv) Photodifference map generation

Fourier electron-density difference maps between the GS and photo-excited models were generated by refining the GS structure (atomic coordinates and anisotropic displacement parameters) against the photo-excited reflection file (HKL file) with SHELXL. The GS parameters were prevented from changing significantly by using a DAMP 20000 instruction, and a LIST 3 instruction was chosen to output the correct FCF file required to generate the photo-difference maps. Photo-difference maps

were plotted using the Maps tool in Olex2, and individual images for each t were generated and combined into molecular movies using the ImageMagick software.[13]

(v) Timepix experiments

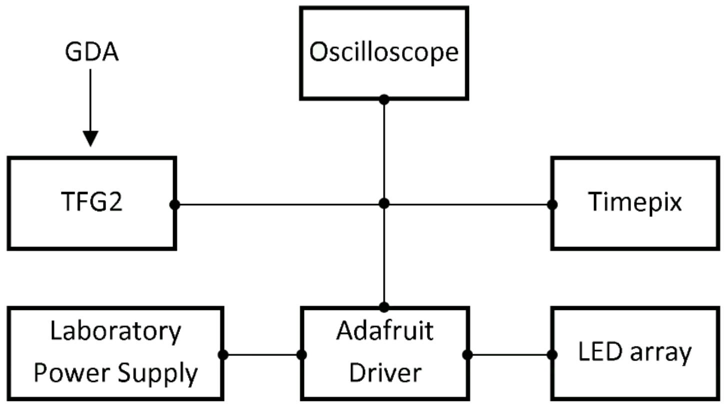

Figure S36 Schematic diagram outlining the communication connections between the diffractometer, LED pump array, Timepix3 detector, TFG2 function generator and GDA software to enable the timing synchronisation in the Timepix experiments.

Supplementary References

1. Hatcher, L.E. Raising the (metastable) bar: 100% photo-switching in $[Pd(Bu4dien)(\eta^1\text{-}\underline{N}O_2)]^+$ approaches ambient temperature. *CrystEngComm* **18**, 4180-4187 (2016).

2. Diamond General Data Aquisition (GDA) software, <http://www.opengda.org/> Accessed 04/01/2022.

3. Hatcher, L.E. *et al.* Monitoring photo-induced population dynamics in metastable linkage isomer crystals: a crystallographic kinetic study of $[Pd(Bu_4dien)NO_2]BPh_4$. *Phys. Chem. Chem. Phys.* **20**, 5874-5886 (2018).

4. Avrami, M. Kinetics of Phase Change. III. Granulation, Phase Change, and Microstructure. *J. Chem. Phys.* **9**, 177-184 (1941).

5. Avrami, M. Kinetics of Phase Change. II. Transformation-Time Relations for Random Distribution of Nuclei. *J. Chem. Phys.* **8**, 212-224 (1940).

6. Avrami, M. Kinetics of Phase Change. I. General Theory. *J. Chem. Phys.* **7**, 1103-1112 (1939).

7. Schmokel, M.S., Kaminski, R., Benedict, J.B. & Coppens, P. Data scaling and temperature calibration in time-resolved photocrystallographic experiments. *Acta Cryst. A* 66, 632-636 (2010).

8. Experimental Physics and Industrial Control System (EPICS), <https://epics.anl.gov/about.php> Accessed 04/01/2022.

9. Gerstel M., Ashton A., Gildea R.J., Levik K. & G. Winter. Data Analysis Infrastructure for Diamond Light Source Macromolecular & Chemical Crystallography and Beyond. *International Conference on Accelerator and Large Experimental Physics Control Systems (17th)*, New York, NY, USA (2019).

10. Winter, G. *et al.* DIALS: implementation and evaluation of a new integration package. *Acta Cryst. D.* **74**, 85-97 (2018).

11. Evans, P.R. & Murshudov, G.N. How good are my data and what is the resolution? *Acta Cryst. D.* **69**, 1204-1214 (2013).

12. Dolomanov, O.V., Bourhis, L.J., Gildea, R.J., Howard, J.A.K. & Puschmann, H. OLEX2: a complete structure solution, refinement and analysis program. *J. Appl. Cryst.* **42,** 339-341 (2009).

13. The ImageMagick Development Team. ImageMagick (2021). <https://imagemagick.org> Accessed 04/01/2021.
