# Supplementary material for: LED-pump-X-ray-multiprobe crystallography for sub-second timescales
Source: Commun Chem. 2022 Aug 26;5:102. doi: 10.1038/s42004-022-00716-1 (PMC9814726; doi:10.1038/s42004-022-00716-1)
Supplement: Supplementary file 3 — Description of Additional Supplementary Files [file 42004_2022_716_MOESM3_ESM.docx]

Description of Additional Supplementary Files

**File name:** Supplementary Data 1

**Description:** Cif files for all 178 structures

**File name:** Supplementary Data 2

**Description:** Check cif files for all 178 structures determined

**File name:** Supplementary Data 3

**Description:** Electronic structure calculations for N-bound molecule

**File name:** Supplementary Data 4

**Description:** Electronic structure calculation for endo O-bound molecule

**File name:** Supplementary Data 5

**Description:** Wilson plots for 260 K data

**File name:** Supplementary Data 6

**Description:** Wilson plots for 284 K data

**File name:** Supplementary Movie 1

**Description:** Movie of transition between 0-4s

**File name:** Supplementary Movie 2

**Description**: Movie of transition between 0-8s

**File name:** Supplementary Movie 3

**Description:** Movie of transition between 1-6 s

**File name:** Supplementary Movie 4

**Description:** Movie of transition state at 4s

**File name:** Supplementary Movie 5

**Description:** Movie of transition state at 8s

**File name:** Supplementary Movie 6

**Description:** Movie of modelled reaction pathway
