# Supplementary figures and images for: LED-pump-X-ray-multiprobe crystallography for sub-second timescales
Source: Commun Chem. 2022 Aug 26;5:102. doi: 10.1038/s42004-022-00716-1 (PMC9814726; doi:10.1038/s42004-022-00716-1)

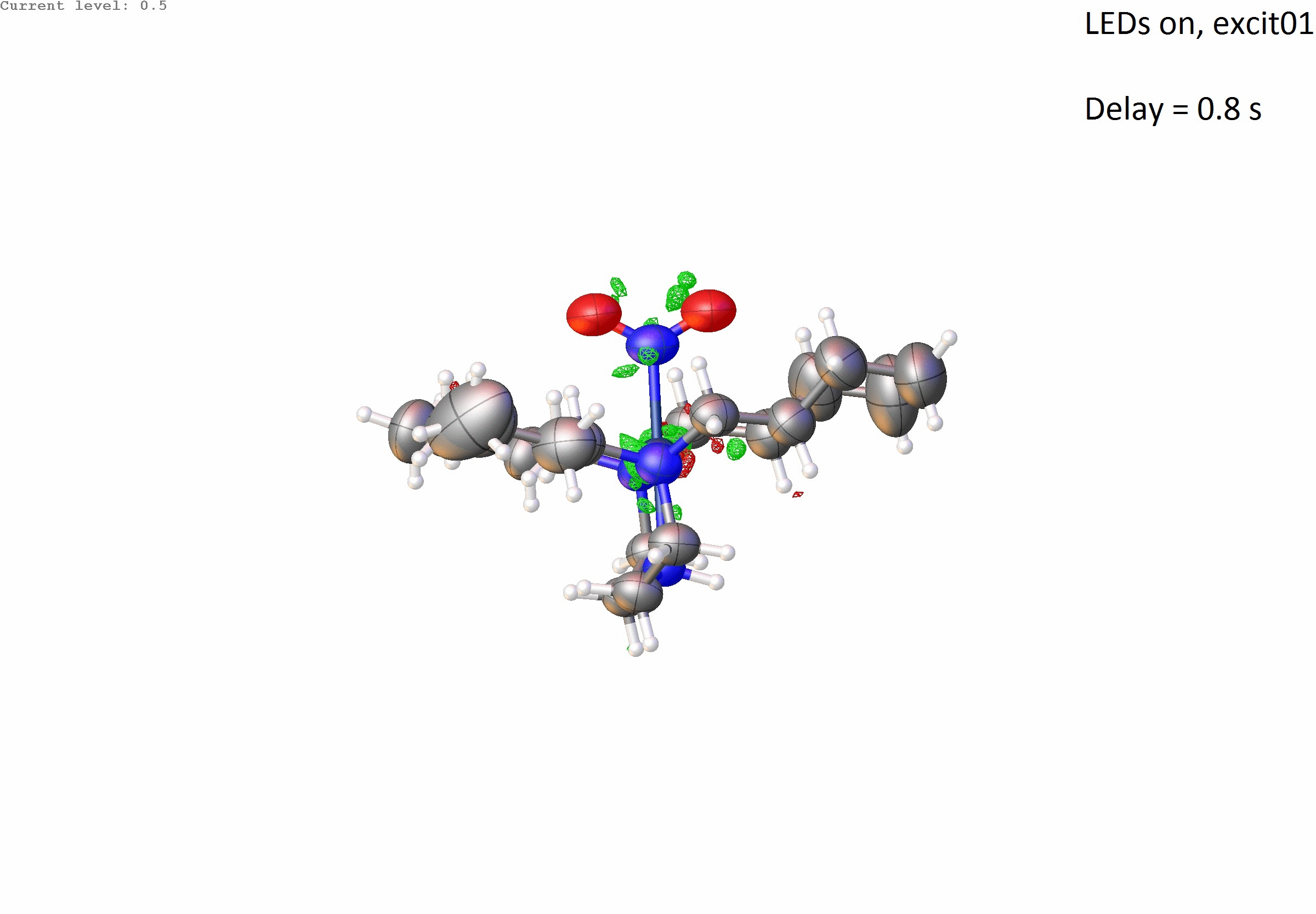

Supplement: Supplementary file 4 — Supplementary Movie 1 [file 42004_2022_716_MOESM4_ESM.gif]

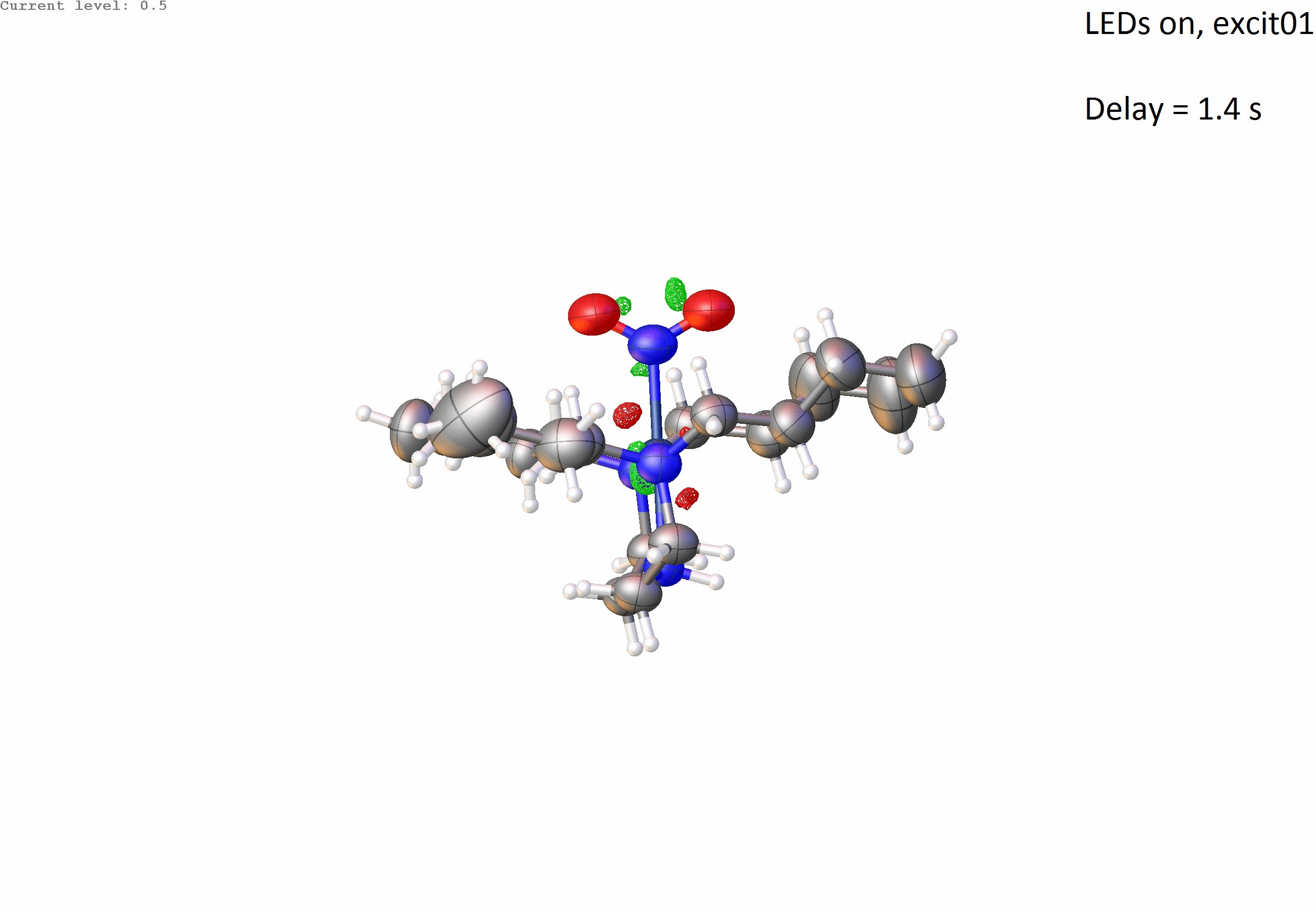

Supplement: Supplementary file 5 — Supplementary Movie 2 [file 42004_2022_716_MOESM5_ESM.gif]

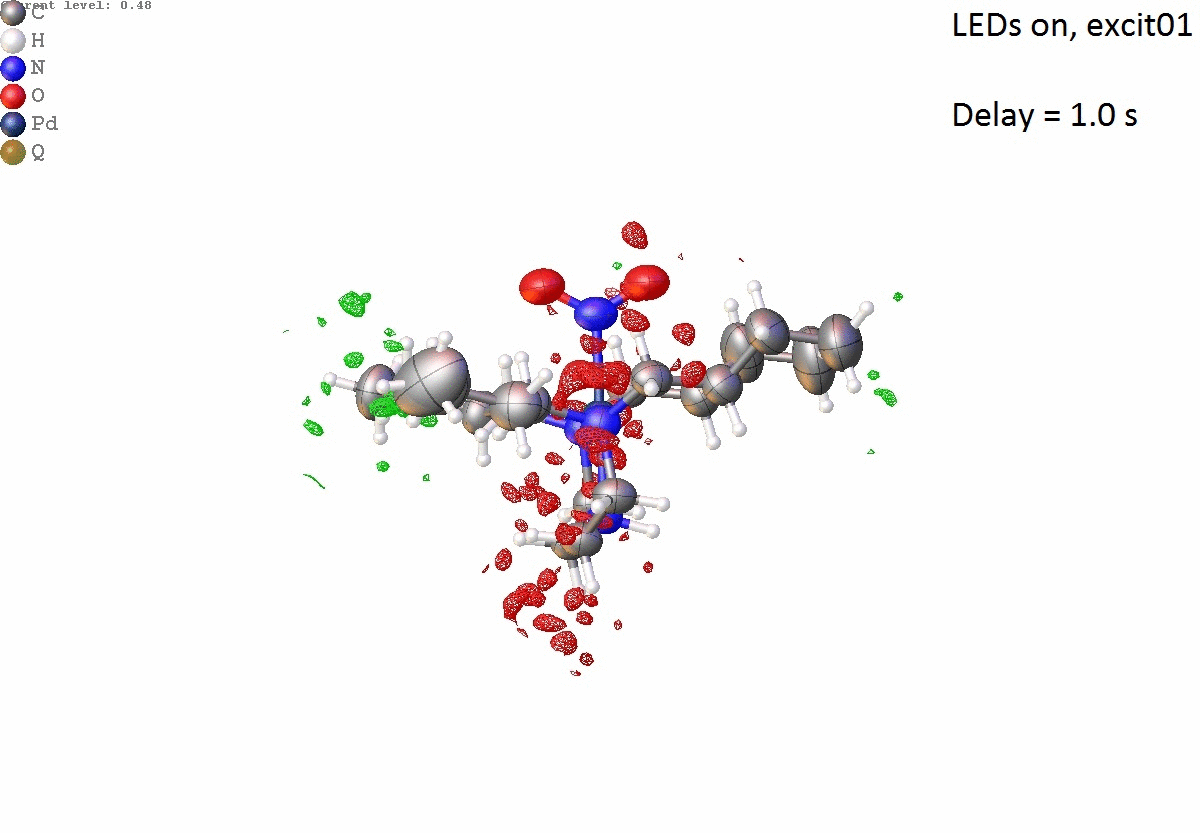

Supplement: Supplementary file 6 — Supplementary Movie 3 [file 42004_2022_716_MOESM6_ESM.gif]

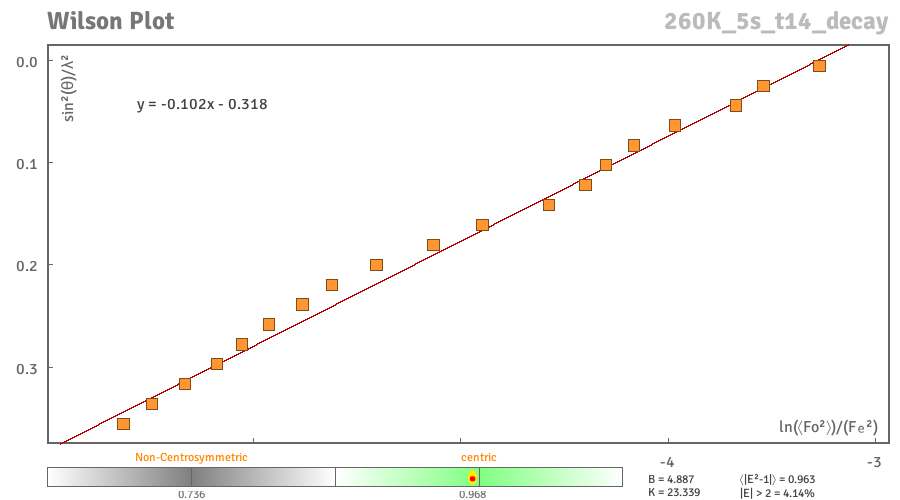

Supplement: Supplementary file 14 — Supplementary Data 5 [file 42004_2022_716_MOESM14_ESM.zip › 260K_8s/260K_5s_t14_decay_wilson.png]

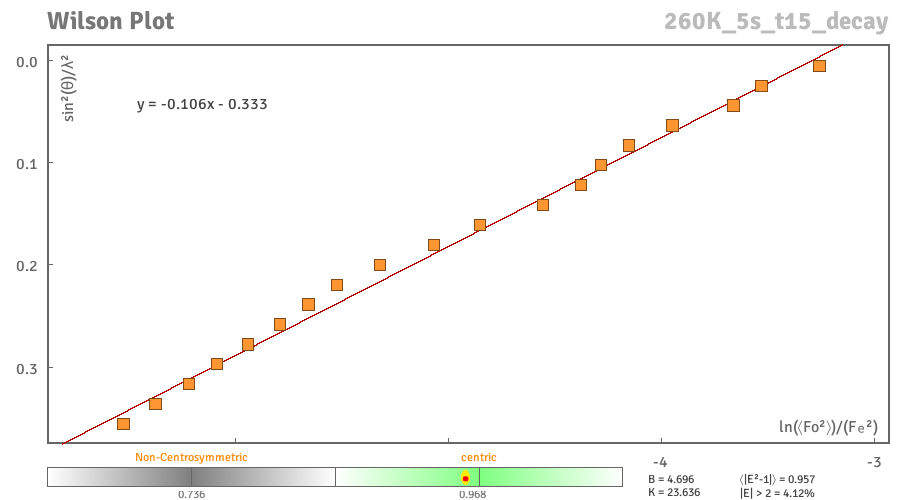

Supplement: Supplementary file 14 — Supplementary Data 5 [file 42004_2022_716_MOESM14_ESM.zip › 260K_8s/260K_5s_t15_decay_wilson.png]

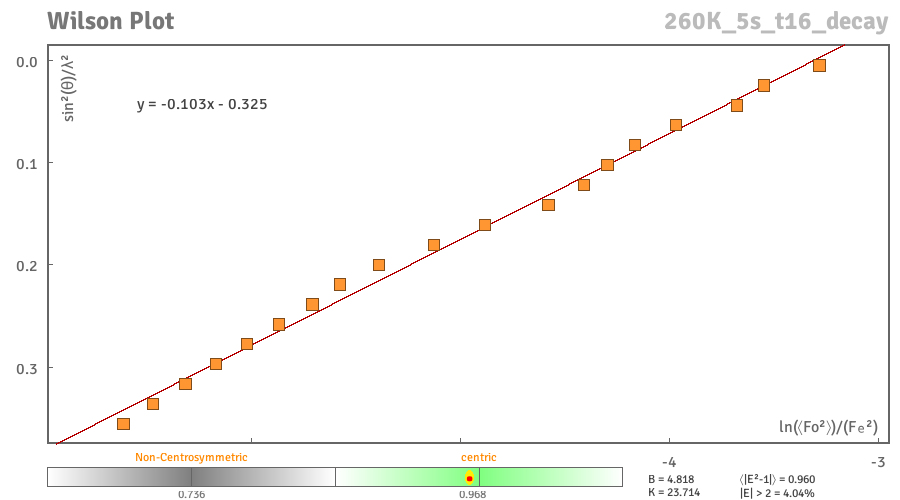

Supplement: Supplementary file 14 — Supplementary Data 5 [file 42004_2022_716_MOESM14_ESM.zip › 260K_8s/260K_5s_t16_decay_wilson.png]

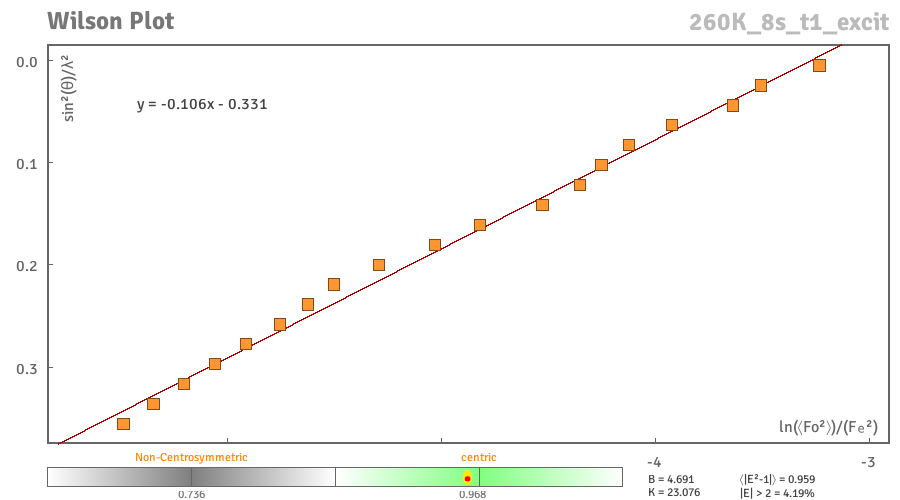

Supplement: Supplementary file 14 — Supplementary Data 5 [file 42004_2022_716_MOESM14_ESM.zip › 260K_8s/260K_8s_t1_excit_wilson.png]

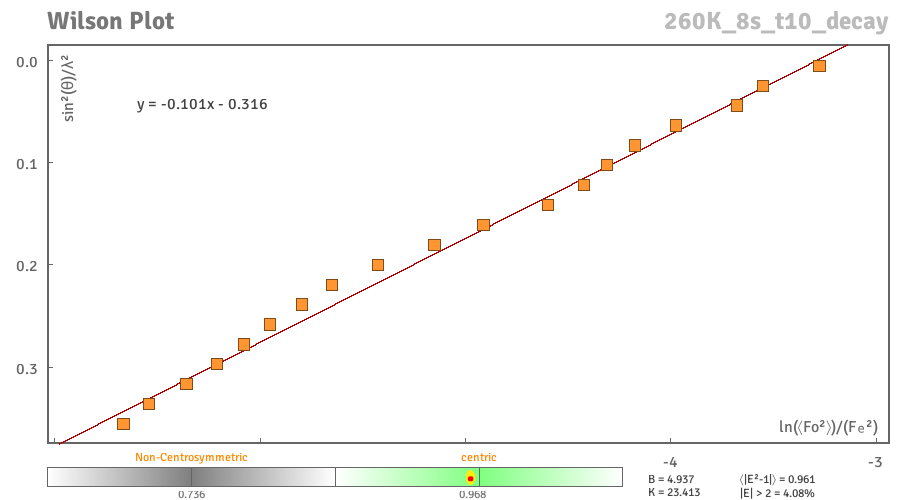

Supplement: Supplementary file 14 — Supplementary Data 5 [file 42004_2022_716_MOESM14_ESM.zip › 260K_8s/260K_8s_t10_decay_wilson.png]

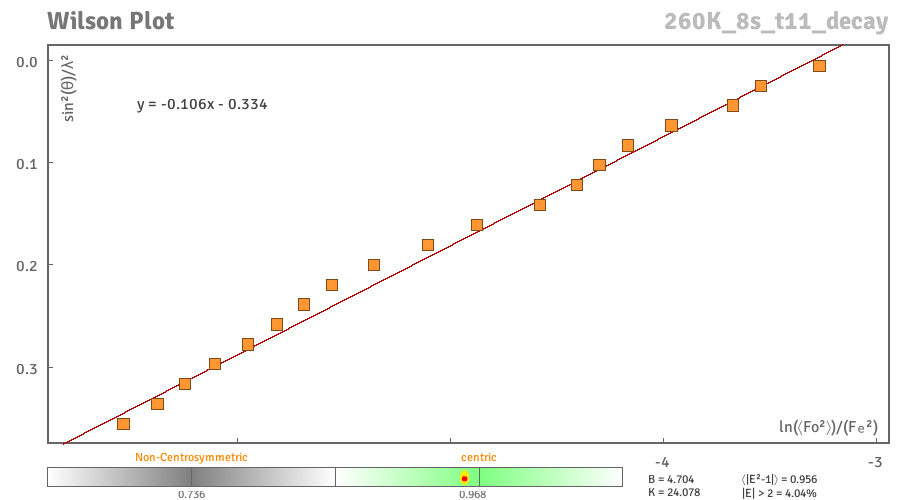

Supplement: Supplementary file 14 — Supplementary Data 5 [file 42004_2022_716_MOESM14_ESM.zip › 260K_8s/260K_8s_t11_decay_wilson.png]

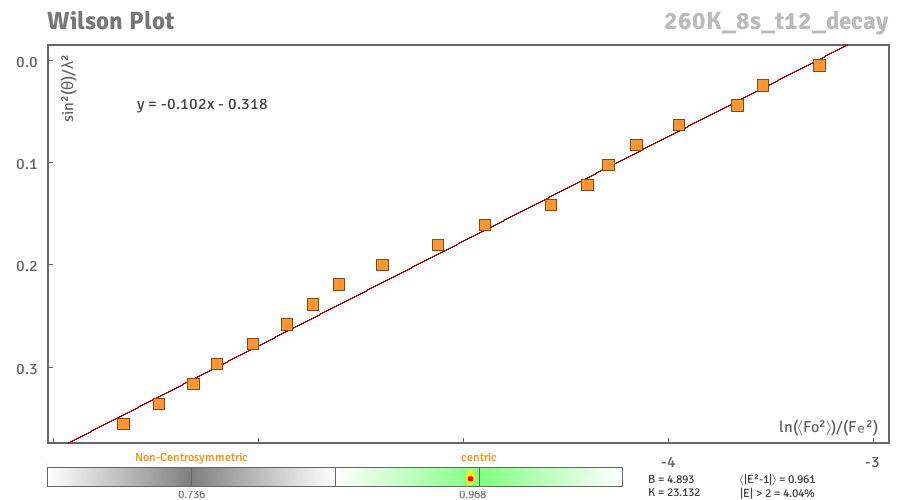

Supplement: Supplementary file 14 — Supplementary Data 5 [file 42004_2022_716_MOESM14_ESM.zip › 260K_8s/260K_8s_t12_decay_wilson.png]

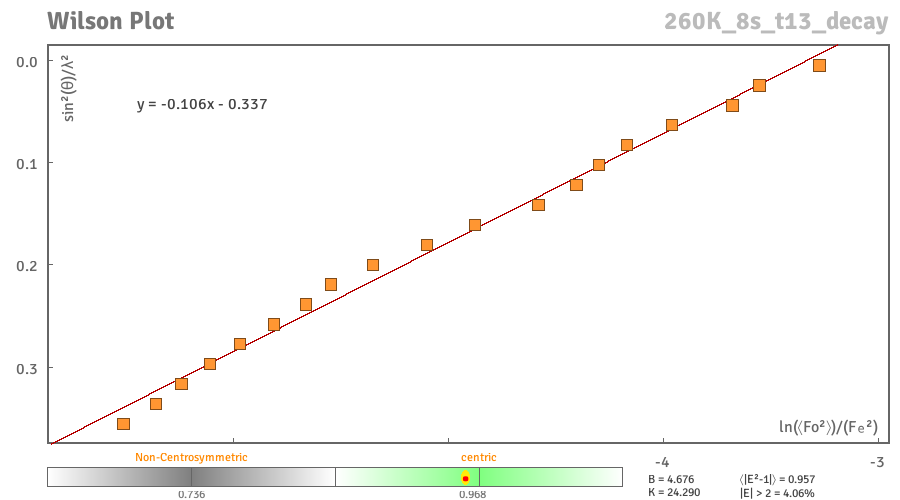

Supplement: Supplementary file 14 — Supplementary Data 5 [file 42004_2022_716_MOESM14_ESM.zip › 260K_8s/260K_8s_t13_decay_wilson.png]

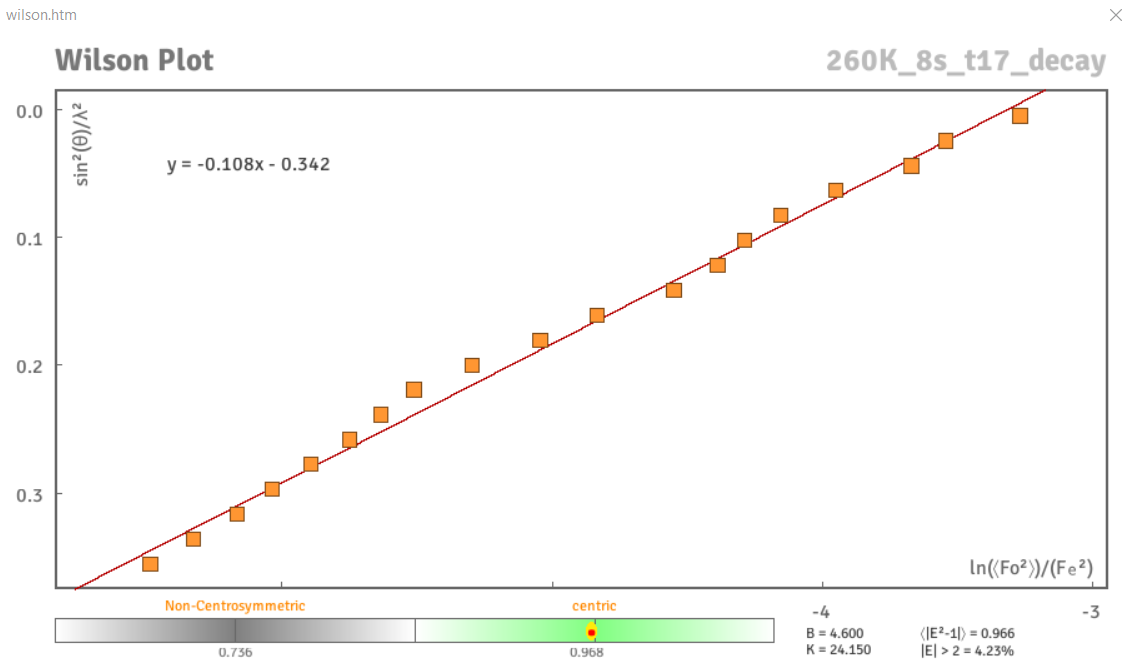

Supplement: Supplementary file 14 — Supplementary Data 5 [file 42004_2022_716_MOESM14_ESM.zip › 260K_8s/260K_8s_t17_decay_Wilson_Plot.png]

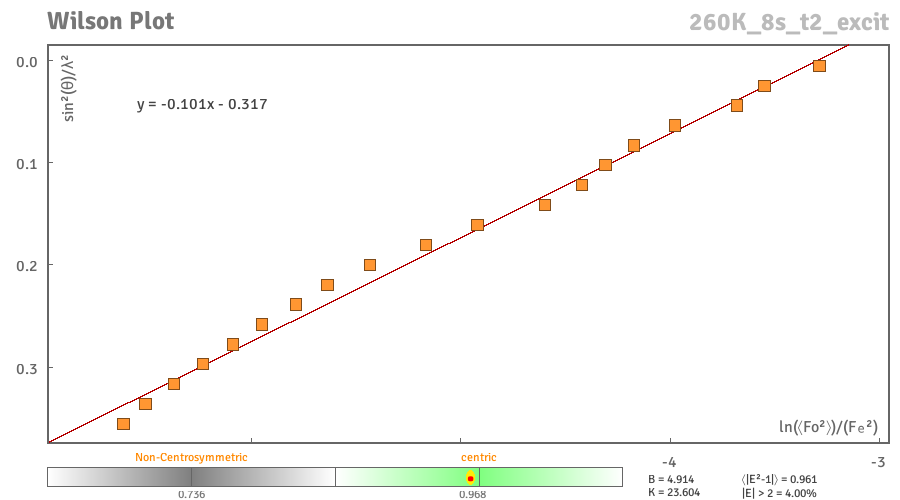

Supplement: Supplementary file 14 — Supplementary Data 5 [file 42004_2022_716_MOESM14_ESM.zip › 260K_8s/260K_8s_t2_excit_wilson.png]

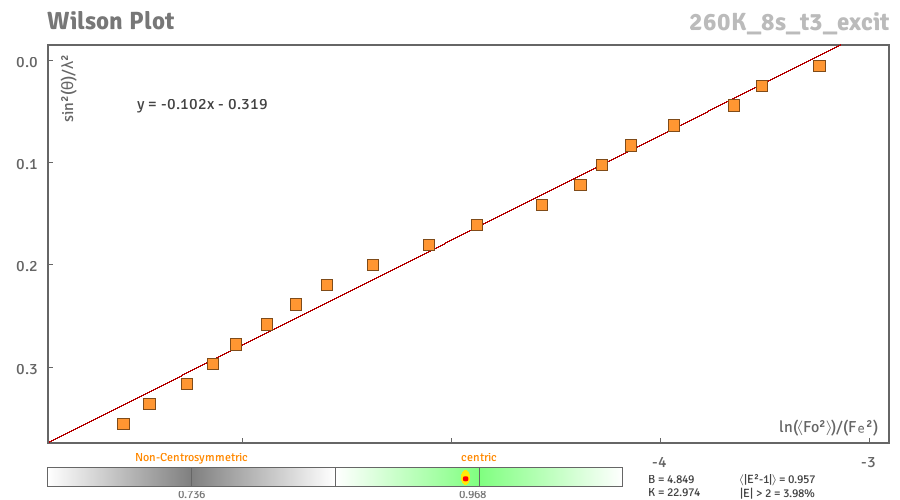

Supplement: Supplementary file 14 — Supplementary Data 5 [file 42004_2022_716_MOESM14_ESM.zip › 260K_8s/260K_8s_t3_excit_wilson.png]

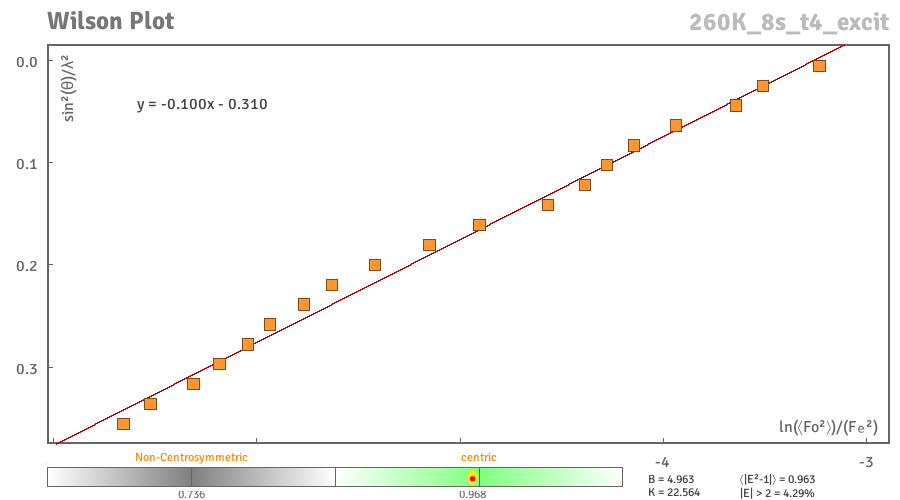

Supplement: Supplementary file 14 — Supplementary Data 5 [file 42004_2022_716_MOESM14_ESM.zip › 260K_8s/260K_8s_t4_excit_wilson.png]

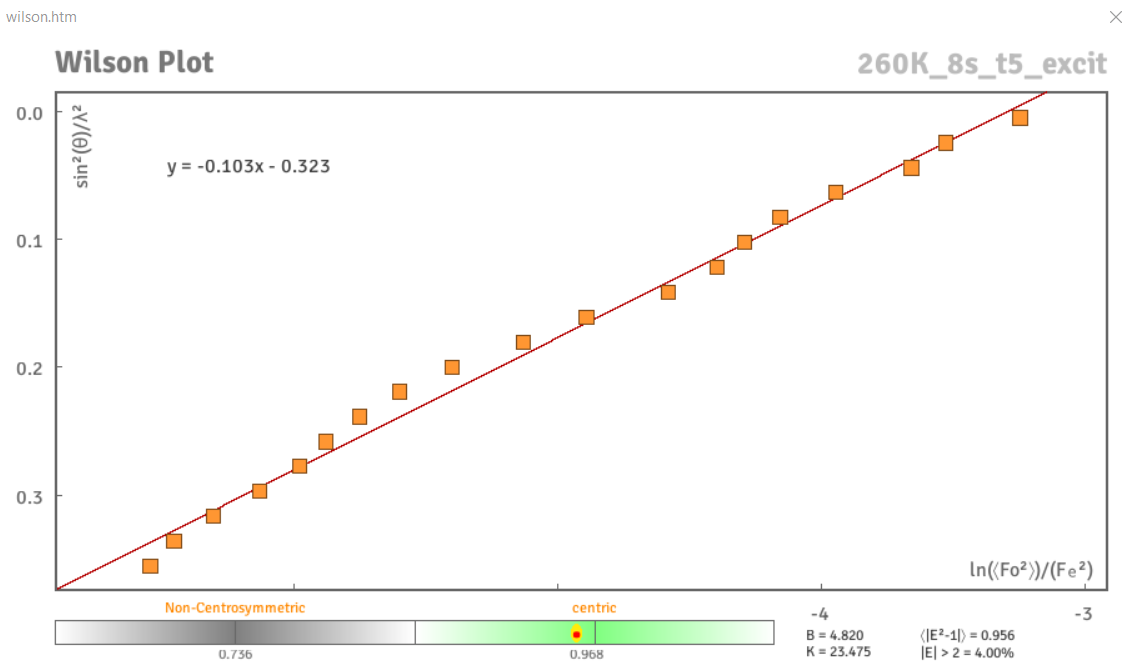

Supplement: Supplementary file 14 — Supplementary Data 5 [file 42004_2022_716_MOESM14_ESM.zip › 260K_8s/260K_8s_t5_excit_ Wilson_Plot.png]

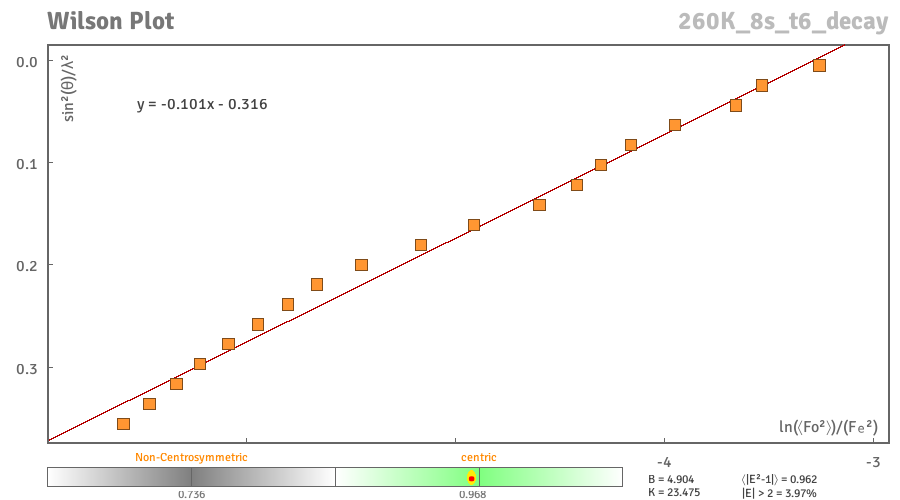

Supplement: Supplementary file 14 — Supplementary Data 5 [file 42004_2022_716_MOESM14_ESM.zip › 260K_8s/260K_8s_t6_decay_wilson.png]

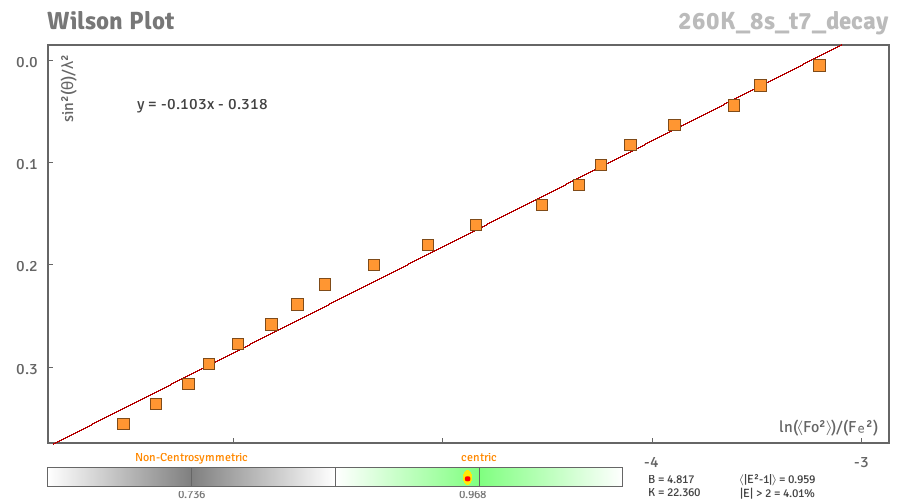

Supplement: Supplementary file 14 — Supplementary Data 5 [file 42004_2022_716_MOESM14_ESM.zip › 260K_8s/260K_8s_t7_decay_wilson.png]

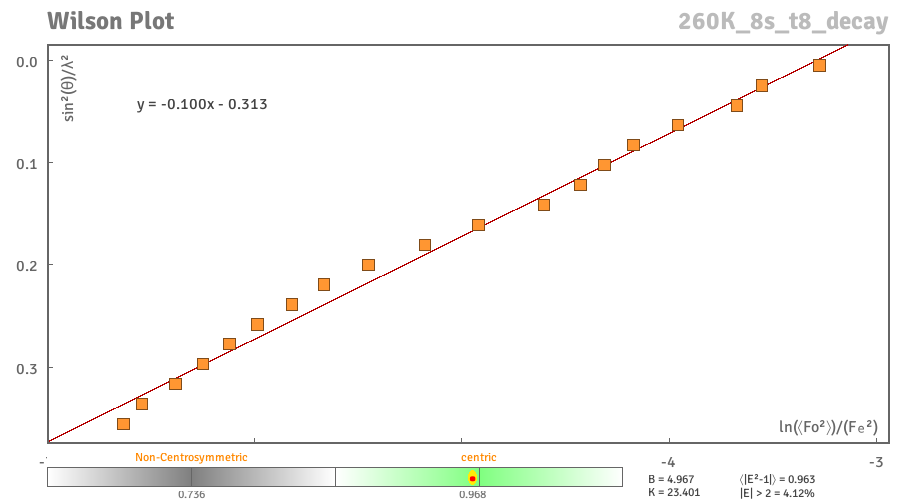

Supplement: Supplementary file 14 — Supplementary Data 5 [file 42004_2022_716_MOESM14_ESM.zip › 260K_8s/260K_8s_t8_decay_wilson.png]

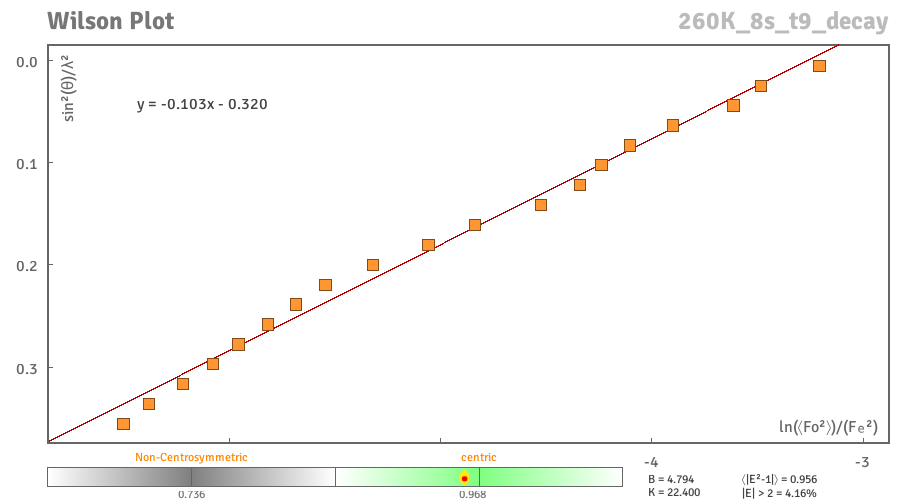

Supplement: Supplementary file 14 — Supplementary Data 5 [file 42004_2022_716_MOESM14_ESM.zip › 260K_8s/260K_8s_t9_decay_wilson.png]
