# Supplementary material for: LED-pump-X-ray-multiprobe crystallography for sub-second timescales
Source: Commun Chem. 2022 Aug 26;5:102. doi: 10.1038/s42004-022-00716-1 (PMC9814726; doi:10.1038/s42004-022-00716-1)

checkCIF/PLATON report

Structure factors have been supplied for datablock(s) 260K_8s_t17_decay

THIS REPORT IS FOR GUIDANCE ONLY. IF USED AS PART OF A REVIEW PROCEDURE FOR PUBLICATION, IT SHOULD NOT REPLACE THE EXPERTISE OF AN EXPERIENCED CRYSTALLOGRAPHIC REFEREE.

No syntax errors found. CIF dictionary Interpreting this report

Datablock: 260K_8s_t17_decay

Bond precision: C-C = 0.0077 A Wavelength=0.53400

Cell:	a=11.6715(4)	b=13.5688(4)	c=30.2352(10)
	alpha=90	beta=93.267(3)	gamma=90
Temperature:	260 K		

	Calculated	Reported
Volume	4780.5(3)	4780.5(3)
Space group	P 21/n	P 21/n
Hall group	-P 2yn	-P 2yn
Moiety formula	C20 H45 N4 O2 Pd, C24 H20 B, C4 H8 O	C20 H45 N4 O2 Pd, C24 H20 B, C4 H8 O
Sum formula	C48 H73 B N4 O3 Pd	C48 H73 B N4 O3 Pd
Mr	871.31	871.31
Dx,g cm-3	1.211	1.211
Z	4	4
Mu (mm-1)	0.206	0.202
F000	1856.0	1856.0
F000'	1846.09	
h,k,lmax	14,16,37	14,16,37
Nref	9787	9740
Tmin,Tmax	0.980,0.990	0.996,1.000
Tmin'	0.980	

Correction method= # Reported T Limits: Tmin=0.996 Tmax=1.000
AbsCorr = EMPIRICAL

Data completeness= 0.995 Theta(max)= 19.496

R(reflections)= 0.0576(4366) wR2(reflections)= 0.1479(9740)

S = 0.864 Npar= 526

The following ALERTS were generated. Each ALERT has the format
test-name_ALERT_alert-type_alert-level.
Click on the hyperlinks for more details of the test.

Alert level C

```
PLAT026_ALERT_3_C Ratio Observed / Unique Reflections (too) Low ..        45% Check
PLAT220_ALERT_2_C NonSolvent   Resd 1  C   Ueq(max)/Ueq(min) Range        3.3 Ratio
PLAT222_ALERT_3_C NonSolvent Resd 1  H   Uiso(max)/Uiso(min) Range        4.6 Ratio
PLAT241_ALERT_2_C High   'MainMol' Ueq as Compared to Neighbors of        C43 Check
PLAT242_ALERT_2_C Low    'MainMol' Ueq as Compared to Neighbors of        Pd1 Check
PLAT242_ALERT_2_C Low    'MainMol' Ueq as Compared to Neighbors of         C7 Check
PLAT242_ALERT_2_C Low    'MainMol' Ueq as Compared to Neighbors of        C18 Check
PLAT242_ALERT_2_C Low    'MainMol' Ueq as Compared to Neighbors of        C27 Check
PLAT242_ALERT_2_C Low    'MainMol' Ueq as Compared to Neighbors of        C39 Check
PLAT260_ALERT_2_C Large Average Ueq of Residue Including       Pd1      0.101 Check
PLAT260_ALERT_2_C Large Average Ueq of Residue Including        O3      0.122 Check
PLAT260_ALERT_2_C Large Average Ueq of Residue Including       O3A      0.122 Check
PLAT360_ALERT_2_C Short  C(sp3)-C(sp3) Bond  C19      - C20      .       1.40 Ang.
PLAT906_ALERT_3_C Large K Value in the Analysis of Variance ......     21.007 Check
PLAT906_ALERT_3_C Large K Value in the Analysis of Variance ......      3.473 Check
PLAT911_ALERT_3_C Missing FCF Refl Between Thmin & STh/L=    0.600         36 Report
```

Alert level G

```
ABSMU01_ALERT_1_G  Calculation of _exptl_absorpt_correction_mu
                not performed for this radiation type.
PLAT002_ALERT_2_G Number of Distance or Angle Restraints on AtSite         16 Note
PLAT007_ALERT_5_G Number of Unrefined Donor-H Atoms .............           1 Report
PLAT092_ALERT_4_G Check: Wavelength Given is not Cu,Ga,Mo,Ag,In Ka    0.53400 Ang.
PLAT171_ALERT_4_G The CIF-Embedded .res File Contains EADP Records          5 Report
PLAT172_ALERT_4_G The CIF-Embedded .res File Contains DFIX Records          2 Report
PLAT173_ALERT_4_G The CIF-Embedded .res File Contains DANG Records          1 Report
PLAT174_ALERT_4_G The CIF-Embedded .res File Contains FLAT Records          1 Report
PLAT175_ALERT_4_G The CIF-Embedded .res File Contains SAME Records          1 Report
PLAT187_ALERT_4_G The CIF-Embedded .res File Contains RIGU Records          1 Report
PLAT232_ALERT_2_G Hirshfeld Test Diff (M-X)  Pd1      --N1       .       10.0 s.u.
PLAT232_ALERT_2_G Hirshfeld Test Diff (M-X)  Pd1      --O1A      .        7.5 s.u.
PLAT301_ALERT_3_G Main Residue  Disorder .............(Resd  1  )         11% Note
PLAT302_ALERT_4_G Anion/Solvent/Minor-Residue Disorder (Resd  3  )       100% Note
PLAT302_ALERT_4_G Anion/Solvent/Minor-Residue Disorder (Resd  4  )       100% Note
PLAT304_ALERT_4_G Non-Integer Number of Atoms in ..... (Resd  3  )      10.06 Check
PLAT304_ALERT_4_G Non-Integer Number of Atoms in ..... (Resd  4  )       2.94 Check
PLAT398_ALERT_2_G Deviating  C-O-C    Angle From 120 for O3             105.4 Degree
PLAT398_ALERT_2_G Deviating  C-O-C    Angle From 120 for O3A            108.9 Degree
PLAT721_ALERT_1_G Bond    Calc     0.96000, Rep     0.97000 Dev...       0.01 Ang.
              C45A     -H45D            1_555   1_555 ........     #  138 Check
PLAT802_ALERT_4_G CIF Input Record(s) with more than 80 Characters          1 Info
PLAT860_ALERT_3_G Number of Least-Squares Restraints ............         490 Note
PLAT883_ALERT_1_G No Info/Value for _atom_sites_solution_primary .     Please Do !
PLAT910_ALERT_3_G Missing # of FCF Reflection(s) Below Theta(Min).          1 Note
PLAT912_ALERT_4_G Missing # of FCF Reflections Above STh/L=  0.600         11 Note
PLAT933_ALERT_2_G Number of OMIT Records in Embedded .res File ...          4 Note
PLAT941_ALERT_3_G Average HKL Measurement Multiplicity ...........        3.5 Low
PLAT978_ALERT_2_G Number C-C Bonds with Positive Residual Density.          0 Info
PLAT984_ALERT_1_G The Pd-f'=   -2.5040 Deviates from the B&C-Value    -2.4885 Check
```

```
PLAT985_ALERT_1_G The Pd-f"=    0.6150 Deviates from the B&C-Value     0.6018 Check
```

```
   0 ALERT level A = Most likely a serious problem - resolve or explain
   0 ALERT level B = A potentially serious problem, consider carefully
  16 ALERT level C = Check. Ensure it is not caused by an omission or oversight
  30 ALERT level G = General information/check it is not something unexpected

   5 ALERT type 1 CIF construction/syntax error, inconsistent or missing data
  18 ALERT type 2 Indicator that the structure model may be wrong or deficient
   9 ALERT type 3 Indicator that the structure quality may be low
  13 ALERT type 4 Improvement, methodology, query or suggestion
   1 ALERT type 5 Informative message, check
```

---

checkCIF publication errors

Alert level A

```
PUBL006_ALERT_1_A  _publ_requested_journal is missing
            e.g. 'Acta Crystallographica Section C'
PUBL008_ALERT_1_A  _publ_section_title is missing. Title of paper.
PUBL009_ALERT_1_A  _publ_author_name is missing. List of author(s) name(s).
PUBL010_ALERT_1_A  _publ_author_address is missing. Author(s) address(es).
PUBL012_ALERT_1_A  _publ_section_abstract is missing.
            Abstract of paper in English.
```

```
   5 ALERT level A = Data missing that is essential or data in wrong format
   0 ALERT level G = General alerts. Data that may be required is missing
```

Publication of your CIF

You should attempt to resolve as many as possible of the alerts in all categories. Often the minor alerts point to easily fixed oversights, errors and omissions in your CIF or refinement strategy, so attention to these fine details can be worthwhile. In order to resolve some of the more serious problems it may be necessary to carry out additional measurements or structure refinements. However, the nature of your study may justify the reported deviations from journal submission requirements and the more serious of these should be commented upon in the discussion or experimental section of a paper or in the "special_details" fields of the CIF. *checkCIF* was carefully designed to identify outliers and unusual parameters, but every test has its limitations and alerts that are not important in a particular case may appear. Conversely, the absence of alerts does not guarantee there are no aspects of the results needing attention. It is up to the individual to critically assess their own results and, if necessary, seek expert advice.

If level A alerts remain, which you believe to be justified deviations, and you intend to submit this CIF for publication in a journal, you should additionally insert an explanation in your CIF using the Validation Reply Form (VRF) below. This will allow your explanation to be considered as part of the review process.

```
# start Validation Reply Form
_vrf_PUBL006_GLOBAL
;
PROBLEM: _publ_requested_journal is missing
RESPONSE: ...
;
_vrf_PUBL008_GLOBAL
;
PROBLEM: _publ_section_title is missing. Title of paper.
RESPONSE: ...
;
_vrf_PUBL009_GLOBAL
;
PROBLEM: _publ_author_name is missing. List of author(s) name(s).
RESPONSE: ...
;
_vrf_PUBL010_GLOBAL
;
PROBLEM: _publ_author_address is missing. Author(s) address(es).
RESPONSE: ...
;
_vrf_PUBL012_GLOBAL
;
PROBLEM: _publ_section_abstract is missing.
RESPONSE: ...
;
# end Validation Reply Form
```

If you wish to submit your CIF for publication in Acta Crystallographica Section C or E, you should upload your CIF via the web. If you wish to submit your CIF for publication in IUCrData you should upload your CIF via the web. If your CIF is to form part of a submission to another IUCr journal, you will be asked, either during electronic submission or by the Co-editor handling your paper, to upload your CIF via our web site.

PLATON version of 13/07/2021; check.def file version of 13/07/2021

Datablock 260K_8s_t17_decay - ellipsoid plot

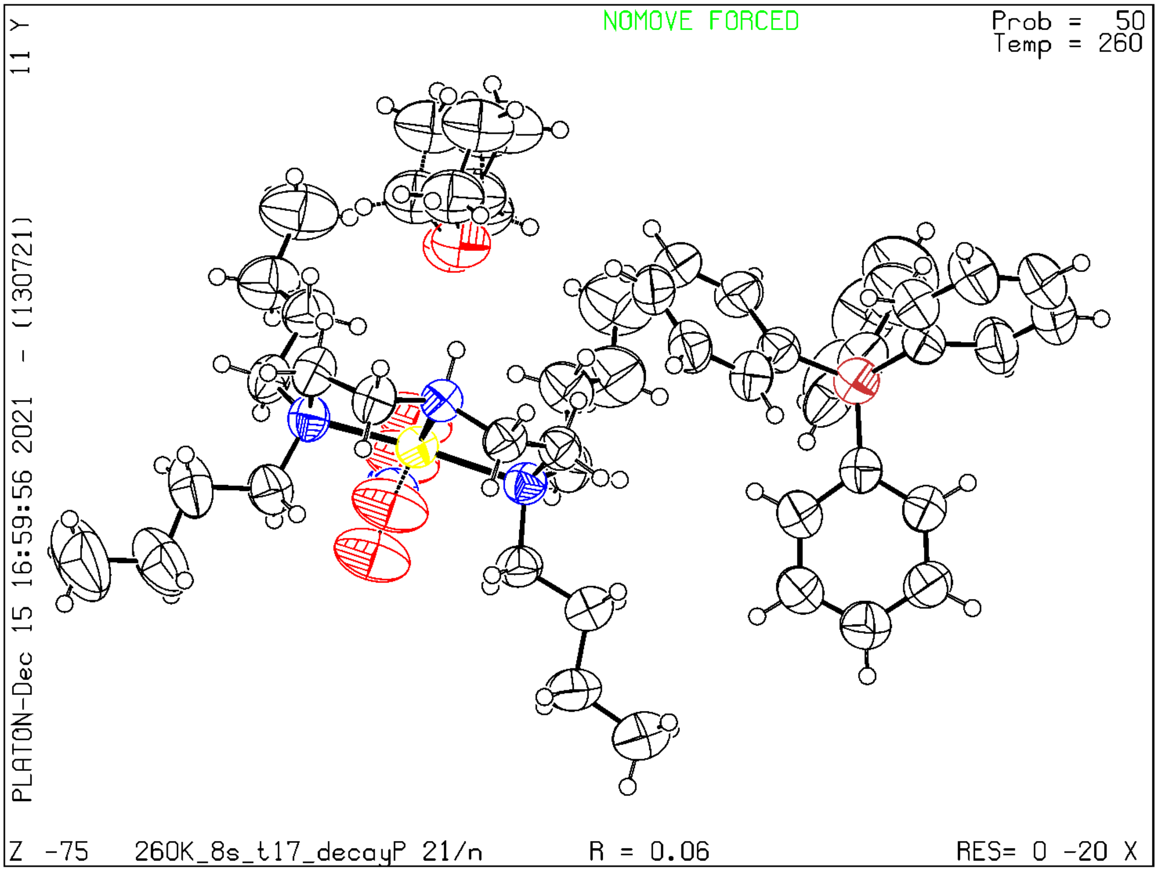

Supplement: Supplementary file 11 — Supplementary Data 2 [file 42004_2022_716_MOESM11_ESM.zip › Check-cifs/260K_8s_t17_decay_checkcif.pdf]
