# Supplementary material for: LED-pump-X-ray-multiprobe crystallography for sub-second timescales
Source: Commun Chem. 2022 Aug 26;5:102. doi: 10.1038/s42004-022-00716-1 (PMC9814726; doi:10.1038/s42004-022-00716-1)

checkCIF/PLATON report

Structure factors have been supplied for datablock(s) 265K_4s_t9_decay

THIS REPORT IS FOR GUIDANCE ONLY. IF USED AS PART OF A REVIEW PROCEDURE FOR PUBLICATION, IT SHOULD NOT REPLACE THE EXPERTISE OF AN EXPERIENCED CRYSTALLOGRAPHIC REFEREE.

No syntax errors found. CIF dictionary Interpreting this report

Datablock: 265K_4s_t9_decay

Bond precision:	C-C = 0.0064 A		Wavelength=0.53400
Cell:	a=11.6606(4)	b=13.5702(4)	c=30.1905(10)
	alpha=90	beta=93.406(3)	gamma=90
Temperature:	265 K		

	Calculated	Reported
Volume	4768.8(3)	4768.8(3)
Space group	P 21/n	P 21/n
Hall group	-P 2yn	-P 2yn
Moiety formula	C20 H45 N4 O2 Pd, C24 H20 B, C4 H8 O	C20 H45 N4 O2 Pd, C24 H20 B, C4 H8 O
Sum formula	C48 H73 B N4 O3 Pd	C48 H73 B N4 O3 Pd
Mr	871.31	871.31
Dx,g cm-3	1.214	1.214
Z	4	4
Mu (mm-1)	0.207	0.202
F000	1856.0	1856.0
F000'	1846.09	
h,k,lmax	14,16,37	14,16,37
Nref	9761	9722
Tmin,Tmax	0.980,0.990	0.997,1.000
Tmin'	0.980	

Correction method= # Reported T Limits: Tmin=0.997 Tmax=1.000
AbsCorr = EMPIRICAL

Data completeness= 0.996 Theta(max)= 19.496

R(reflections)= 0.0550(5241) wR2(reflections)= 0.1391(9722)

S = 0.900 Npar= 526

The following ALERTS were generated. Each ALERT has the format
test-name_ALERT_alert-type_alert-level.
Click on the hyperlinks for more details of the test.

Alert level C

```
PLAT220_ALERT_2_C NonSolvent   Resd 1  C   Ueq(max)/Ueq(min) Range         3.2 Ratio
PLAT222_ALERT_3_C NonSolvent Resd 1  H   Uiso(max)/Uiso(min) Range         4.4 Ratio
PLAT241_ALERT_2_C High   'MainMol' Ueq as Compared to Neighbors of         C43 Check
PLAT242_ALERT_2_C Low    'MainMol' Ueq as Compared to Neighbors of         Pd1 Check
PLAT242_ALERT_2_C Low    'MainMol' Ueq as Compared to Neighbors of         C18 Check
PLAT260_ALERT_2_C Large Average Ueq of Residue Including        O3       0.101 Check
PLAT260_ALERT_2_C Large Average Ueq of Residue Including       O3A       0.101 Check
PLAT360_ALERT_2_C Short  C(sp3)-C(sp3) Bond  C19      - C20      .        1.43 Ang.
PLAT906_ALERT_3_C Large K Value in the Analysis of Variance ......      12.102 Check
PLAT906_ALERT_3_C Large K Value in the Analysis of Variance ......       2.347 Check
PLAT911_ALERT_3_C Missing FCF Refl Between Thmin & STh/L=    0.600          37 Report
```

Alert level G

```
ABSMU01_ALERT_1_G  Calculation of _exptl_absorpt_correction_mu
                not performed for this radiation type.
PLAT002_ALERT_2_G Number of Distance or Angle Restraints on AtSite          16 Note
PLAT007_ALERT_5_G Number of Unrefined Donor-H Atoms .............            1 Report
PLAT092_ALERT_4_G Check: Wavelength Given is not Cu,Ga,Mo,Ag,In Ka     0.53400 Ang.
PLAT171_ALERT_4_G The CIF-Embedded .res File Contains EADP Records           5 Report
PLAT172_ALERT_4_G The CIF-Embedded .res File Contains DFIX Records           2 Report
PLAT173_ALERT_4_G The CIF-Embedded .res File Contains DANG Records           1 Report
PLAT174_ALERT_4_G The CIF-Embedded .res File Contains FLAT Records           1 Report
PLAT175_ALERT_4_G The CIF-Embedded .res File Contains SAME Records           1 Report
PLAT187_ALERT_4_G The CIF-Embedded .res File Contains RIGU Records           1 Report
PLAT301_ALERT_3_G Main Residue  Disorder .............(Resd  1  )          11% Note
PLAT302_ALERT_4_G Anion/Solvent/Minor-Residue Disorder (Resd  3  )        100% Note
PLAT302_ALERT_4_G Anion/Solvent/Minor-Residue Disorder (Resd  4  )        100% Note
PLAT304_ALERT_4_G Non-Integer Number of Atoms in ..... (Resd  3  )        7.49 Check
PLAT304_ALERT_4_G Non-Integer Number of Atoms in ..... (Resd  4  )        5.51 Check
PLAT398_ALERT_2_G Deviating  C-O-C    Angle From 120 for O3              105.2 Degree
PLAT802_ALERT_4_G CIF Input Record(s) with more than 80 Characters           1 Info
PLAT860_ALERT_3_G Number of Least-Squares Restraints ............          490 Note
PLAT883_ALERT_1_G No Info/Value for _atom_sites_solution_primary .      Please Do !
PLAT910_ALERT_3_G Missing # of FCF Reflection(s) Below Theta(Min).           1 Note
PLAT912_ALERT_4_G Missing # of FCF Reflections Above STh/L=  0.600           1 Note
PLAT933_ALERT_2_G Number of OMIT Records in Embedded .res File ...           4 Note
PLAT941_ALERT_3_G Average HKL Measurement Multiplicity ..........          3.4 Low
PLAT978_ALERT_2_G Number C-C Bonds with Positive Residual Density.           2 Info
PLAT984_ALERT_1_G The Pd-f'=   -2.5040 Deviates from the B&C-Value     -2.4885 Check
PLAT985_ALERT_1_G The Pd-f"=    0.6150 Deviates from the B&C-Value      0.6018 Check
```

0 **ALERT level A** = Most likely a serious problem - resolve or explain
0 **ALERT level B** = A potentially serious problem, consider carefully
11 **ALERT level C** = Check. Ensure it is not caused by an omission or oversight
26 **ALERT level G** = General information/check it is not something unexpected

4 ALERT type 1 CIF construction/syntax error, inconsistent or missing data

```
11 ALERT type 2 Indicator that the structure model may be wrong or deficient
 8 ALERT type 3 Indicator that the structure quality may be low
13 ALERT type 4 Improvement, methodology, query or suggestion
 1 ALERT type 5 Informative message, check
```

It is advisable to attempt to resolve as many as possible of the alerts in all categories. Often the minor alerts point to easily fixed oversights, errors and omissions in your CIF or refinement strategy, so attention to these fine details can be worthwhile. In order to resolve some of the more serious problems it may be necessary to carry out additional measurements or structure refinements. However, the purpose of your study may justify the reported deviations and the more serious of these should normally be commented upon in the discussion or experimental section of a paper or in the "special_details" fields of the CIF. checkCIF was carefully designed to identify outliers and unusual parameters, but every test has its limitations and alerts that are not important in a particular case may appear. Conversely, the absence of alerts does not guarantee there are no aspects of the results needing attention. It is up to the individual to critically assess their own results and, if necessary, seek expert advice.

Publication of your CIF in IUCr journals

A basic structural check has been run on your CIF. These basic checks will be run on all CIFs submitted for publication in IUCr journals (*Acta Crystallographica*, *Journal of Applied Crystallography*, *Journal of Synchrotron Radiation*); however, if you intend to submit to *Acta Crystallographica Section C* or *E* or *IUCrData*, you should make sure that full publication checks are run on the final version of your CIF prior to submission.

Publication of your CIF in other journals

Please refer to the *Notes for Authors* of the relevant journal for any special instructions relating to CIF submission.

PLATON version of 13/07/2021; check.def file version of 13/07/2021

Datablock 265K_4s_t9_decay - ellipsoid plot

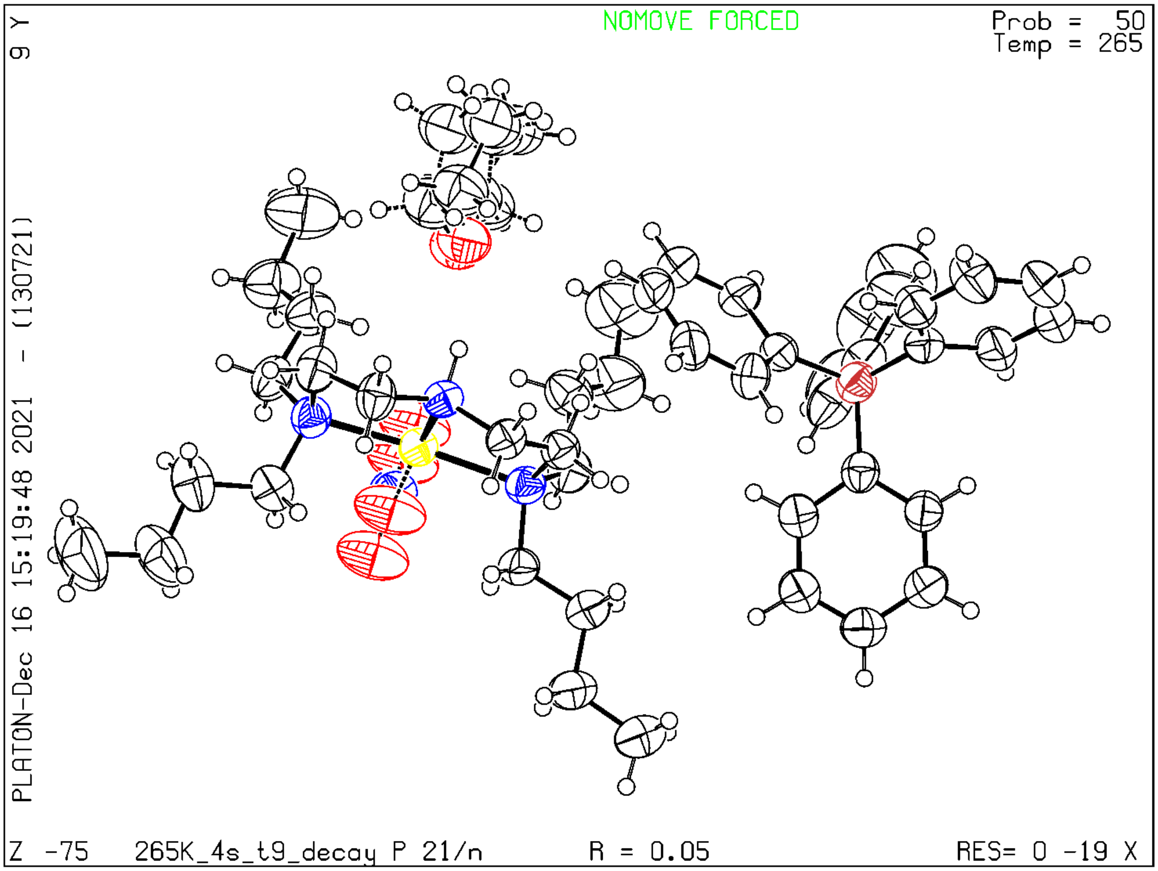

Supplement: Supplementary file 11 — Supplementary Data 2 [file 42004_2022_716_MOESM11_ESM.zip › Check-cifs/265K_4s_t9_decay_checkcif.pdf]
