# Supplementary material for: LED-pump-X-ray-multiprobe crystallography for sub-second timescales
Source: Commun Chem. 2022 Aug 26;5:102. doi: 10.1038/s42004-022-00716-1 (PMC9814726; doi:10.1038/s42004-022-00716-1)
Supplement: Supplementary file 1 — Raithby_PR File [file 42004_2022_716_MOESM1_ESM.pdf]

Peer Review File

LED-pump-X-ray-multiprobe crystallography for sub-second timescales

Open Access This file is licensed under a Creative Commons Attribution 4.0 International License, which permits use, sharing, adaptation, distribution and reproduction in any medium or format, as long as you give appropriate credit to the original author(s) and the source, provide a link to the Creative Commons license, and indicate if changes were made. In the cases where the authors are anonymous, such as is the case for the reports of anonymous peer reviewers, author attribution should be to 'Anonymous Referee' followed by a clear attribution to the source work. The images or other third party material in this file are included in the article's Creative Commons license, unless indicated otherwise in a credit line to the material. If material is not included in the article's Creative Commons license and your intended use is not permitted by statutory regulation or exceeds the permitted use, you will need to obtain permission directly from the copyright holder. To view a copy of this license, visit http://creativecommons.org/licenses/by/4.0/.

Reviewers' comments:

Reviewer #1 (Remarks to the Author):

This paper is very interesting and a pleasure to read. The authors have presented a very interesting work of pump-multiprobe single-crystal X-ray diffraction (SCXRD) technique for studying photoexcited solid-state species with millisecond-to-minute lifetime. In their control experiments, have shown the complete control of the pump-multiprobe sequencing and can access a range of timescales using the same setup and demonstrate our method by studying the variable-temperature kinetics of photo-activated linkage isomerism in [Pd(Bu4dien)(NO2)][BPh4] single-crystals. The ability of monitoring solid-state reactions in real-time contributes enormously to the understanding of structure-function relationships and assists in designing new materials. The ability to watch chemical and biological processes in 3D, and as they occur, opens up new vistas of chemical research.
More significantly, from the standpoints of characterization and structure confirmation, the authors should give a more critical analysis of the GS and ES diffraction data. Data analyses show,the data across multiple time-delays in each pump-probe cycle, which substantially shortens the experiment compared to previously-developed approaches and ensures that measurements at different time-delays are not unequally affected by cumulative crystal damage, photobleaching, and/or X-ray excitation.
Claims by the authors are thoughtfully set out and presented. In summary, the flexibility afforded by moving away from traditional imaging to event-counting detectors, such as the Timepix3 chip explored in this work, will be crucial for future time-resolved SCXRD studies at shorter timescales.
The following few points were not discussed in the manuscript. The authors might wish to respond as a means of fine-tuning the content, but I would not insist on changes as a requirement:
• On page 6, "The same behaviour is seen in all experiments, suggesting the same fundamental isomerisation mechanisms at all the timescales/temperatures studied." How about the Wilson plot for each experiment? Is the B factor kept constant at each temperature data set?
• On Page 14, the authors monitor the (-2 1 0) reflection, can provide the plot with the intensity vs the time?
• On page 10, "Online methods," I am confused here. What is the author trying to express in this section? May put this section in the supporting material or change to experimental section then will need to edit the whole article.

In summary, the paper is substantial in its scope, is significant in its findings, and in most respects meets the expectations of the Journal in content and quality.
My recommendation is therefore that the paper be accepted for publication as a Full-Length Article in Communications Chemistry, but only after show of the matters raised in my report.

Reviewer #2 (Remarks to the Author):

This paper, written by Hatcher, et al, describes a novel X-ray diffraction technique for studying photochemical reactions in single crystals using pulsed LEDs. The proposed photo-crystallography technique is a powerful method to instigate photochemical reactions in the time-window ranging from milliseconds to minutes, and the authors successfully applied the method to the study of linkage isomerism in a Pd complex. The effects of X-ray and LED exposure were carefully investigated.
In previous studies of photoisomerization of that kind, the discussion has only involved structures after continuous photo-illumination. However, the method implemented in this study facilitates elucidation of the reaction dynamics, and I think this is a very important study. This method can be applicable to the studies of various solid-state photoreactions. I think no revisions are needed but I have very minor comments: In Fig. 1, "Tseq" may be "Tcyc". In Fig. 5, label each graph (a-c).

Reviewer #3 (Remarks to the Author):

The article by Hatcher et al. treats about a novel approach in synchrotron photocrystallography which takes the advantage of the gated properties of the Pilatus detector. The group of Raithby and co-workers has already reported many pioneering results in the field of small molecule crystallography, especially these concerning reactions in crystals and metastable states characterisation. Here, the Authors follow their long time interest in the photoswichable nitro complexes of transition metals, and aim to monitor the izomerisation reaction process photocrystallographically step by step. The time-resolved photocrystallographic studies of small molecules are of high importance, yet they are very demanding. Time-resolved methods allow, in principle, to study molecule dynamics in the solid state, and as such contribute significantly to our understanding of photochemistry. In order to demonstrate the method's capabilities, the case of the earlier reported nitro Pd complex (studied already with the "static" photocrystallographic methods) was used as a proof of principle.

The current paper describes a new technique to study structure evolution at different time scales. The Authors start with describing the technique they use in details. The new method can be placed in between static ones (e.g. irradiation samples in situ at low temperatures on a laboratory diffractometer) and single pulse techniques allowing to study sub-nanosecond processes in crystals. The method of Hatcher et al., in the version with the Pilatus detector, is aimed at time scales from microseconds to even seconds. In general, the method is similar to the one published recently by Casaretto et al [ref. 27]. Nevertheless, there are some interesting improvements. Firstly, the Authors utilize the synchrotron radiation which allows them to record full structures at different time points (this was not possible before with an XPAD detector and a laboratory X-ray source (being, however, more available than synchrotrons)). Secondly, the Authors use the LEDs instead of lasers, which saves time and effort required to adjust the latter. Using the presented method the Authors were able to reconstruct the metastable nitrito state population build up and decay along with time. The experimental findings are supported by theoretical simulations and JMAK model fits. The manuscript is well written and very clearly presents different aspects of the method and experiments. Quantification of the metastable state lifetime at various temperatures is a solid piece of work. Nevertheless, the paper does not present much of a new chemical insight, e.g. on the mechanism of isomerisation, which would be achievable thanks to the new method, and would be very much interesting to the more general chemistry audience. Thus, in my opinion, the paper is more suitable to, for example, IUCrJ or related crystallographic journals. Other than that I suggest just a minor revision of the manuscript.

Below I list my more specific comments which, I believe, should be addressed prior to final decision on the paper.

1. In the introduction part the Authors compare their LED-based approach to single pulse techniques. The Authors say the LEDs might be more convenient here due to the larger conversion observed. However, in this discussion the Authors should mention that the single pulse methods are practically the only ones applicable to observe short-lived excited states which is supported by literature examples. The build-up of the population of the 'excited' states in contrast to the 'metastable' reaction products (although the distinction is rather blurry) is much more troublesome. The cumulative pumping method proposed previously by Fullagar and Coppens (DOI: 10.1107/S0909049500005422) poses some serious problems with sample heating. In this view, it would be valuable to estimate this factor using the Wilson-like plots as suggested some time ago by Coppens or Collet (e.g. DOI: 10.1107/S0108767309051046; 10.1107/S0108767310029429). Have the Authors tried that?

2. It would be interesting to compare the results concerning metastable state lifetimes with the results presented by Fournier and Coppens in their article on the topic (DOI: 10.1107/S0909049512010710).

3. Although the in-house method reported by Casaretto et al. is mentioned, I suggest including also

the developed method of chopper-based in house techniques, applied quite recently by Benedict and Coppens (e.g. DOI: 10.1063/1.4978240 or 10.1107/S160057671401961X) in the discussion.

4. In several literature cases multiple isomers are observed. In the reported experimental results only the endo-nitrito isomer is observed as a photo-induced reaction product. Do the Authors know whether it is formed directly, or why the exo-nitrito isomer is not detected / formed?

5. Why do the Authors ascribe a low-level background excitation to X-ray excitation (page 6)? What is the reasoning as this effect is rather unlikely? Is this effect also observed during an X-ray diffraction experiment of the GS structure of this system (using the same intensity synchrotron radiation, but with no LED irradiation)?

Minor comments:
1. In Figure 1 t(seq) (sequence?) should be changed to t(cyc).
2. On page 5 the Authors discuss the quality of refinements, but later the Rint values are given. Rint value though does not provide any indication of the refined model quality. I presume the Authors mean the overall quality of both the data and the refined structures. Please rephrase this fragment.
3. It would be valuable to add values of the maximal/minimal residual peaks in the photodifference maps.

Professor Paul R. Raithby FRSC

Professor of Inorganic Chemistry
BSc (Lond), PhD (Lond), ScD (Cantab)

Direct Line : +44 (0) 1225 383183
Facsimile : +44 (0) 1225 386231
Email : p.r.raithby@bath.ac.uk

Department of Chemistry

UNIVERSITY OF BATH

Claverton Down, BATH, BA2 7AY, UK

Tel : +44 (0)1225 386444
Email : p.r.raithby@bath.ac.uk

www.bath.ac.uk/chemistry

Reviewer 1:

(Reviewer comments in red italics):
This paper is very interesting and a pleasure to read. The authors have presented a very interesting work of pump-multiprobe single-crystal X-ray diffraction (SCXRD) technique for studying photoexcited solid-state species with millisecond-to-minute lifetime. In their control experiments, have shown the complete control of the pump-multiprobe sequencing and can access a range of timescales using the same setup and demonstrate our method by studying the variable-temperature kinetics of photo-activated linkage isomerism in [Pd(Bu$_4$dien)(NO$_2$)][BPh$_4$] single-crystals. The ability of monitoring solid-state reactions in real-time contributes enormously to the understanding of structure-function relationships and assists in designing new materials. The ability to watch chemical and biological processes in 3D, and as they occur, opens up new vistas of chemical research.
We thank the reviewer for their positive endorsement of our work and its importance.

More significantly, from the standpoints of characterization and structure confirmation, the authors should give a more critical analysis of the GS and ES diffraction data.

The GS and ES structure of complex **1** are previously reported and discussed in detail in an original publication (see *Hatcher, L. E. Raising the (metastable) bar: 100% photo-switching in [Pd(Bu4dien)(η^1-NO_2)]$^+$ approaches ambient temperature. CrystEngComm 2016, 18, 4180-4187*) and the crystals are used here as a benchmark material for proof-of-concept studies with our new time-resolved method.

We have added the following sentence to page 4, lines 2 – 3 of the manuscript to make this point clearer to the reader:

"Full details of the GS and photoinduced ES structures are discussed in detail previously,[29] and a summary of key data is included in the Supplementary Information, §1."

This time-resolved study does also uncover some new chemical insight for the switching behaviour of complex **1** at second-to-millisecond timescales, and we have added new paragraph to the Discussion section in the second paragraph of page 8 on this topic – please see the below responses to Reviewer 3 for further detail on this point.

Data analyses show, the data across multiple time-delays in each pump-probe cycle, which substantially shortens the experiment compared to previously-developed approaches and ensures that measurements at different time-delays are not unequally affected by cumulative crystal damage, photobleaching, and/or X-ray excitation.

Claims by the authors are thoughtfully set out and presented.

We thank the reviewer for their positive comments on the validity of our results and the quality of our presentation.

In summary, the flexibility afforded by moving away from traditional imaging to event-counting detectors, such as the Timepix3 chip explored in this work, will be crucial for future time-resolved SCXRD studies at shorter timescales.

We are pleased that the reviewer shares our view that event-counting detectors mark a key step forward into the future of time-resolved crystallographic research, as shown by our proof-of-concept studies in this work.

The following few points were not discussed in the manuscript. The authors might wish to respond as a means of fine-tuning the content, but I would not insist on changes as a requirement:

• On page 6, "The same behaviour is seen in all experiments, suggesting the same fundamental isomerisation mechanisms at all the timescales/temperatures studied." How about the Wilson plot for each experiment? Is the B factor kept constant at each temperature data set?

We thank the reviewer for this very sensible suggestion. Wilson plots have been generated for all pump-multiprobe data-sets, which show a strong linear fit and values for the temperature factor B (obtained from the slope of the graph) are constant (within error) for all time points obtained at a particular temperature. This strongly indicates that there is no significant deterioration of the crystal during the process. Example Wilson plots are made for two time-resolved datasets: one collected at 260 K (lowest temperature studied) and one at 284 K (highest temperature studied) and are included in the revised ESI document in Section 8.

The results are also consistent across the different time delays, proving that our pump-multiprobe method is successful at ensuring the measurements at different time-delays are not unequally affected by any of these processes – and indeed, the way the data is collected should preclude this issue as, for each time-delay, equivalent data-frames are collected at the same point in the experiment due to the pump-multiprobe strategy.

We have added 2 sentences to the Discussion on this topic on page 8, lines 7 – 11 of the revised manuscript, as follows:
"A comparison of the Wilson plots for all pump-multiprobe datasets confirms there is no significant deterioration of the crystal during the process, with all plots showing a strong linear fit and values for the temperature factor B constant (within error) for all time points obtained at a particular temperature. Example Wilson plots for the lowest (260 K) and highest (284 K) temperature experiments are included in Figure S29-30."

• On Page 14, the authors monitor the (-2 1 0) reflection, can provide the plot with the intensity vs the time?
The plot of intensity vs. time for the (-2 1 0) reflection is already provided in Figure 5 of the original manuscript.

• On page 10, "Online methods," I am confused here. What is the author trying to express in this section? May put this section in the supporting material or change to experimental section then will need to edit the whole article.
This issue arises from our original submission of this article to Nature Methods. As suggested by the reviewer, the "Online Methods" section has now been changed to a standard "Methods" section outlining the key experimental information, while the more extensive detail on the time-resolved diffraction experiments and other method development is moved to the Revised Supplementary Information file.

In summary, the paper is substantial in its scope, is significant in its findings, and in most respects meets the expectations of the Journal in content and quality.
My recommendation is therefore that the paper be accepted for publication as a Full-Length Article in Communications Chemistry, but only after show of the matters raised in my report.
We thank the reviewer for their positive recommendation.

Reviewer #2

This paper, written by Hatcher, et al, describes a novel X-ray diffraction technique for studying photochemical reactions in single crystals using pulsed LEDs. The proposed photo-crystallography technique is a powerful method to instigate photochemical reactions in the time-window ranging from milliseconds to minutes, and the authors successfully applied the method to the study of linkage isomerism in a Pd complex. The effects of X-ray and LED exposure were carefully investigated.
In previous studies of photoisomerization of that kind, the discussion has only involved structures after continuous photo-illumination. However, the method implemented in this study facilitates elucidation of the reaction dynamics, and I think this is a very important study. This method can be applicable to the studies of various solid-state photoreactions. I think no revisions are needed but I have very minor comments: In Fig. 1, "Tseq" may be "Tcyc". In Fig. 5, label each graph (a-c).
We thank the reviewer for their positive review of our work. We have revised Figure 1 to correct the typographic error highlighted.

Reviewer #3

The article by Hatcher et al. treats about a novel approach in synchrotron photocrystallography which takes the advantage of the gated properties of the Pilatus detector. The group of Raithby and co-workers has already reported many pioneering results in the field of small molecule crystallography, especially these concerning reactions in crystals and metastable states characterisation. Here, the Authors follow their long time interest

in the photoswichable nitro complexes of transition metals, and aim to monitor the izomerisation reaction process photocrystallographically step by step. The time-resolved photocrystallographic studies of small molecules are of high importance, yet they are very demanding. Time-resolved methods allow, in principle, to study molecule dynamics in the solid state, and as such contribute significantly to our understanding of photochemistry. In order to demonstrate the method's capabilities, the case of the earlier reported nitro Pd complex (studied already with the "static" photocrystallographic methods) was used as a proof of principle.
We thank the reviewer for their careful reading of our manuscript and for their assessment that the topic area is of high-importance.

The current paper describes a new technique to study structure evolution at different time scales. The Authors start with describing the technique they use in details. The new method can be placed in between static ones (e.g. irradiation samples in situ at low temperatures on a laboratory diffractometer) and single pulse techniques allowing to study sub-nanosecond processes in crystals. The method of Hatcher et al., in the version with the Pilatus detector, is aimed at time scales from microseconds to even seconds. In general, the method is similar to the one published recently by Casaretto et al [ref. 27]. Nevertheless, there are some interesting improvements. Firstly, the Authors utilize the synchrotron radiation which allows them to record full structures at different time points (this was not possible before with an XPAD detector and a laboratory X-ray source (being, however, more available than synchrotrons)). Secondly, the Authors use the LEDs instead of lasers, which saves time and effort required to adjust the latter. Using the presented method the Authors were able to reconstruct the metastable nitrito state population build up and decay along with time. The experimental findings are supported by theoretical simulations and JMAK model fits. The manuscript is well written and very clearly presents different aspects of the method and experiments. Quantification of the metastable state lifetime at various temperatures is a solid piece of work.
We thank the reviewer for their positive comments on the quality of our presentation and for their assessment that our makes improvements on the methods available for time-resolved crystallography methods.

Nevertheless, the paper does not present much of a new chemical insight, e.g. on the mechanism of isomerisation, which would be achievable thanks to the new method, and would be very much interesting to the more general chemistry audience. Thus, in my opinion, the paper is more suitable to, for example, IUCrJ or related crystallographic journals. Other than that I suggest just a minor revision of the manuscript.
The reviewer makes a good point that further chemical insight could be drawn from our results. As the original manuscript for this article was prepared for submission to Nature Methods, we concentrated on the methodology. For Communications Chemistry we now have the scope to broaden the chemical discussion and have added a new paragraph to the Discussion section of the Revised manuscript on page 8 as follows:
"*For model complex **1**, at the temperatures investigated (260 to 284 K) and the timescales that we access, we see only the known endo-nitrito-(η^1-ONO) isomer forming and no evidence of other transient linkage isomers. The system can thus be regarded as a simple binary switch that is "OFF" in the ground-state (GS) nitro-(η^1-NO_2) form and "ON" in the endo-nitrito-(η^1-ONO) form and has the potential to act as a 'near room temperature' molecular switch in opto-electronic applications. However, in other, related materials,[32-33] transient exo-nitrito-(η^1-ONO) isomers have been determined, but always at cryogenic temperatures far below those studied in by pump-multiprobe methods. Given the proven strong temperature-dependence of nitrite isomer lifetimes,[30] it is important to establish whether an exo-nitrito form might exist at potential operational temperatures and interfere with the simple binary switch. From our experimental observations it is likely that the exo-nitrito lifetime is much shorter than milliseconds near ambient temperature, explaining why it is not observed in this study. To confirm the existence of the exo-nitrito isomer as a possible transient intermediate in the nitro-(η^1-NO_2) to endo-nitrito(η^1-ONO) pathway, the Nudged Elastic Band (NEB) method was used to calculate the minimum energy pathway between the two known states (Supplementary Information §10). A local minima is found with an exo-nitrito geometry; however, the minima is shallow with only 6.6 kJ mol^{-1}*

required to continue to the endo-nitrito(η¹-ONO) compared to the expected activation energy of 114 kJ mol⁻¹ (Figures S32, S34) for the whole process. This indicates that this state will not be long-lived and so does not interfere with the simple binary switching of the isomers on the millisecond timescale near room temperature. This result compares well to the prior photocrystallographic evidence.[32-33] However, the NEB output does show that a considerable amount of movement is expected at the Pd(II) centre to accommodate the rearrangement of the nitrite ligand. This is interesting and compares well to the changes in electron density seen at the Pd(II) centre in the second- and millisecond-resolved molecular movies (Figure 4, Supplementary Information §7)."

We believe this highlights the more detailed chemical insight that can be gained from our method, both in this proof-of-concept study and for future investigations that utilise of our technique, making this article of interest to the wider chemistry audience of Communications Chemistry.
As part of this additional analysis, we conducted further calculations using the Nudged Elastic Band method implemented in ORCA. As such, we required further expertise and we add one new author, Dr Ben A. Coulson, to the author list.

Below I list my more specific comments which, I believe, should be addressed prior to final decision on the paper.
1. In the introduction part the Authors compare their LED-based approach to single pulse techniques. The Authors say the LEDs might be more convenient here due to the larger conversion observed. However, in this discussion the Authors should mention that the single pulse methods are practically the only ones applicable to observe short-lived excited states which is supported by literature examples. The build-up of the population of the 'excited' states in contrast to the 'metastable' reaction products (although the distinction is rather blurry) is much more troublesome. The cumulative pumping method proposed previously by Fullagar and Coppens (DOI: 10.1107/S0909049500005422) poses some serious problems with sample heating. In this view, it would be valuable to estimate this factor using the Wilson-like plots as suggested some time ago by Coppens or Collet (e.g. DOI: 10.1107/S0108767309051046; 10.1107/S0108767310029429). Have the Authors tried that?
We thank the reviewer for their suggestion to investigate the Photo-Wilson plots for our time-resolved data. We have completed this analysis for all of the pump-multiprobe time-resolved datasets collected for this manuscript and find that we obtain k_B values as follows: an average (mean) value of 1.069 ± 0.028 across all 12 datasets, with a maximum k_B of 1.187 ± 0.009 and a minimum k_B value of 0.987 ± 0.007 (i.e. a range of 0.200). Comparing these values with the literature (e.g. in the suggested Schmøkel *et al.* reference which states that a much higher k_B value of 1.70 indicates a significant temperature increase) our analysis suggests that the temperature increase as a result of the cumulative pumping with the LED sphere for several seconds is small, if not negligible, considering the errors on the values. There is also no significant trend in the values of k_B with irradiation time t_{exc}, which also supports our conclusion.
We have added the full analysis of the PhotoWilson plots to the Supplementary Information (Section 9) and included the following sentence on page 8 of the Revised Manuscript:
"*We confirm that the level of crystal heating from the LED sphere is low by determining the temperature scale factor k_B from PhotoWilson plots created for all pump-multiprobe experiments, following the method outlined by Coppens et al.[32] Full details of this analysis is provided in the Supplementary Information (§9). The plots show an average k_B value of 1.069 ± 0.028 across all 12 datasets that, when compared to the literature, indicates a negligible temperature rise in the crystal.[32]*"

2. It would be interesting to compare the results concerning metastable state lifetimes with the results presented by Fournier and Coppens in their article on the topic (DOI: 10.1107/S0909049512010710).
Unless we are mistaken, the DOI provided by the reviewer does not link to an article discussing metastable state lifetimes that we can compare to, instead it is a discussion of a methods for time-resolved Laue diffraction data processing? However, we do appreciate the importance of the point the reviewer is making.

It seems sensible to compare our results with those published for related materials, such as other linkage isomer systems. The most investigated of these at near-ambient conditions is the archetypal sodium nitroprusside (SNP) system. Woike et al. report lifetimes of ~300 ns for SNP single-crystals at room temperature (doi.org/10.1039/B924528F) and in the region of ~100 ms at 150 K (doi.org/10.1107/S2052520617009234), which are considerably shorter than our estimated values for the lifetime of complex **1** at similar temperatures (for example, at the highest temperature of 284 K investigated in this study, the rate constant for the ES decay (k_{dec}) = 2.42 × 10^{-1} s^{-1}, equating to a lifetime of ~ 5 s). This is perhaps to be expected when comparing the behaviour of M-NO with M-NO_2 linkage isomers at very low, cryogenic temperatures in steady-state photocrystallographic experiments. To thermally trap the M-ON or M-(ON) side-on isomers MS1 and MS2, this usually requires temperatures at or below 100 K, while ES Pd-ONO complexes such as **1** can be thermally trapped as high as 220-230 K (see DOI: 10.1039/C7CE01366C and 10.1039/C5CE02434J, for example). As such, we believe that our results are in-keeping with the reported literature on linkage isomer switching lifetimes.

3. Although the in-house method reported by Casaretto et al. is mentioned, I suggest including also the developed method of chopper-based in house techniques, applied quite recently by Benedict and Coppens (e.g. DOI: 10.1063/1.4978240 or 10.1107/S160057671401961X) in the discussion.

We thank the reviewer for highlighting this omission. DOI 10.1063/1.4978240 is now cited as the new reference [20] and the following addition has been made to the last sentence on page 2:

"Excepting a handful of examples using Laue methods to study species with microsecond lifetimes,[11-13] molecular crystallography has therefore continued to use monochromatic X-rays despite flux limitations, either at synchrotrons,[14-18] or more recently using some in-house time-resolved set-ups.[19-20]"

4. In several literature cases multiple isomers are observed. In the reported experimental results only the endo-nitrito isomer is observed as a photo-induced reaction product. Do the Authors know whether it is formed directly, or why the exo-nitrito isomer is not detected / formed?

No, we see no evidence of the exo-nitrito isomer at the timescales and temperatures we investigate in this study. From this evidence we can say that complex **1** does not display an exo-nitrito isomer with a lifetime longer than 400 ms in the temperature range 260 – 284 K. However, we cannot rule out that other isomers, including exo-nitrito, may be formed on a shorter timescale.

Our anecdotal evidence on exo-nitrito isomers in related Group 10 nitrite complexes is that we have tended to observe this ES isomer at lower (cryogenic) temperatures, but never at temperatures as high as 260 K. For example, in a study of a related complex $[Pd(Et_4dien)(NO_2)]^+$ (*Hatcher, L. E.; Raithby, P. R. The impact of hydrogen bonding on 100% photo-switching in solid-state nitro-nitrito linkage isomers. CrystEngComm 2017, 19, 6297-6304*), we observed long-lived metastable exo-nitrito isomers at small occupancy levels at 150 K, yet saw no evidence of these same isomers when photocrystallographic experiments were repeated at 200 K. This may indicate that the exo-nitrito lifetime drops off very quickly with temperature.

We have never yet observed evidence of exo-nitrito in any previous study of complex **1**, we have only ever observed the endo-nitrito ES even at 100 or 150 K. To supplement our chemical insight on this point, we conducted calculations using the Nudged Elastic Band method to determine the minimum energy pathway between the GS nitro and ES endo-nitrito forms (see our earlier discussion on this topic above). The calculated pathway indicates a local minimum with a geometry that matches the exo-nitrito arrangement. However, this minimum is fairly shallow, which would fit with an explanation that the exo isomer will be short-lived. This, combined with prior photocrystallographic evidence, would suggest that while the exo-nitrito isomer may well be a transient intermediate, at the temperatures studied in this work it has a lifetime much shorter than the minimum 400 ms time-resolution achieved.

5. Why do the Authors ascribe a low-level background excitation to X-ray excitation (page 6)? What is the reasoning as this effect is rather unlikely? Is this effect also observed during an X-ray diffraction experiment of the GS structure of this system (using the same intensity synchrotron radiation, but with no LED irradiation)?
Yes, X-ray excitation in complex **1** is seen for the GS structure of this system, as shown in the originally submitted version (see the Methods section, and the Supplementary Information (Section 3 in the Revised and original versions). Preliminary tests involving repeated data collections on the synchrotron source, in the dark but exposing the crystal to the same intensity synchrotron radiation, led to a small but measurable increase in the endo-nitrito-ONO isomer occupancy (Figures S4 and S5), confirming that the synchrotron X-rays induce excitation. A compromise was made by attenuating the synchrotron beam for all time-resolved diffraction studies to keep this X-ray excitation level to a manageable level (ca. 4%).

Minor comments:
1. In Figure 1 t(seq) (sequence?) should be changed to t(cyc).
A revised version of Figure 1 is included with the typographical error corrected.

2. On page 5 the Authors discuss the quality of refinements, but later the Rint values are given. Rint value though does not provide any indication of the refined model quality. I presume the Authors mean the overall quality of both the data and the refined structures. Please rephrase this fragment.
We have rephrased this sentence on page 5 of the Revised Manuscript as requested:
"Good-quality SCXRD data and structure refinements were obtained from all experiments, with residual factors of R_{int} = 9.16-9.85 % (mean 9.55 %) and R_1 = 6.24-6.41 % (mean 6.32 %) across the 9 datasets collected at the shortest t_{acq}."

3. It would be valuable to add values of the maximal/minimal residual peaks in the photodifference maps.
We have now included full details of the maximal/minimal values for the photodifference maps for Figure 4 in the caption for the Figures, as requested – this can be found in the Revised Manuscript.

For all photo-difference maps the level was set to a consistent ±0.8 e A^{-3} per contour, allowing us to make direct comparison between images. The maximum and minimum electron density levels in each image are as follows (in e A^{-3}): (a) min = -0.901, max = 1.729; (b) min = -2.023, max = 2.864; (c) min = -2.511, max = 4.087; (d) min = -2.838, max = 5.249; (e) min = -2.230, max = 3.657; (f) min = -0.975, max = 1.682; (g) min = -0.670, max = 0.783; (h) min = -0.666, max = 1.095.

****

We hope that, with these changes, our manuscript will now be suitable for publication. However, please do not hesitate to contact us should you require any further information. We look forward to hearing from you again in due course.

Yours sincerely,

Paul R. Raithby

Paul R. Raithby (on behalf of the authors)

REVIEWERS' COMMENTS:

Reviewer #1 (Remarks to the Author):

I am happy with the author's response. Therefore, I recommend that the paper be accepted for publication as a Full-Length Article in Communications Chemistry.

Reviewer #3 (Remarks to the Author):

I do appreciate a detailed answer to my comments given by the Authors in their rebuttal letter, as well as I am fully satisfied with the additions made in the article and supporting materials, and by the enriched discussion of the results. I find the computational part regarding the isomerization reaction pathway especially interesting, as well as linking the changes in electron density seen at the Pd(II) centre in the second- and millisecond-resolved molecular movies with the considerable amount of movement expected at the Pd(II) centre to accommodate the rearrangement of the nitrite ligand according to the NEB output. Just to explain- regarding the paper by Fournier and Coppens – I have rather thought of using their method to estimate the metastable state lifetimes for the Pd system and compare these with the ones obtained by the Authors using their approach. However, given the amount of work and details risen by the Authors in the current version of the manuscript, I do not see now the point to further extend the discussion.
To conclude - in my opinion, the paper by Hatcher et al. is now suitable for publication in the Communications Chemistry journal without further delay.
